# Characterizing the immune response to *Mycobacterium tuberculosis*: a comprehensive narrative review and implications in disease relapse

**DOI:** 10.3389/fimmu.2024.1437901

**Published:** 2024-11-22

**Authors:** Fatima Rahman

**Affiliations:** ^1^ Department of Pharmacology, University College London, London, United Kingdom; ^2^ Istituto per le Applicazioni del Calcolo, Consiglio Nazionale delle Ricerche, Rome, Italy

**Keywords:** immune response, tuberculosis, adaptive immunity, innate immunity, granuloma, relapse

## Abstract

**Introduction:**

Tuberculosis remains the leading cause of death from infectious diseases among adults worldwide. To date, an overarching review of the immune response to *Mtb* in humans has not been fully elucidated, with innate immunity remaining poorly understood due to historic focus on adaptive immunity. Specifically, there is a major gap concerning the contribution of the immune system to overall bacterial clearance, particularly residual bacteria. This review aims to describe the time course of interactions between the host immune system and *Mtb*, from the start of the infection to the development of the adaptive response. Concordantly, we aim to crystallize the pathogenic effects and immunoevasive mechanisms of *Mtb*. The translational value of animal data is also discussed.

**Methods:**

The literature search was conducted in the PubMed, ScienceDirect, and Google Scholar databases, which included reported research from 1990 until 2024. A total of 190 publications were selected and screened, of which 108 were used for abstraction and 86 were used for data extraction. Graphical summaries were created using the narrative information (i.e., recruitment, recognition, and response) to generate clear visual representations of the immune response at the cellular and molecular levels.

**Results:**

The key cellular players included airway epithelial cells, alveolar epithelial cells, neutrophils, natural killer cells, macrophages, dendritic cells, T cells, and granulomatous lesions; the prominent molecular players included IFN-γ, TNF-α, and IL-10. The paper also sheds light on the immune response to residual bacteria and applications of the data.

**Discussion:**

We provide a comprehensive characterization of the key immune players that are implicated in pulmonary tuberculosis, in line with the organs or compartments in which mycobacteria reside, offering a broad vignette of the immune response to *Mtb* and how it responds to residual bacteria. Ultimately, the data presented could provide immunological insights to help establish optimized criteria for identifying efficacious treatment regimens and durations for relapse prevention in the modeling and simulation space and wider fields.

## 1 Introduction

Tuberculosis (TB) is the primary cause of death from infectious diseases among adults worldwide. Each year, more than 10 million people are newly infected (1). The causative agent, *Mycobacterium tuberculosis* (*Mtb*), is a prototroph (2) that operates an erratic and highly repetitive life cycle, traversing various niches and physiological states—a feature unique to this pathogen and therefore its longevity in humans (3).

As with most infections, the immune system forms the first line of defense against *Mtb* and contributes to both infection prevention and control. The interaction between *Mtb* and the host results in a complex and multifaceted immune response, which can lead to clinical TB, subclinical TB, or clearance (i.e., non-disease state) (5). Under the new classification, there are four disease states; these include both clinical and subclinical diseases, which can be either infectious or non-infectious. For clinical TB, the non-infectious state includes all forms of disease whereby the individual recognizes the symptoms sufficiently to seek care; the infectious state is defined as individuals who are infectious based on sputum microbiologically confirmed pulmonary TB, and the individual recognizes the symptoms sufficiently to seek care. For subclinical TB, the non-infectious state is defined by the presence of macroscopic pathology while the patient is not infectious, and symptoms are not recognized by the individual or are insufficient to seek care. Finally, the subclinical infectious state is classified by the capacity for *Mtb* transmission with macroscopic pathology present, although any symptoms are also not recognized by the individual or are insufficient to seek care. Moreover, the fifth non-disease state is defined as the presence of viable *Mtb* in the host that is effectively contained by the immune response, whereby the individual does not present any macroscopic pathology of symptoms and is non-infectious (6).

A remarkable aspect of TB is that infection with *Mtb* rarely causes active disease (4). Among patients who fail to clear the bacteria, only 5% progress to active disease, while 95% can contain the pathogen via adaptive immunity (4, 7). However, there have been case-contact studies that indicate early clearance of bacteria by the innate response, independent of adaptive immunity, in up to 55% of cases, although this has yet to be proven definitively (8).

Currently, relapse and drug resistance are the two major challenges in the treatment of TB due to inadequate treatment outcomes. In clinical TB, relapse is the recurrence of TB in a patient who has been deemed cured (9). This may result from endogenous persisting infection by the same *Mtb* strain that caused the previous disease episode, as one may not become immunized against the pathogen, or re-infection with a different bacterial strain (9). In TB infection (TBI), approximately 5%–10% of individuals later develop active disease owing to reactivation (4). In summary, relapse provides the formative context for this review.

To understand relapse, we must first appreciate the presence of different bacterial phenotypes in TB (**Supplementary Figure 1**). Following infection, *Mtb* undergoes multiple rounds of rapid replication, which is then slowed or arrested by host immunity (2, 3). Thus, TB disease states are characterized by rapidly replicating, slowly replicating, and non-replicating bacterial phenotypes (3). In relapse, it is the non-replicating, slowly replicating (persisters), and drug-resistant bacteria that circumvent clearance. That is, they can evade the antimicrobial activity of both drugs (10) and the immune system—the latter of which represents a significant gap in the drug development purview.

Dovetailing with relapse is drug resistance, which is caused by exposure of pathogens to low, inefficacious doses of antitubercular drugs (11). For drug-susceptible TB, isoniazid (H), rifampicin (R), pyrazinamide (Z), and ethambutol (E) are used as combination therapy for 6 months (2HRZE/4HR) (12). Combination therapy is used to combine both bactericidal and sterilizing drugs, with adequate duration, to enhance antimicrobial efficacy and prevent drug resistance (12). An additional benefit of combination therapy is that it lowers disease relapse compared to monotherapy (13, 14). However, it is noteworthy that 20% of patients even on short-course therapy (4 months) develop relapse (15). For multidrug-resistant TB (MDR-TB) and rifampicin-resistant TB (RR-TB), the World Health Organization (WHO) suggests a 6-month regimen of bedaquiline, pretomanid, linezolid, and moxifloxacin (BPaLM) rather than the 9-month and longer regimens. This applies to pulmonary TB and extrapulmonary TB, except for TB involving the central nervous system, miliary TB, and osteoarticular TB. Further, for patients who are not eligible for BPaLM, the WHO recommends the use of the 9-month all-oral regimen in individuals with MDR-TB, individuals with RR-TB, and those without resistance to fluoroquinolones (16, 17). This involves an intensive phase with bedaquiline, levofloxacin or moxifloxacin, ethionamide or linezolid, ethambutol, isoniazid, pyrazinamide, and clofazimine, and then a continuation phase with fluoroquinolones, clofazimine, ethambutol, and pyrazinamide (17).

However, low patient compliance has resulted in increased resistance (18). See **Supplementary Material** for further details. Consequently, shortening of treatment duration has become a major goal of TB drug development (19). However, empirical attempts (20) to reduce the continuation phase for drug-susceptible TB treatment to less than 4 months have caused relapse rates as high as 40%, depending on the drug combination (9). It is anticipated that improved clarity on the role of the immune system in TB to support modeling and simulation (M&S) efforts will convey a deeper understanding of the mechanisms that contribute to relapse and may help identify prevention strategies.

Thus, arguably, while new compounds are in the pipeline (21), a more integrated approach to advance drug development may involve highlighting the extent to which drugs and the immune system contribute to bacterial clearance, in the context of pathogenic immune evasion (i.e., host–pathogen–drug interactions). This may support translational and quantitative clinical pharmacology efforts aimed at better elucidating the mechanisms that may cause and prevent relapse. However, the characterization of the immune response to *Mtb* has been largely evaded in clinical research. That is, the systematic consideration of the time course of immune response—discerning the innate and adaptive phases—as a contributor to bacterial clearance in TB has not been fully elucidated.

While this gap has been acknowledged by various research groups, an overarching review of the immune response to *Mtb* has not been fully expounded, with the innate arm remaining poorly understood due to the historic focus on adaptive immunity (22). Specifically, the tripartite host–pathogen–drug interaction, considering the time course of response from infection to granuloma formation, is yet to be examined. This is imperative in research, as the immune system is a determinant of bacterial clearance before, during, and after drug treatment.

## 2 Aims

This review aims to provide a comprehensive characterization of the key immune players that are implicated in pulmonary TB, in line with the organs or compartments in which they reside, as a contributor to bacterial clearance—discerning the innate and adaptive arms (at the cellular and molecular levels). We explore the intricate interplay of molecular mediators (e.g., cytokines and chemokines) and signaling pathways involved in orchestrating the immune response to *Mtb*. Bacterial persistence through immunoevasion is also highlighted. Further, we provide the time course of the immune system–pathogen interactions from the start of the infection to the development of the adaptive response in animal models. This review also sheds light on the limited role of the immune system in eradicating residual persister bacteria. While there is extensive research in this field, currently, there is no report that describes the entire time course of the immune response to *Mtb*. The translational value of animal data is also discussed. Ultimately, we aim to provide valuable insights into TB immunology as a basis for the development of translational *in silico* models, a tool that integrates quantitative clinical pharmacology principles (i.e., pharmacokinetics and pharmacodynamics) and host–pathogen–drug interactions to identify shortened, efficacious drug combinations/regimens for relapse prevention, to help elucidate the mechanisms that may cause relapse, and to predict the risk of relapse.

## 3 Methods

For the literature search, a narrative approach was employed. Publications in English and French were selected from the PubMed, Science Direct, and Google Scholar databases throughout the period of 1990 to 2024.

The first literature search included the MeSH terms “tuberculosis” and “granuloma”, paired with the keywords “immun*” and “cell” while excluding the terms “bovis” and “avium”. To provide a completed representation of immune response, a further two search methods were employed: one was bibliographic mining, and the other was an *ad hoc* search using the MeSH term “tuberculosis” with more specific keywords such as “airway epithelial cell”, “alveolar epithelial cell”, “neutrophil”, “natural killer”, “macrophage”, “dendritic cell”, “CD4+ T cell”, and “CD8+ T cell” for cellular data; and “*in silico*”, “quantitative”, “computational model”, and research groups such as “Kirschner” to provide reference to *in silico* models; and keywords like “T cell subsets” and “immune cell migration” for broader immunological insights. Finally, a narrative synthesis of the papers was conducted while exploring the consistency in key data as a measure of reliability and validity. Furthermore, figures were created using the narrative information (i.e., recruitment, recognition, and response) to generate a clear visual representation of the signaling and immune mechanisms per player at the cellular and molecular levels using a visualization tool (BioRender, BioRender.com).

**Figure 1:**
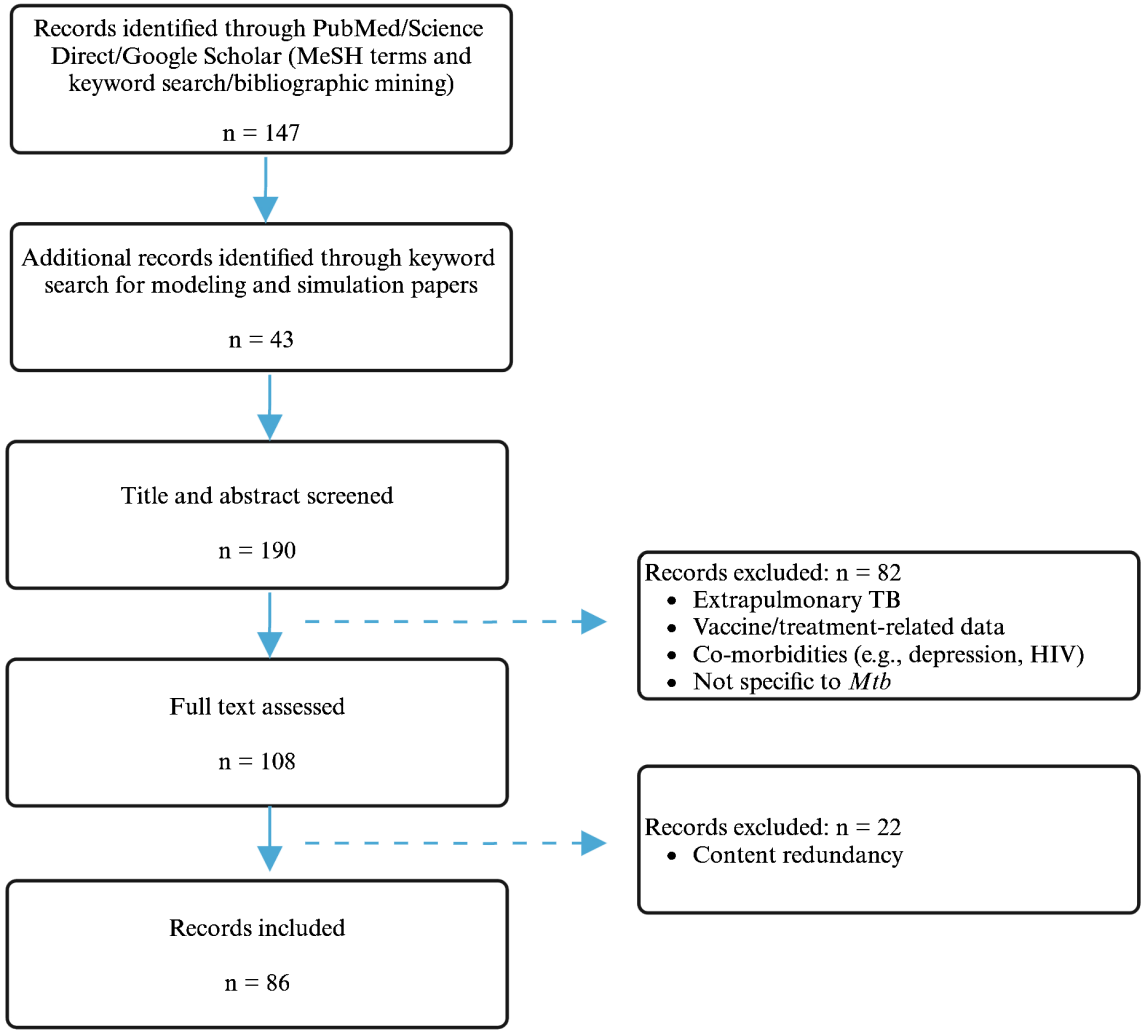
Schematic representation of the literature search process. Created with BioRender.com.

A total of 190 publications were selected and screened, of which 108 were used as sources for abstraction and 86 were used for data extraction. The literature search flowchart is shown in **Figure 1**. A summary of the papers used for extraction per immune player is shown in **Table 1**. This was created by synthesizing a high-level summary of the most crucial data that were extracted per immune player, per paper.

**Table 1:** Summary of major papers and data extracted.

Player/component	Site	Major references	Data extracted
**Airway epithelial cells**	Airways	Chai et al., 2020 (40)	Goblet cells and mucous cells; plasma cells and antibodies produced; innate lymphoid cells; innate immune T cells; MAIT cells; antigen recognition receptors; structure and function
		Li et al., 2012 (44)	AEC role in innate and adaptive arms; antigen presentation to T cells (non-professional)
		Gupta et al., 2018 (22)	Recruitment: receptors, signaling pathways, and cytokines released
		Rodrigues et al., 2020 (45)	Airway epithelium cell surface area
		Condon et al., 2011 (46)	Cytokines that modulate intraepithelial cells
		Barclay et al., 2023 (47)	Neutrophil and APC (DC) recruitment
		Vono et al., 2017 (48)	Antigen presentation
**Alveolar epithelial cells**	Lungs	Rodrigues et al., 2020 (45)	Major surface area for inhaled pathogens; macrophage and DC function in the alveolar space
		Chai et al., 2020 (40)	Alveolar epithelial cell phenotype; antigen recognition receptors
		Li et al., 2012 (44)	ATI and ATII; antigen presentation; innate cellular response; bacterial replication in alveoli
		Bussi and Gutierrez, 2019 (49)	Alveolar response to bacilli that pass AECs
		Gammack et al., 2004 (50)	Early macrophage and DC activities
		Corleis and Dorhoi, 2020 (30)	Type II pneumocytes recruit neutrophils
		Gupta et al., 2018 (22)	Immunoregulation by cytokines
		Nisa et al., 2022 (51)	Necrosis
**Neutrophils**	Lungs	Liu et al., 2017 (31)	The complex role of neutrophils; not MHC-restricted; *Mtb* cell wall components as ligands for receptors; recruitment to lungs; respiratory burst; neutrophil-mediated damage to host parenchyma
		Sokol and Luster, 2015 (52)	Recruitment from bone marrow and inflammatory stimuli needed
		Lowe et al., 2012 (53)	Neutrophil abundance and infiltration; stimuli for recruitment to lungs; priming and extravasation; internalization mechanisms
		Muefong et al., 2022 (54)	Recognition: PAMPs and DAMPs
		Mak and Saunders, 2006 (55)	Opsonization of antigen
		Pennisi et al., 2019 (37)	Apoptosis and necrosis
		Gupta et al., 2018 (22)	Chemokines released neutrophils
		Nisa et al., 2022 (51)	Apoptosis; NET immune cell recruitment and activation
		Dallenga et al., 2017 (56)	Efferocytosis vs. necrotic neutrophils
		Borkute et al., 2021 (57)	Phagocytosis
		Muefong and Sutherland, 2020 (58)	Oxidative burst, tissue injury via NETosis
		Mattila et al., 2013 (42)	Neutrophil cytokines and cross-priming of CD8+ T cells
		Hilda et al., 2020 (59)	Neutrophil response to sensing *Mtb*
		Li et al., 2019 (60)	Antigen presentation capabilities
**Natural killer cells**	Lungs	Gupta et al., 2018 (22)	Intracellular pathogen killing; mediation of action by cytokines
		Qin et al., 2023 (61)	NK cell abundance in lungs
		Nutt and Huntington, 2019 (35)	Bacterial killing pathways (apoptosis; perforin/granzyme)
		Sokol and Luster, 2015 (52)	Recruitment via chemokines
		Liu et al., 2017 (31)	Not MHC-restricted; *Mtb* cell wall components as ligands for receptors
		Nisa et al., 2022 (51)	Bacterial killing pathways (apoptosis; perforin/granzyme)
		Lin and Flynn, 2015 (62)	Antigen recognition; role of granulysin
		Murphy et al., 2022 (63)	Role of perforin and granzymes
**Macrophages**	Lungs and lymph nodes	Chai et al., 2020 (40)	Macrophage phenotypes; macrophage response
		Corleis and Dorhoi, 2020 (30)	Macrophage origin
		Weiss and Schaible, 2015 (33)	Resident macrophages; macrophage response
		Chandra et al., 2022 (64)	AM migration from alveolar space to lung interstitium
		Ganguli et al., 2005 (4)	Macrophage recruitment
		Gammack et al., 2004 (50)	Macrophage chemotaxis; macrophage response
		Sokol and Luster, 2015 (52)	Macrophage recruitment
		Liu et al., 2017 (31)	Macrophage response
		Guirado et al., 2013 (65)	Macrophage response
		Chaurasiya, 2018 (66)	Macrophage response
		Nisa et al., 2022 (51)	Necrotic dissemination of bacteria
		Pennisi et al., 2019 (37)	Macrophage response
		Cilfone et al., 2015 (67)	Macrophage response
		Ryndak and Laal, 2019 (39)	Macrophage response (intracellular bacterial killing)
		Pedruzzi et al., 2016 (68)	Macrophage response
		Lowe et al., 2012 (53)	Macrophage response
		Wigginton and Kirschner, 2001 (69)	Macrophage response
		Sud et al., 2006 (34)	Macrophage response
**Dendritic cells**	Lungs and lymph nodes	Chai et al., 2020 (40)	Sentinel cells
		Condon et al., 2011 (46)	Lung homeostasis; DC recruitment; antigen recognition; RME
		Kim and Shin, 2022 (70)	DC origins
		Sia et al., 2015 (71)	Antigen presentation; antigen recognition
		Marino and Kirschner, 2004 (72)	Antigen presentation; DC recruitment; DC response
		Stillwell, 2016 (73)	DC recruitment
		Marino et al., 2011 (74)	DC response
		Pennisi et al., 2019 (37)	DC response
		Lian and Luster, 2015 (76)	DC response
		Sokol and Luster, 2015 (52)	DC response
		Marino et al., 2004 (75)	DC response
		Chandra et al., 2022 (64)	DC response; localization
		Cilfone et al., 2015 (67)	DC response
		Lin and Flynn, 2015 (62)	DC response
**CD4+ T cells**	Lungs and lymph nodes	Marino and Kirschner, 2004 (72)	Antigen presentation to naïve CD4+ T cells; Th1 cell differentiation; Th2 cell differentiation; CD4+ T-cell migration; Th1 effector functions; Th2 effector functions
		Carty et al., 2018 (77)	Th2 cell differentiation; Th2 effector functions; Th17 cell differentiation; Th17 effector functions
		Nutt and Huntington, 2019 (35)	Naïve T cells
		Wigginton and Kirschner, 2001 (69)	T-cell main roles; Th1 cell differentiation; Th2 cell differentiation; Th1 effector functions
		Bozzano et al., 2014 (78)	Importance of CD4+ and CD8+ T cells to prevent TB
		Kuka et al., 2019 (82)	Antigen presentation to naïve CD4+ T cells; Th1 cell differentiation; Th2 cell differentiation; Th1 effector functions
		Chaurasiya, 2018 (66)	Th1 cell differentiation; Th1 effector functions
		Gupta et al., 2018 (22)	Th1 cell differentiation
		Leal Rojas et al., 2017 (83)	Th1 cell differentiation; Th17 cell differentiation
		Khader et al., 2006 (84)	Th1 cell differentiation
		Huang et al., 2012 (85)	Th17 cell differentiation; Th17 effector functions
		Lourenço and La Cava, 2011 (86)	iTreg cell differentiation
		Herbert et al., 2017 (87)	iTreg cell differentiation
		Pennisi et al., 2019 (37)	CD4+ T-cell migration
		Sokol and Luster, 2015 (52)	CD4+ T-cell migration; Th2 effector functions
		Flynn and Chan, 2022 (79)	Th1 effector functions
		Guzzetta and Kirschner, 2013 (121)	Th1 effector functions
		Murphy et al., 2022 (63)	Th1 effector functions
		Cilfone et al., 2015 (67)	Th1 effector functions
		Guirado et al., 2013 (65)	Th2 effector functions
		Liu et al., 2017 (31)	Th17 effector functions
**CD8+ T cells**	Lungs and lymph nodes	Seder and Ahmed, 2003 (80)	CD8+ T-cell differentiation
		Capece and Kim, 2016 (81)	Shorter activation of CD8+ T cells
		St. Paul and Ohashi, 2020 (88)	CD8+ T-cell differentiation; Tc1 effector functions; Tc2 effector functions
		Pennisi et al., 2019 (37)	CD8+ T-cell migration
		Gammack et al., 2004 (50)	CD8+ T-cell migration
		Sokol and Luster, 2015 (52)	CD8+ T-cell migration
		Wigginton and Kirschner, 2001 (69)	Tc1 effector functions
		Murphy et al., 2022 (63)	Tc1 effector functions
		Sud et al., 2006 (34)	Tc1 effector functions
		Lin and Flynn, 2015 (62)	Tc1 effector functions
		Kudryavtsev et al., 2023 (122)	Tc1 effector functions; Tc2 effector functions
		Guirado et al., 2013 (65)	Tc2 effector functions
		Carty et al., 2018 (77)	Tc2 effector functions
**Granuloma**	Lungs and lymph nodes	Linderman et al., 2015 (37)	Host–microbe interactions; granuloma formation; granuloma types
		Pagán and Ramakrishnan, 2015 (89)	Granuloma formation
		Marino et al., 2018 (41)	Granuloma function
		Dooley et al., 2016 (90)	Lesion types
		Cronan, 2022 (91)	Granuloma heterogeneity; granuloma formation; macrophage differentiation; necrotic cell death; T-cell function
		Linderman and Kirschner, 2015 (92)	Granuloma dual functions (bacterial control vs. bacterial survival); granuloma formation
		Schaaf and Zumla, 2009 (93)	Granuloma formation; granuloma types
		Marino et al., 2011 (74)	Granuloma formation
		Russell et al., 2009 (94)	Granuloma formation; granuloma types
		Lin and Flynn, 2015 (62)	Granuloma formation
		Ramakrishnan, 2012 (32)	Granuloma formation; granuloma types
		Flynn et al., 2011 (25)	Granuloma formation; granuloma types
		Chai et al., 2020 (40)	Granuloma formation
		Huang et al., 2019 (36)	Granuloma formation; macrophage differentiation
		Hunter et al., 2022 (28)	Granuloma formation; multinucleated giant cells
		Gago et al., 2018 (95)	Granuloma formation
		Lanni et al., 2023 (96)	Granuloma formation
		Gideon et al., 2015 (26)	Granuloma formation
		Liu et al., 2017 (31)	Granuloma formation
		Huynh et al., 2011 (97)	Granuloma formation
		Evans et al., 2020 (98)	Granuloma formation; fibrosis and calcification
		Ehlers and Schaible, 2012 (99)	Granuloma formation; granuloma types
		Hortle and Oehlers, 2020 (100)	Granuloma formation; fibrosis
		Mattila et al., 2013 (42)	Granuloma formation; granuloma types
		Nisa et al., 2022 (51)	Granuloma types; lipid droplets
		Warner, 2014 (2)	Granuloma types
		Sawyer et al., 2023 (29)	Necrotizing granuloma formation; non-necrotizing leukocyte aggregates
		Gong et al., 2015 (124)	Granuloma formation; fibrosis/calcification
		Warsinske et al., 2017 (125)	Granuloma types
		Silva Miranda et al., 2012 (123)	Granuloma types
**Bacteria**	Lungs and lymph nodes	Warner, 2014 (2)	Aerobic vs. hypoxic niches
		Marino and Kirschner, 2016 (101)	*Mtb* is preferentially an intracellular pathogen
		Marino et al., 2011 (74)	*Mtb* slow division rate
		Weiss and Schaible, 2015 (33)	*Mtb* immune evasion mechanisms
		Upadhyay et al., 2018 (102)	*Mtb* immune evasion mechanisms
		Gago et al., 2018 (95)	*Mtb* immune evasion mechanisms; aerobic vs. hypoxic niches; bacterial manipulation in granuloma
		Lowe et al., 2012 (53)	*Mtb* immune evasion mechanisms
		Sia et al., 2015 (71)	*Mtb* immune evasion mechanisms
		Ehrt et al., 2018 (3)	Aerobic vs. hypoxic niches
		Huynh et al., 2011 (97)	Bacterial manipulation in granuloma
		Pagán and Ramakrishnan, 2015 (89)	Bacterial manipulation in granuloma
		Mahajan et al., 2012 (103)	Bacterial manipulation in granuloma

AEC, airway epithelial cell; APC, antigen-presenting cell; DC, dendritic cell; ATI, type I alveolar epithelial cells; ATII, type II alveolar epithelial cells; PAMPs, pattern-associated molecular patterns; DAMPs, damage-associated molecular patterns; NET, neutrophil extracellular trap; AM, alveolar macrophage; RME, receptor-mediated endocytosis.

The inclusion criteria comprised the following: human data, *in vivo* models [mice and non-human primates (NHPs)], *in vitro* models, *in silico* models, models specific to *Mtb*, presence of pulmonary TB, and presence of immune response. The exclusion criteria comprised models based on extrapulmonary TB, research related to treatment, the presence of co-morbidities, and models that were non-specific to *Mtb*.

The key data extracted to characterize the immune system response to *Mtb* comprised the players involved, the key mediators involved, and temporal data spanning 12 weeks to describe both the innate and adaptive phases.

For the “Time course of immune response”, the innate and adaptive phases were described using animal models (mice and NHPs) given the paucity of temporal data in humans. As mice are the primary animal species utilized for the preclinical development of antitubercular drugs due to their manageability, low cost, and susceptibility to *Mtb* (23), we considered it appropriate to use the same animal species to define the time course of the immune response to reliably ascribe bacterial clearance to the immune system instead of species differences. However, it should be noted that while mice do develop granulomas (24), murine lungs lack the structured and organized appearance of human lesions (25). To reconcile these differences, data were obtained from NHP models whose lesions demonstrate a closer pathological approximation to humans (24). NHP data were therefore used to describe the timing of granuloma formation and response. See the “Discussion” section for details on the translation of preclinical findings to humans.

For the “Innate immune response” section, the data are presented according to the following structure per immune player: background, recruitment (to infection site), recognition (of *Mtb*), and response. For the “Adaptive immune response” section, the T-cell data are presented in the following structure: background, T-cell differentiation, T-cell migration to lungs, and T-cell effector functions (for both CD4+ and CD8+ T cells). The “Granuloma” section is presented as follows: background, formation, and granuloma types. Finally, the “Bacteria” section is structured as follows: background, *Mtb* immunoevasive mechanisms, aerobic versus hypoxic niches, and bacterial manipulation in the granuloma. These sections are complemented by available data on the compartments within which these processes take place (blood, lymph nodes, or lungs). Following the immune response data, the “Immune response and implications in disease relapse” and “Application of the data” sections are presented.

Of note, to elucidate granuloma immunology, sole reliance on human or animal data would not be prudent, as no single model is comprehensive. To effectively research human granuloma formation and response, lung tissue containing these lesions must be extracted, which is extremely challenging (26) due to ethical constraints surrounding lung autopsies (27). Obtaining lung samples via biopsy is also limited, given that symptoms may not present for months to years (28) (see the “Discussion” section for further details). Moreover, there are little data on how granulomas contain *Mtb* and the spatial organization of immune cells at the border of necrotizing regions (29). Thus, the most judicious alternative was to use animal models that closely recapitulate human disease (26). To date, NHPs (specifically macaques) have shown remarkable similarity to human infection (26), as they have similar physiology, anatomy, and response to *Mtb* infection, encompassing the entire spectrum of the disease and immunology in humans (28). Therefore, for the “Granuloma” section, a systems biology approach was taken to provide a fuller depiction of response.

Definitions of the icons used to represent each cellular player (**Supplementary Figure 2**), including the colors and arrows (i.e., mediators and processes) in the figures (**Supplementary Figure 3**), are provided. A high-level summary of the role of each player in the innate and adaptive phases is provided in **Supplementary Figures 4**, **5**.

A tabular summary of the innate, adaptive, and granuloma response with the associated mediators, category of molecule, and site of action is provided in **Supplementary Table 1**. A more detailed overview ranking the players and mediators by their importance in the immune response against *Mtb* is provided in **Supplementary Tables 2**, **3**, respectively.

This review was comprehended under expert guidance, and the tasks were conducted independently in a stepwise process. The initial step was to develop a “storyboard” detailing the immune response to *Mtb* from the start of infection to granuloma formation. This step involved using a reference management software, Zotero, to open the PDF versions of the selected papers for each immune player. Relevant data that consistently appeared across these papers were extracted, and the narrative synthesis of the response was conducted simultaneously.

Subsequently, the figures were created in BioRender. The process began with finding the relevant icons for each immune player and undertaking training provided by BioRender.com on creating effective scientific images. This knowledge was applied, and the narrative text for each player was followed to create graphical representations of the immune response iteratively. The highly organized structure of the narrative text (i.e., background, recruitment, response) was crucial in ensuring a logical flow and compartmentalization in the figures.

After creating the storyboard and the initial versions of the figures, a tabular summary of the collated data (**Supplementary Tables**) was created to facilitate navigation for readers. This involved reading each immune player section and then extracting specific data (i.e., copying and pasting the cellular players and their subsets) into an Excel spreadsheet in a list format, followed by aligning these with the molecular players associated with each cellular player within this spreadsheet. Information on the type of molecule and site of action was also added to provide a broader picture. The same method was used to create ranking spreadsheets for each player and each molecule. The data were then copied and pasted into a Word file from the Excel spreadsheet. They were also fact-checked against the source. Multiple iterations of all review subparts were developed to allow for corrections and necessary adjustments.

## 4 Time course of immune response

**Figure 2:**
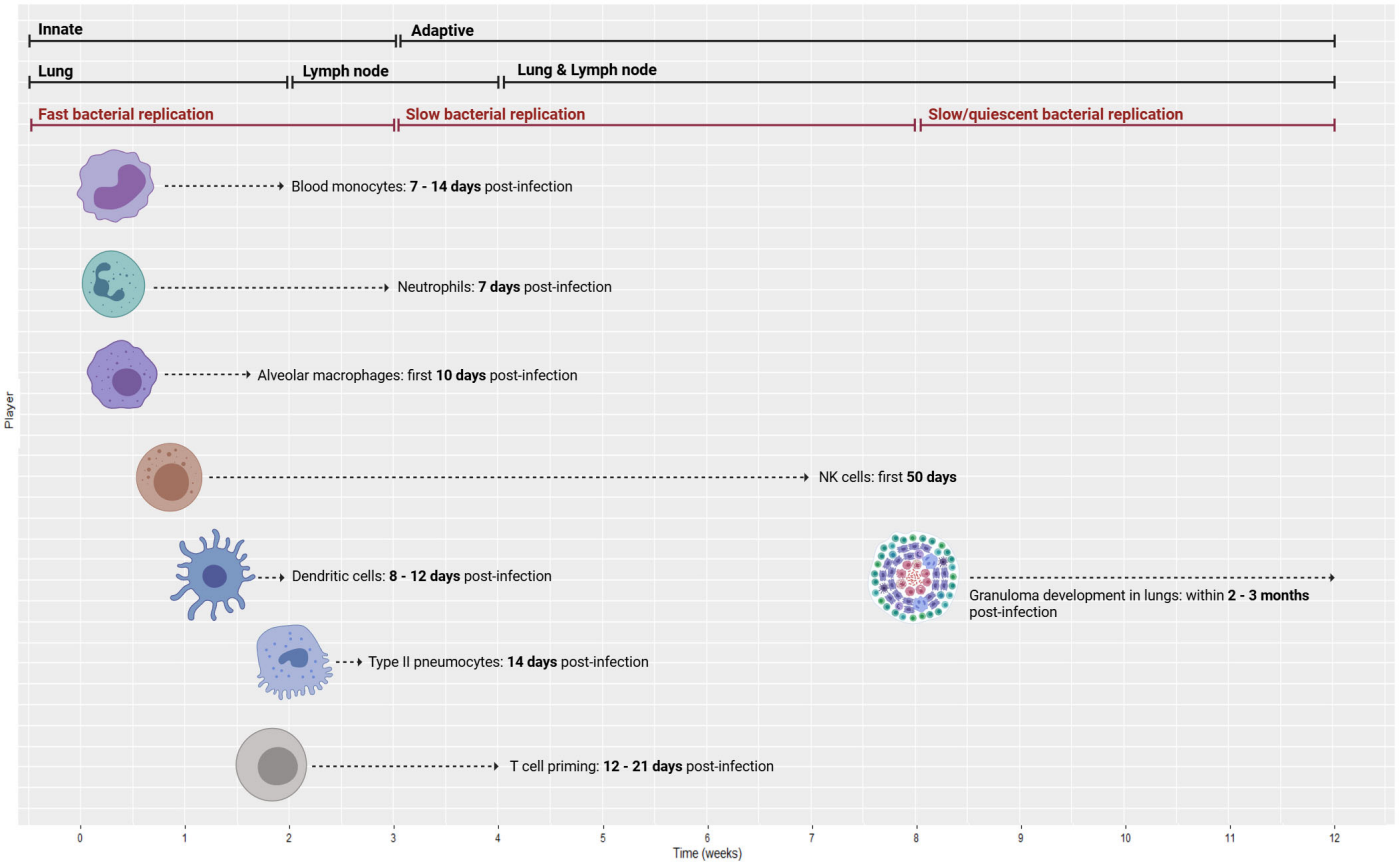
Time course of immune response from innate to adaptive (top bars) with the associated compartments (middle bars) and bacterial replication rates (lower bars). Temporal data are delineated for a 12-week period of the innate to adaptive response using animal models (mice and NHPs), ranging from blood monocyte recruitment to granuloma formation. NHPs, non-human primates. Created with BioRender.com.

**Figure 2** displays a 12-week period describing the transition from the innate to the adaptive response in mice and NHPs.

At 7–14 days post-infection in mice, blood monocyte recruitment increases significantly (30). At Day 14, type II pneumocytes release CXC chemokine ligand 5 (CXCL5) to recruit neutrophils to *Mtb*-infected lungs (30). Following *Mtb* infection, neutrophils are the first cells that infiltrate the lungs (31), with their numbers growing drastically 7 days post-infection (30). At Day 21, infected neutrophil numbers peak and then decline sharply thereafter (32).

Alveolar macrophages (AMs) are tissue-resident phagocytes that patrol the pulmonary surface for inhaled pathogens (33). In mice, *Mtb* predominantly remains inside AMs for the first 10 days after infection, directing the initial immune response and the shift of *Mtb* from the alveolar region to the lung interstitium (30). However, natural killer (NK) cells are crucial in the first 50 days of infection (34). NK cells produce cytokines, namely, IFN-γ, to enhance the immune response by spontaneously killing target cells without pre-stimulation (35).

In mice, an acquired immune response is delayed until 3–4 weeks post-infection because dendritic cells (DCs) deliver *Mtb* antigen to draining lymph nodes (DLNs) where the T-cell response is activated (36). In line with this delay in cell-mediated immunity, numerous murine models of TB have demonstrated that bacterial growth is high during the first 3 weeks of infection and then plateaus as adaptive immunity initiates (32). According to mouse studies, DCs move to the local lung DLNs (8–12 days post-infection) and drive naïve T-cell polarization (37). Then, T-cell priming begins 12–21 days post-infection (25). After 2–4 weeks, effector T cells travel back to the lungs through the blood (attracted by chemokines and pro-inflammatory cytokines that are released primarily by macrophages from the lung site of infection) to trigger an *Mtb*-specific immune response (38). By approximately 3 weeks post-infection, *Mtb* can be detected in monocytes and neutrophils in higher quantities compared to the initially infected AM (39). To provide a point of reference, available data on this process in humans suggest that the adaptive response is initiated at 6–8 weeks post-infection (39), while another paper indicates that there is a 2–3-week interval before host T-cell priming, which may benefit mycobacterial colonization (40).

As for granuloma formation in mice, *Mtb*-infected AMs migrate to the lung interstitium and form small aggregates at 2 weeks post-infection. These aggregates are believed to be the precursors to lung granulomas (39). Comparatively, NHP models have shown that *Mtb*-infected macaques develop 2–4-mm granulomas in their lungs within 2–3 months (41). Cynomolgus macaque experiments have shown that caseous granulomas form by 4–6 weeks, but non-necrotic granulomas appear later and mostly during active disease (see the “Granuloma” section for further details) (42). NHP studies have revealed that substantial bacterial death in granulomas occurs only after 10 weeks of infection (43). Further details on the immune mechanisms, cytokine release, and signaling activity are provided in the respective sections of each player.

## 5 Innate immune response

The innate immune response is the cornerstone of early response to *Mtb* invasion. Recent years have seen greater characterization of the innate response in TB due to a historic focus on adaptive immunity (22) that does not regard the 2–3-week delay in the onset of the adaptive response wherein bacterial replication occurs in humans (40). This is further exacerbated by the lack of vaccines that garner sufficient immune response. As such, this paper characterizes the time course of the innate response and its players to further the understanding of disease progression mechanisms. The key players of the innate response include epithelial cells [airway epithelial cells (AECs) and alveolar epithelial cells], neutrophils, NK cells, macrophages, and DCs. The innate response section is derived from human data.

### 5.1 Airway epithelial cells

#### 5.1.1 Background

The lungs are the major point of entry for respiratory pathogens due to their exposure to the outside environment during gas exchange. The airway contains the trachea, bronchi, and bronchioles; they are composed of ciliated cells, goblet cells, Clara cells, neuroendocrine cells, and regenerative basal cells (40). AECs are essential in the immune defense against *Mtb* in both the innate and adaptive arms (44).

#### 5.1.2 Recruitment

The very first immune player to encounter *Mtb* is the AEC following aerosol inhalation, thereby displaying its prominence in binding, recognizing, and internalizing *Mtb* to initiate an immune response within the airways (22). To reject the entry of pathogens, the host airway surface is covered by a range of resident (i.e., not recruited) tightly adhered epithelial cells. The cells form a mucosal barrier in the airways, which operates to both physically restrain and immunologically clear *Mtb* (40). Altogether, the human epithelium forms a physical barrier spanning 75 m^2^ (45).

#### 5.1.3 Recognition

Within the airways, AECs express various pattern recognition receptors (PRRs), such as toll-like receptors (TLRs), RIG-1-like receptors, NOD-like receptors (NLRs), and C-type lectin receptors (CLRs) which recognize Mtb cell wall components (22). This activates several signaling pathways that induce the production of cytokines [TNF-α, IFN-γ, granulocyte-macrophage colony-stimulating factor (GM-CSF), IL-6, and IL-10] and chemokines [IL-8, interferon gamma-induced protein-10 (IP-10), IL-27, monocyte chemoattractant protein-1 (MCP-1), and monokine induced by gamma interferon (MIG)] (22), all of which modulate the function of intraepithelial DCs (46). These cytokines also attract neutrophils and antigen-presenting DCs (47).

#### 5.1.4 Response

Upon sensing pathogens in the airways, AECs secrete antimicrobial effector molecules, enzymes, peptides, reactive oxygen species (ROS), reactive nitrogen species (RNS), and a range of mediators (cytokines, chemokines, and growth factors) (22). Specifically, the peptides cathelicidin (LL-37), β-defensin-2, and hepcidin are considered pivotal to the innate response in TB. Early secretion of these immune mediators elicits immune cell recruitment via communication with other cells to ultimately activate monocytes, phagocytes, lymphocytes, and polymorphonuclear leukocytes in the lungs (22). The activated innate response secondarily induces adaptive immune components (namely, recruitment and activation of DCs, T cells, and B cells that enhance antigen recognition and antibody production) (44).

**Figure 3:**
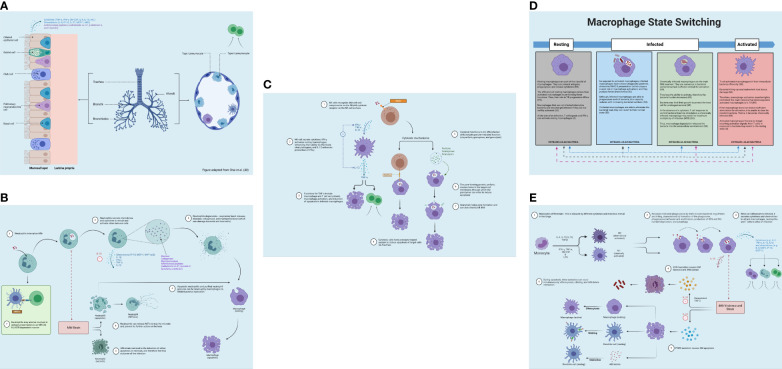
Innate immune response. **(A)** Airway epithelial cell immune response to *Mtb* invasion in the lungs (blue). Upon *Mtb* detection in the airways, airway epithelial cells secrete antimicrobial substances (e.g., peptides, enzymes, and cytokines). Pneumocytes produce bactericidal molecules. From Chai et al. (40). Reproduced with permission of SNCSC. **(B)** Neutrophil immune response to *Mtb* invasion in the lungs (blue) and lymph nodes (green). Neutrophils release antimicrobial enzymes and facilitate the apoptosis of infected macrophages. Neutrophils release NETs to ensnare the microbe and impede its further activity. **(C)** NK cell immune response to *Mtb* invasion in the lung (blue). NK cells promote immune stimulation through macrophage activation and using cytotoxic mechanisms. **(D)** Macrophage state switching in the lungs (blue). Macrophages can switch between the “resting” and “active” status depending on bacterial internalization via engulfment and degree of T-cell stimulation, which activates macrophages and optimizes bacterial killing. **(E)** Macrophage immune response to *Mtb* invasion in the lungs (blue). Macrophage defense entails activating antimicrobial effectors, initiating microbe intoxication mechanisms, limiting microbial access to nutrients, and generating antimicrobial peptides and cytokines, autophagy, and efferocytosis. NETs, neutrophil extracellular traps; NK, natural killer. Created with BioRender.com.

Concerning antigen presentation, AECs express major histocompatibility complex of class I (MHC I) molecules and can directly present intracellular antigens to resident CD8+ T cells in the lungs (22); essentially, they are “non-professional” antigen-presenting cells (APCs) (44). As myeloid DCs show a superior presenting ability (48), they are regarded as “professional” APCs (44). Within the lung epithelial cells, *Mtb* is localized in the late endosomal vacuole wherein antigens are presented to CD8+ T cells that subsequently stimulate IFN-γ production (22). Thus, AECs are important to initiating the adaptive response to *Mtb* (22) (**Figure 3A**). The roles of ciliated cells, Clara cells, goblet cells, neuroendocrine cells, and regenerative basal cells are provided in the **Supplementary Material**.

### 5.2 Alveolar epithelial cells

#### 5.2.1 Background

The alveolar epithelium also forms a major surface area that initially interacts with inhaled pathogens (45). The alveolar epithelial surface in the lung is mainly covered by type I (ATI) and type II (ATII) alveolar epithelial cells (pneumocytes), unlike the trachea, bronchi, and bronchioles (40) (**Figure 3A**). *Mtb* establishes infection of the lung in a myriad of ways, such as adhesion, invasion, and replication in AMs (see the “Macrophages” section) (44). The bacilli that successfully traverse the upper airways are delivered to the alveoli (49). However, it is yet to be explored how bacteria pass through the airway passage to enter the lungs (45).

#### 5.2.2 Recruitment

ATIs play a key role in gas exchange because they compose 90% of the alveolar wall and have a large, flat phenotype (40). ATII cells can generate a wide range of antimicrobial and pro-inflammatory molecules that support pulmonary immunity (44). Like AECs, pneumocytes are not recruited upon onset of infection; rather, they are already present in the lung compartment (44).

#### 5.2.3 Recognition

Alveolar epithelial cells express PRRs, including TLRs, NLRs, and CLRs, which are required to recognize microbe-associated molecular patterns (MAMPs) on *Mtb* (40).

#### 5.2.4 Response

Within the lungs, the first site of *Mtb* infection is the alveoli where it encounters lung epithelial cells and resident macrophages and DCs (50). Expressly, there is interplay between *Mtb* and resident phagocytes (macrophages and DCs) in the alveolar space (45). Macrophages and DCs are present in both the innate and adaptive arms. In the innate phase, macrophages respond to bacterial infections by phagocytosis, which involves engulfing the bacteria (50). Some of these macrophages are uninfected and unactivated (i.e., innate response that kills extracellular bacteria), which means that they do not contain bacteria (50). Activation is a prerequisite for efficient phagocytosis that is established in activated macrophages that do contain bacteria (i.e., adaptive response that kills intracellular bacteria), thus rendering uninfected/unactivated macrophages poor at phagocytosis (50). In the adaptive response, phagocytosis and bacterial eradication are heightened due to the presence of T cells and cytokines that enhance these capabilities (50). In addition, lung-resident DCs survey the lumen of alveoli and conducting airways to gather samples of antigens, which is also part of the innate response (40). DCs play further roles in bridging the two phases of immune response and in the adaptive response itself. Further details are provided in the “Macrophages” and “Dendritic cells” sections.

Specifically to alveolar epithelial cells, upon activation, they fortify the intracellular bactericidal effects of macrophages and increase the recruitment of lymphocytes and neutrophils to sites of infection (40). As a defense strategy, pneumocytes produce immunoglobulins, antimicrobial peptides, and surfactant proteins, which have bactericidal effects in TB. ATII cells release alveolar lavage fluid hydrolases that improve intracellular bacterial eradication by neutrophils (40). Human lung hydrolases also inhibit bacterial cell adhesion and therefore mycobacterial intracellular survival in human AMs by 60%–80% via mycobacterial cell envelope alteration (40).

Central to innate immunity, the ATII cells release CXCL5 to recruit neutrophils to *Mtb*-infected lung (30). At this early time point (Day 14 in mice), type I IFNs (whose role involves signaling in tissue-resident cells and hematopoietic cells) also elicit neutrophil infiltration in the airways (30). In addition, alveolar epithelial cells have an immunoregulatory role, elicited by tumor growth factor-beta (TGF-β) production: they are responsible for maintaining epithelial integrity and limiting inflammation to prevent tissue destruction (resulting from the inflammatory response) (22). Moreover, ATII cells can present antigens to T cells, which suggests that they may play a role in the adaptive immune response (44).

Ultimately, the alveolar epithelial cells directly partake in the innate immune within the lungs while, paradoxically, contributing to the dissemination of mycobacteria during primary infection by undergoing cellular necrosis (44). Necrosis is defined as an instant and uncontrolled form of cell death that results in lysis of the plasma membrane caused by extreme stress, releasing cellular contents to the extracellular space (51). That is, *Mtb* can invade and replicate in epithelial cells and macrophages within the lung alveolar spaces (44).

### 5.3 Neutrophils

#### 5.3.1 Background

Neutrophils are the first cells to partake in the process of lung infiltration in TB; they are also the most abundant cells in the lungs. The role of neutrophils in TB pathology is complex (31).

#### 5.3.2 Recruitment

After release from the bone marrow (BM), neutrophils circulate in the bloodstream until they receive inflammatory signals that trigger their migration into peripheral tissues (52). For example, airway epithelial cells release IL-8, GM-CSF, granulocyte-colony stimulating factor (G-CSF), ENA-78 (CXCL5), and Gro-α (CXCL1) (53); γδ T cells release IL-17 and IL-8 (53); macrophages and DCs release IL-8, GM-CSF, G-CSF, macrophage inflammatory protein-2 (MIP-2), LL-37, leukotriene B4 (LTB4), and Gro-α (53); T-helper cell type 17 (Th17) cells release IL-17 (53). Neutrophil recruitment is further enhanced by their release of mediators in response to mycobacteria (e.g., cytokines, leukotrienes, and granule products), which can further drive neutrophil recruitment to the lungs (53). Subsequently, the neutrophils become primed and activated as they migrate from the circulating blood; they then interact with *Mtb* and are stimulated by these cytokines and chemokines (53).

#### 5.3.3 Recognition

Neutrophil recognition of *Mtb* is characterized by pattern-associated molecular patterns (PAMPs) or damage-associated molecular patterns (DAMPs) via PRRs on the neutrophil, which heightens response and cellular recruitment to the site of infection (54).

#### 5.3.4 Response

After they arrive at the site of infection in the lung, neutrophils directly interact with and internalize mycobacteria (53). The interaction between mycobacteria and host cells is mediated via two mechanisms: opsonization and direct recognition (53). Opsonization involves removing antibody-coated antigens that are small enough for phagocytic engulfment (55).

Upon infection, neutrophils release IL-1 and chemokines (37) (e.g., MCP-1, IP-10, and MIP-1a/β) and pro-inflammatory cytokines (TNF-α and IFN-γ); this results in immune cell recruitment and activation (22). Similar to AMs, the *Mtb* strain can cause neutrophils to play varying roles in initiating cell death (necrosis or apoptosis), ultimately influencing the outcome of infection (37). Apoptosis is triggered by two pathways—intrinsic or extrinsic—depending on whether the bacterial source is intracellular or extracellular, respectively (51). Apoptosis involves cellular breakdown followed by the enclosing of the cytoplasmic contents within membrane-bound vesicles called apoptotic bodies (51). Necrosis, however, allows bacterial growth in host cells, thereby perpetuating the infection (51) (see “Alveolar epithelial cells” section for details on necrosis). Although efferocytosis (the removal of *Mtb*-infected apoptotic cells) is deemed advantageous for host defense, there is limited understanding of necrotic neutrophils infected with *Mtb* (56).

During *Mtb* infection, neutrophils generate and release antimicrobial enzymes (matrix metalloproteases, α-defensins, lipocalins, and lactoferrin) in the lungs (22), which are stored in granules (57). The purpose of this is to control bacterial growth in macrophages and aid apoptosis of infected macrophages. This limits pathogenic longevity in the host (22). However, neutrophils indiscriminately damage both bacterial and host cells during respiratory burst by releasing factors like elastase, collagenase, and myeloperoxidase (31). Additional innate immune cells and epithelial cells affected by *Mtb* also possess the potential to release enzymes that could result in the damage of pulmonary parenchyma, such as arginase, MMP-9, and gelatinase B (31). Moreover, the process of oxidative burst also releases ROS, which may propagate necrosis and therefore mycobacterial growth (58).

Research has shown that infected macrophages can uptake apoptotic neutrophils and purified neutrophil granules. As both contain active antimicrobial peptides, this process can inhibit bacterial replication (31). Neutrophil effector functions are regulated by the anti-inflammatory cytokine, IL-10, which reduces bacterial killing and modulates the secretion of pro-inflammatory cytokines and chemokines (37). In addition, neutrophils can produce pro-inflammatory cytokines, such as TNF-α, IL-12, IL-1β, and vascular endothelial growth factor (VEGF) (42), that enhance bacterial eradication in the adaptive response.

Neutrophils can also secrete neutrophil extracellular traps (NETs), which can ensnare the microbe and impede its further activity on the host (59). NETs are comprised of granule proteins and chromatin that are capable of binding to and eliminating extracellular pathogens (59). This process of NETosis, however, can also cause tissue injury by prompting unwanted immune activity (58). Beyond extracellular bacterial killing, NETs are also involved in the recruitment and activation of effector cells (51). Moreover, neutrophils may acquire the features of APCs via the expression of MHC II and co-stimulatory molecules, which occurs in the DLN (60); effectively, they can be involved in the cross-priming of CD8+ T cells (42). **Figure 3B** displays the role of neutrophils in the lungs.

### 5.4 Natural killer cells

#### 5.4.1 Background

NK cells are crucial for clearing intracellular pathogens in innate immunity (22). They form 15% of the lymphocytes found in the lungs and thus play a major role in infection outcomes (61). NK cells also produce cytokines to further enhance the immune response, namely, IFN-γ (35). NK cells can spontaneously kill target cells without pre-stimulation, although this capability is reinforced significantly by pre-stimulation via inflammatory signals or cytokines (35).

#### 5.4.2 Recruitment

The chemokines responsible for the recruitment of NK cells from the bloodstream to peripheral tissues include CXCL9, CXCL10, CC chemokine ligand 3 (CCL3), CCL4, and CCL5, which are produced by immune cells ranging from macrophages to mast cells (52).

#### 5.4.3 Recognition

NK cells function at an early stage of infection and are not limited by MHC restrictions (31). Several cell wall components of *Mtb*, including mycolic acids, serve as direct ligands for NKp44 (the natural cytotoxicity receptor found on NK cells) (31).

#### 5.4.4 Response

Cytotoxic cells can eliminate their targets through two primary pathways: death receptor-induced apoptosis and perforin/granzyme-mediated lysis (35, 51). Both of these mechanisms require close interaction between the cytotoxic cell and its target (35). NK cells can indirectly regulate bacterial growth by activating macrophages, thereby stimulating immune response and directly using cytotoxic mechanisms, which includes the secretion of cytoplasmic granules containing perforin, granzymes, and granulysin (31) [this protein is produced by human CD8+ T cells (62)]. Perforin (the pore-forming protein) creates openings in the target cell membrane to allow granzymes entry into the cell, which then initiate apoptosis (i.e., programmed cell death) (63), while granulysin may form pores and kill Mtb (62). The cytokines IFN-γ and IL-22, produced by NK cells, inhibit *Mtb* intracellular growth by enhancing phagolysosomal fusion (31). NK cell activity is regulated by cellular cytotoxicity and cytokine production (IFN-γ and TNF-α) (22).

Cytotoxic cells also induce apoptosis of target cells through a receptor-based system (35, 51). This involves tumor necrosis factor receptor (TNFR) superfamily expression on target cells (35). These receptors possess what is called the death domain, an intracellular signaling motif that attracts molecules like the Fas-associated death domain (FADD) to transmit the death signal (Fas-Fas ligand) (35). **Figure 3C** demonstrates NK cell activity in the lungs.

### 5.5 Macrophages

#### 5.5.1 Background

The lung macrophage population can be categorized into AMs and interstitial macrophages (IMs) (40). AMs form 90%– 95% of all immune cells in the alveolar space—they may develop from fetal monocytes (30). AMs are tissue-resident phagocytes that patrol the pulmonary surface for inhaled pathogens (33) and exhibit distinct phenotypic and functional characteristics that distinguish them from IMs (30). IMs are believed to have an immunoregulatory function in airway inflammation, whereas AMs are exceptionally efficient at recognizing and engulfing invading mycobacteria (40). AM movement from the alveolar lumen to the lung interstitium is driven by the ESAT-6 secretion system 1 and IL-1β signaling (64). Due to their predominant role in the immune response to *Mtb*, this review will focus on the role of AMs.

#### 5.5.2 Recruitment

First, the lungs must possess a baseline level of resting macrophages (resident macrophages) to continuously patrol the tissue for inhaled foreign invaders. Second, macrophages are recruited to the site of infection via mediator release (e.g., chemokines) by other macrophages, as shown in the “Response” subsection (4). Macrophages move to the site of infection through a combination of random motion and chemotaxis (i.e., directed cell movement) (50). This phenomenon is induced by bacteria-secreted chemokines to serve as signaling molecules that draw macrophages to the site of infection (50). During recruitment, the exit of monocytes from the BM is influenced by CXCR4 and C-C chemokine receptor type 2 (CCR2)-mediated signaling under homeostatic conditions (52).

#### 5.5.3 Recognition

AMs have a large capacity to identify and engulf invading mycobacteria by utilizing their abundant PRRs. Macrophages recognize *Mtb* surface MAMPs via PRRs and phagocytose them, but this process can be hindered by *Mtb* effector proteins to educe bacterial persistence (40).

#### 5.5.4 Response

##### 5.5.4.1 Phenotypes

Different microenvironmental signals drive macrophage differentiation and ultimately their development and function in TB. The M1 and M2 populations are two key phenotypes (31). Classically activated human M1 macrophages are induced by microbial stimuli (e.g., lipopolysaccharide) or cytokines (e.g., IFN-γ, TNF-α, and GM-CSF), and they produce stimulatory cytokines (31). This phenotype is effective in eradicating intracellular bacteria (31).

*Mtb* can incite AM polarization toward an alternatively activated M2-like phenotype, which allows for bacterial growth via anti-inflammatory cytokine production (IL-10 and TGF-β) (40). M2 macrophages are suppressors of Th1 responses (described in the “T cells” section) and are poor APCs (31). They are induced by IL-4 and IL-13 (Th2-type cytokines) (31, 65), IL-10, and TGF-β (31); as such, this macrophage population is thought to act in humoral immunity and tissue repair (65). Further, the deactivated M2 phenotype is thought to dampen immune response by producing anti-inflammatory cytokines and prostaglandin E (PGE) and reducing MHC II expression to limit antigen presentation (i.e., limit T-cell activation) in the adaptive response (65).

##### 5.5.4.2 Immune mechanisms

Upon reaching the lungs, mycobacteria are internalized through receptor-mediated phagocytosis by AMs (66) that patrol the airway surface (33); mycobacteria replicate herein, thereby creating infective foci in the alveolar walls (66). Phagocytosis can be divided into two processes: bacterial engulfment and killing. Initially, engulfment occurs because the macrophage receptors bind to the bacterial cell wall ligands. This encircles *Mtb* with the macrophage cell membrane and allows subsequent bacterial internalization to form a compartment (i.e., phagosome). The lysosomes within the macrophage (which contain antimicrobial enzymes, proteins, and peptides) can then fuse with the phagosome to produce a phagolysosome. The contents of the lysosome are subsequently released, resulting in bacterial death (50).

Macrophage defense entails the activation of harmful antimicrobial effectors, such as the production of ROS and nitric oxide (NO), the acidification or metal accumulation in the phagolysosome to intoxicate the microbes, the limitation of critical microbial nutrients (iron, fatty acids, or amino acids), and the generation of antimicrobial cytokines and peptides, as well as the induction of autophagy (33) [a homeostatic process that recycles cellular organelles into an energy conservation mechanism (51)] and efferocytosis to eradicate bacteria (33). Of note, mycobacterial metabolism relies heavily on specific carbon sources, such as pyruvate, acetate, or cholesterol. Hence, acidification of the phagolysosome leads to bacterial growth arrest (due to reduced mycobacterial metabolism) (33). Infected macrophages produce the enzyme inducible nitric oxide synthase (iNOS) and antimicrobial peptides that kill *Mtb* (40). The process of macro-autophagy can be employed by activated macrophages to dispose of senescent organelles and compartments, thereby eliminating intracellular bacteria (33). Additionally, the low pH level present in infected macrophages is vital for the optimal functioning of most late endosomal/lysosomal hydrolases (33).

To clarify the role of the mediators, evidence shows a cocktail of pro-inflammatory cytokines and chemokines are released in response to *Mtb* infection (37, 40), such as IL-12 (37), TNF-α, IL-1β, IL-6, IL-23, and GM-CSF, which are upregulated in infected human AMs (40). Infected macrophages also secrete several chemokines (e.g., IL-8, IP-10, MCP-1, and MIP-2) that attract immune cells (neutrophils, macrophages, and T cells) to infection sites (50). During *Mtb* infection, macrophages are also a major source of IL-10. This may aid in limiting host-induced tissue damage, as IL-10 inhibits chemokine production by immune cells, indirectly downregulating cell recruitment to infection sites (67). Notably, infected AMs experience a substantial alteration in their metabolic pathway, shifting toward glycolysis. This is characterized by increased levels of the pro-inflammatory cytokine, IL-1β, and reduced levels of the anti-inflammatory cytokine, IL-10. These cytokine levels stimulate intracellular bacterial killing, thereby contributing to early clearance of *Mtb* in humans (39).

The different roles of infected AMs determine the type of cell death that takes place downstream (apoptosis or necrosis), depending on the virulence and strain of *Mtb* (37). Lipoxin A4 (LXA4) promotes necrosis, whereas prostaglandin E2 (PGE2) is pro-apoptotic (37). Deregulated levels of TNF-α induce necrotic cell death, thereby causing bacterial dissemination (68). Necrosis is paramount to bacterial longevity and dissemination, as it promotes release into the extracellular milieu (51). Otherwise, the AM becomes apoptotic (37), which helps to restrain or eradicate the infection (68). Apoptosis is widely regarded as an anti-inflammatory process, which leads to the induction of TGF-β and PGE2 (i.e., anti-inflammatory mediators) while inhibiting the production of IL-6, IL-8, IL-12, and TNF-α (i.e., pro-inflammatory mediators) within the phagocytosing macrophages (53).

In the context of apoptosis, three distinct scenarios can occur concurrently (37). First, the apoptotic AM may interact with a resting macrophage in the lung, resulting in macrophage efferocytosis (engulfment). This causes the macrophages to transition from a “resting” state to an “active” state (37). The macrophage can switch between these states depending on the mycobacterial load (4, 50, 69) (**Figure 3D**). Second, the apoptotic AM may interact with a lung DC; the apoptotic AM can be captured by a DC, which then processes (via “nibbling”) and delivers the resultant fragments to antigen-specific T cells via MHC I (37). DCs that process *Mtb* antigen migrate to the local lung DLNs, initiating naïve T-cell polarization (37). DC movement to the DLNs is influenced by IL-12 release as well as additional chemokines, although IL-10 can prevent such movement (37). Third, *Mtb* debris extrusion from apoptotic AMs can engage with resting DCs, which is then processed and presented to antigen-specific T cells in the presence of MHC II or associated proteins (37). Then, an anti-inflammatory cytokine, IL-10, is responsible for downregulating activated macrophages (69). It suppresses MHC II expression and NO generation while offsetting IFN-γ-mediated antimycobacterial actions on macrophages (69). Research suggests that IFN-γ can control T-cell numbers by inducing apoptosis; this limits tissue damage by controlling macrophage activation (34). **Figure 3E** displays macrophage response in TB.

### 5.6 Dendritic cells

#### 5.6.1 Background

Sentinel cells (i.e., lung-resident DCs) scour the lumen alveoli and conduct airways for antigen samples (40). Lung-resident DCs co-ordinate with other immune cells in the lungs, such as AMs and AECs. This interaction aids in the maintenance of lung homeostasis, the regulation of inflammation, and the co-ordination of both innate and adaptive immune responses (46). DCs are paramount to bridging innate and adaptive immunity (70). They are the primary APCs that initiate adaptive immune responses through antigen presentation, co-stimulation, and the production of T helper-polarizing cytokines (71), rendering them the most efficient APCs (72).

#### 5.6.2 Recruitment

DCs are derived from two different sources depending on the environment: they are recruited from the BM at a steady state and from monocytes in the blood circulation in an inflammatory state (70). Immature or resting DCs (IDCs) are abundant at sites of *Mtb* infection (such as the lungs) during inflammatory responses. They are specialized for antigen uptake and subsequent processing, which is accomplished predominantly through receptor-mediated endocytosis (RME) (46). RME involves clathrin-producing membrane vesicles (membrane-associated proteins) that contain the microbes and internalize them (73).

#### 5.6.3 Recognition

Upon infection, *Mtb* enters the DC via the major receptor Dendritic Cell-Specific Intercellular adhesion molecule-3-Grabbing Non-integrin (DC-SIGN) by recognition of Mannose-capped lipoarabinomannan (ManLAM) on the *Mtb* cell surface (71). Ligation of DC-SIGN by *Mtb* ManLAM causes IL-10 release, a factor that has been linked to the suppression of DC maturation and the downregulation of co-stimulatory molecule production (71). DC maturation requires two specific signaling events for the maturation program to be fully activated (46). The first signal is derived from receptor-mediated antigen uptake, whereas the second signal (essential for DC maturation) is initiated by the recognition of molecules associated with the antigen, specifically PAMPs or DAMPs (46). PAMPs and DAMPs are detected by PRRs that are expressed on the surface of various subsets of DCs in the lungs (46).

#### 5.6.4 Response

Bacteria are also internalized and phagocytosed by DCs at the infection site; DCs are less susceptible to *Mtb* replication and are specialized in delivering bacteria to DLNs to trigger T-cell priming (i.e., the adaptive immune response) (74). After bacterial uptake, immature DCs undergo maturation, which diminishes their phagocytic and endocytic abilities. The maturation process also initiates the expression of immune-stimulatory molecules (75).

As DCs mature, they migrate through the afferent lymphatic vessels to the DLN (in the T-cell area) (72). This migration is influenced by IL-12 release (37) and chemokines (76). Specifically, CCL19 and CCL21 direct DCs into the deep paracortex of the DLN, where their co-localization enables T-cell scanning and the early start of an immune response (76). Homeostatic CCL19 and CCL21 production by fibroblastic reticular cells within the DLN promotes DC localization to the T-cell region (52). CXCL17 also influences DC (and macrophage) chemotaxis (52). Moreover, mature DCs (MDCs) produce and enhance expression of the chemokine CCL18, which attracts naïve T cells to DLNs as a specific operation (75). In summary, infected DCs migrate to the DLN to activate T cells, which then move back into the lungs to mount their effector functions (64).

IL-10 can block this movement (37). IL-10 is produced by numerous immune cells during infection (e.g., macrophages, T cells, and neutrophils) (67). During *Mtb* infection, macrophages are the primary source of IL-10; activated macrophage-derived IL-10 may operate to prevent host-induced tissue damage (67). IL-10 inhibits cytokine/chemokine production, precludes cellular apoptosis/necrosis, and alters the macrophage activation phenotype (67).

**Figure 4:**
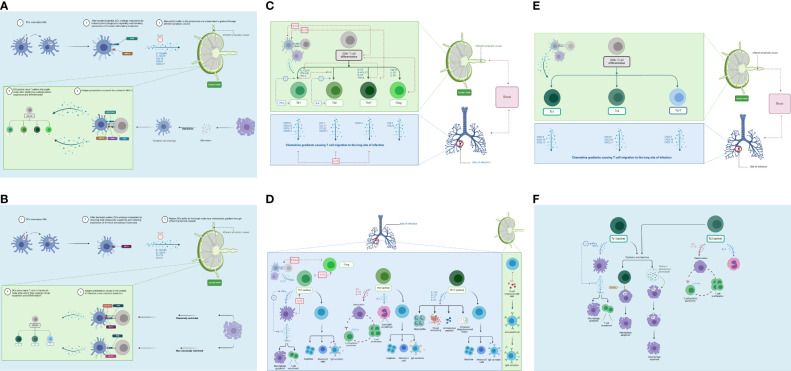
Adaptive immune response. **(A)** DC maturation and migration from the lungs (blue) and presentation to naïve CD4+ T cells in the lymph nodes (green). As maturation occurs, DCs migrate through the afferent lymphatic vessels and enter the T-cell area of the DLN. This migration is influenced by IL-12 release and chemokines. IL-10 can block this movement. DCs provide two primary actions within the DLN: naïve T-cell recruitment and antigen presentation. **(B)** DC maturation and migration from the lungs (blue) and presentation to naïve CD8+ T cells in the lymph nodes (green). CD8+ T cells can detect *Mtb* antigens (as peptides) presented by both classical and non-classical MHC molecules. **(C)** CD4+ T-cell differentiation in the lymph node (green) and migration to lungs (blue). Antigen-specific naïve CD4+ T cells undergo clonal expansion and effector differentiation. CD4+ T cells sense cytokines, which activate differentiation programs that result in their polarization toward specialized T-helper cell subsets. **(D)** CD4+ T-cell effector functions in the lungs (blue) and lymph nodes (green). Th1 cells produce IFN-γ to activate macrophages (eliminates intracellular *Mtb*). The Th2 response targets extracellular bacteria (i.e., humoral immunity). Th17 effector function is driven by IL-23; production of IL-17A, IL-17F, IL-21, and IL-22 to control extracellular bacteria. **(E)** CD8+ T-cell differentiation in the lymph node (green) and migration to the lung (blue). Following antigen presentation, CD8+ T cells can differentiate into Tc1, Tc2, and Tc17 cells. **(F)** CD8+ T-cell effector functions in the lungs (blue). Tc1 cells produce high levels of IFN-γ and TNF-α. Tc2 cells produce type II cytokines, which promote immune suppression. DC, dendritic cell; DLN, draining lymph node. Created with BioRender.com.

DCs provide two primary actions within the DLN: naïve T-cell recruitment and antigen presentation (72). Upon reaching the DLN, MDCs exhibit a mature phenotype that elicits high production of long-standing MHC I and MHC II molecules (from fivefold to 20-fold more than IDCs can produce). This allows for a more stable presentation of antigens. Following maturation, additional molecules that are expressed include co-stimulatory molecules like B7 (up to 100-fold), CD40, and Fas (75). In the DLN, the MDC population dynamics are governed by IDC maturation and migration from the lungs after phagocytosis (72). They are also subject to a natural death, which may explain the MDC “deactivation” (72). **Figure 4A** displays the process of DC maturation and presentation to CD4+ T cells.

Furthermore, CD8+ T lymphocytes can detect *Mtb* antigens as peptides, presented by both classical and non-classical MHC molecules (62). Classically restricted CD8+ T cells recognize antigens presented via classical MHC Ia (HLA-A, HLA-B, and HLA-C) molecules (62). Non-classically restricted CD8+ T cells recognize antigens via HLA-E molecules (non-MHC Ia), glycolipids linked with group 1 CD1 molecules and MR1 molecules, such as mucosal-associated invariant T (MAIT) cells (62). **Figure 4B** shows the process of DC maturation and presentation to CD8+ T cells.

## 6 Adaptive immune response

The adaptive immune response generates in the DLN and requires lymphocyte–DC (antigen-bearing cells) interactions. This process requires the co-ordinated migration of immune cells (innate and adaptive players) and is regulated by mediators (chemotactic cytokines and chemokines) (76). The adaptive response section is derived from human data.

### 6.1 T cells

#### 6.1.1 Background

Naïve T cells recirculate repeatedly through the bloodstream and the DLN, with a constant number arriving and a variable number leaving, depending on the degree of successful antigen presentations by MDCs (72). Although CD4+ and CD8+ T cells defend against *Mtb*, T-cell priming and differentiation must occur before T-cell effector functions can be enforced (72). To elicit T-cell response, secondary lymphoid organs (SLOs) must contain numerous mature T cells (77). Upon thymic export, T cells acquire a quiescent state before antigen presentation takes place, rendering them “naïve” cells (35, 77). In TB, T cells have two key roles: cytokine production to regulate the cell-mediated immune response and apoptosis-mediated elimination of infected macrophages (69). Bacterial eradication is reliant on CD4+ T cells to prevent active disease due to their production of IFN-γ, while CD8+ T cells are predominant in TBI (78). Of note, CD4+ T cells have been the primary focus of TB research over the last few decades owing to their significance in infection control (79). However, research suggests that CD8+ T cells demonstrate comparatively faster responses than CD4+ T cells (80). This could be ascribed to evidence that shows how CD8+ T cells become fully activated within 24 hours of antigen stimulation, which is a relatively short time (81).

#### 6.1.2 CD4+ T cells

##### 6.1.2.1 CD4+ T-cell differentiation

In the DLN, DCs present antigens to naïve CD4+ T cells (see “Dendritic cells” section) (72). Naïve CD4+ T cells (Th0) are primed in SLOs following binding to MHC II and co-stimulatory molecules (82). This is elicited by professional APCs, which have encountered antigens in SLOs or the periphery (82). Subsequently, antigen-specific naïve CD4+ T cells undergo clonal expansion and effector differentiation (82). During these processes, CD4+ T cells are exposed to a cytokine-rich environment, produced by numerous cells including infected cells, DCs, and stromal cells (82). This results in the activation of the CD4+ T-cell differentiation programs, thus causing their polarization toward specialized T-helper cell subsets (82). **Figure 4C** displays the process of CD4+ T-cell differentiation and migration to the lungs.

Th1 cells develop from the presence of IL-12 (secreted by macrophages and DCs following phagocytosis of bacterial pathogens) (66), IFN-γ (72), and type I interferons (comprising different isoforms of IFN-α) (82). Type I interferons are intrinsic molecules released by diverse cells in response to infection (82), such as infected macrophages (22). A Th1 response is invoked when naïve CD4+ T cells are primed in the presence of both IL-12p70 and IL-23 (produced by DCs and macrophages). This is because IL-12p70 inhibits Th17 differentiation (83) (i.e., Th17 inhibits Th1 response). IL-12(p40)_2_—homodimeric IL-12p40—also acts as a chemoattractant for macrophages; it also induces TNF-α and NOS (84). Type I interferons increase DC maturation, which makes antigen presentation and co-stimulation to naïve T cells more efficient (82). Finally, type I IFNs sensed by CD4+ T cells protect them from NK cell-mediated killing (82). Moreover, IFN-γ heightens the rate of Th0 to Th1 differentiation by combating IL-4-mediated opposition to this process (69). However, an excessive and persistent Th1 response may result in inflammation and tissue damage (72).

Further, IL-4 drives Th0 to Th2 differentiation (69). It is mainly produced by Th precursor and Th2 cells (72). IL-4 is responsible for regulating the pro-inflammatory Th1 response by downregulating Th0 to Th1 differentiation; this inhibits Th1 formation (69). IL-4 also upregulates Th2 differentiation as it encounters antigens in a positive feedback loop (77).

The differentiation of human Th17 cells from naïve CD4+ T cells is driven by IL-1β and IL-6 (produced by DCs and macrophages). However, the effector function of memory CD4+ Th17 cells requires IL-1β and IL-23 (83). Th17 cell differentiation can be initiated by TGF-β (produced by DCs and macrophages) in the presence of IL-6 or IL-21 and later IL-23 (pro-inflammatory cytokines) (85). DCs produce IL-27, which is thought to display the greatest ability in inhibiting Th17 cell differentiation and autoimmune inflammation (85). DC-derived IL-10 also prevents Th17 cell differentiation via the constraint of DC IL-1 production (85).

In the absence of pro-inflammatory stimuli, the anti-inflammatory cytokine, TGF-β, causes induced Foxp3+ regulatory T (iTreg) cell differentiation from naïve CD4+ T cells (85). The cytokines IL-10, TGF-β, and IL-35 stimulate adaptive Treg cell differentiation (86). IL-35 (a member of the IL-12 cytokine family) is thought to be strictly immunosuppressive; it is produced mainly by DCs and regulatory T cells (87). IL-35 inhibits T-cell proliferation (87). Type I IFNs also have a role in a feedback loop: they weaken Treg immunosuppression activities to reinforce CD4+ T-cell activation (82).

##### 6.1.2.2 CD4+ T-cell migration to the lungs

Naïve CD4+ T cells change functionally and phenotypically, enabling primed T cells to proliferate and migrate to the site of infection via the efferent lymphatic vessel and blood (72). The cytokine environment governs the CD4+ T-cell commitment to become one of various effector T cells both during migration and at the site of infection (72).

After exposure to *Mtb*, antigen-specific Th1 cells migrate back to the lungs under a chemokine gradient (37). The cocktail of chemokines is produced by mast cells, the cytokine-activated epithelium, DCs, and macrophages among a host of other resident innate immune cells. The functional CD4+ T cells migrate to the lungs via CXCL9, CXCL10, CXCL11, and CX3CL1 for Th1 cells; CCL1, CCL17, CCL22, and CX3CL1 for Th2 cells; CX3CL1 for Th17 cells; and CCL1 and CCL22 for iTreg cells (52). IL-10 blocks this chemotactic movement for Th1, Th2, and Th17 cells (37).

##### 6.1.2.3 CD4+ T-cell effector functions

**Table 2** provides the details on CD4+ T-cell effector functions (**Figure 4D**).

**Table 2:** T-cell effector functions by subset.

CD4+ T cell vs. CD8+ T-cell effector functions	
CD4+ T cell subset	
**Th1 cells**	In the lungs, Th1-type cytokines (IFN-γ, TNF-α, and IL-12) are central to protective immunity, as they activate macrophages to further antimicrobial activity (79). IFN-γ is produced mainly by Th1 cells; this occurs before and after activated macrophage response (72). IFN-γ activates macrophages, which is critical for intracellular bacterial elimination (a distinctive feature of *Mtb*) (66). Macrophages are the main target cells; however, following macrophage activation, they can kill intracellular bacteria and heighten the protective Th1 response (72) (**Figure 4D**). Activated and resident macrophages kill extracellular bacteria, while intracellular bacteria are only killed when the infected macrophage dies via cytolytic action or apoptosis (72). Infected macrophages secrete cytokines like TNF-α to recruit CD4+ and CD8+ T cells and activate their effector functions at the infection site (121). TNF-α induces apoptosis of infected macrophages (121); apoptosis can also occur by the Fas-FasL pathway (72). FasL is a membrane-bound molecule that is expressed by CD8+ and Th1 cells (63). The binding of the FasL to its receptor induces death by apoptosis in target cells (63). Following the apoptotic death of infected macrophages, neutrophils are attracted to the sites of mycobacterial release within the extracellular space (66). IL-12 is produced by macrophages in response to antigen stimulation. Its main function is to induce differentiation of Th0 lymphocytes into Th1 lymphocytes and enhance the production of IFN-γ (69). When macrophages are primed with IFN-γ, the production of IL-12 is greatly increased; however, the production can also be inhibited by IL-10 (69). Both Th1 and Th2 cells also produce IL-10 (72). IL-10 and other cytokines deactivate macrophages (72). IL-10 inhibits the production of cytokines and chemokines; this prevents cellular apoptosis and necrosis and alters the activation phenotype of macrophages (67). It dampens MHC II expression and NO production while circumventing the antimycobacterial effects of IFN-γ on macrophages (69).
**Th2 cells**	The Th2 response targets extracellular bacteria (i.e., humoral immunity) (72). Mature Th2 cells produce IL-4, IL-5, and IL-13 (77) and IL-10 (72). IL-4 promotes further Th2 cell differentiation in naïve T cells as they encounter antigens in a positive feedback loop (77). IL-4 and IL-13 reduce macrophage bacterial killing by dampening cellular responsiveness to IFN-γ and inhibiting iNOS production (65). IL-4 also mediates IgE class switching in B cells and IL-5 recruits eosinophils (77), which express the chemokine receptor CCR3—this enables eosinophils to respond to various inflammatory stimuli (52) (**Figure 4D**).
**Th17 cells**	Th17 effector cell function is governed by IL-23, which is characterized by the production of IL-17A, IL-17F, IL-21, and IL-22 (77). Th17 cells are thought to play an important role in controlling extracellular bacteria (77). The pro-inflammatory responses of IL-17A, IL-17F, and IL-22 include neutrophilia, tissue remodeling, and antimicrobial protein production (85). Meanwhile, IL-22 enhances phagolysosomal fusion, which inhibits *Mtb* intracellular growth (31) (**Figure 4D**). For further information on the roles of iTreg cells and B cells, see the **Supplementary Material**.
CD8+ T cell subset	
**Tc1 cells**	Functionally, Tc1 cells are characterized by their high levels of IFN-γ and TNF-α (88, 122). Resting macrophages are activated by IFN-γ, which enhances their pathogen-clearing ability and cytokine release (69). Cytotoxic CD8+ T cells also produce IFN-γ, which induces MHC class I expression to increase the chances for recognition and bacterial killing; IFN-γ activates macrophages for further bactericidal activity (i.e., phagocytic functions and antigen presentation) (63). IL-12 enhances the production of IFN-γ (69). T cells are killed by IFN-γ-induced apoptosis and natural cell death (based on their half-life) (34). Functions of TNF-α include recruitment of macrophages and T cells, activation of macrophages (with IFN-γ and bacterial signals), and induction of apoptosis of infected macrophages (34) (**Figure 4F**). Further, Tc1 cells produce high levels of perforin and granzyme B, thus demonstrating exceptional cytotoxic activity (88, 122). This bacterial killing process, cytolysis, involves the use of granules containing perforin, granzymes, and granulysin to destroy the infected cells (62). Similar to NK cells, the pore-forming protein, perforin, penetrates the target cell membrane, allowing granzyme entry to induce apoptosis (63). Granulysin has antimicrobial activity and is pro-apoptotic (63)(**Figure 4F**).
**Tc2 cells**	Tc2 cells are known for their production of type II cytokines, such as IL-4, IL-5, and IL-13 (88, 122). IL-4, and IL-13 promote immune suppression (65, 122). IL-4 and IL-13 reduce iNOS synthesis and cellular responsiveness to IFN-γ (65). IL-5 recruits eosinophils (77). These type II cytokines promote B-cell class switching for IgE production (77). Tc2 cells also express high levels of granzyme B, possessing cytotoxic abilities similar to Tc1 cells (88) (**Figure 4F**). Following bacterial elimination, the T-cell response is concluded via antigen removal, thereby restricting T-cell activation and abrogating the recruitment of new effector T cells (77).
**Tc9, Tc17, Tc22, and CD8 memory T cells**	The effector functions of Tc9, Tc17, Tc22, and CD8 memory T cells can be found in the **Supplementary Material**.

#### 6.1.3 CD8+ T cells

##### 6.1.3.1 CD8+ T-cell differentiation

Following antigen presentation, CD8+ T cells can differentiate into Tc1, Tc2, and Tc17 cells. The cytokine milieu in human TB remains unclear. However, research in related areas (respiratory diseases, tumors) has provided insights, some which are included here for context. Induction of Tc1s is mediated by IL-12, which is produced mostly by APCs (i.e., macrophages and DCs) in response to pathogen-derived maturation stimuli (88). The polarization of Tc2 cells is driven by IL-4; this activates the transcription factors STAT6 and GATA3 to induce the expression of genes that propagate Tc2 formation (88). The polarization of Tc9 cells requires the combined actions of IL-4 and TGF-β (88). The differentiation of Tc17 cells involves the combination of IL-6 and TGF-β, which is further enhanced by IL-1β, IL-21, and/or IL-23 (88). Further, IL-6 in conjunction with TNF-α has been shown to govern Tc22 differentiation (88). The presence of Tc9 and Tc22 cells in human TB is undefined and has yet to be examined.

##### 6.1.3.2 CD8+ T-cell migration to the lungs

Infected AMs secrete cytokines (IL-1, TNF-α, IL-12, and IL-6) (37) and chemokines (e.g., IL-8, IP-10, MIP2, and MCP-1) to attract neutrophils, macrophages, and T cells to infection sites (50). CD8+ T cells migrate to the lungs under a chemotactic gradient involving CXCL9, CXCL10, and CXCL11 for all CD8+ T cell subsets (52) to mount an *Mtb*-specific immune response. **Figure 4E** displays the process of CD8+ T-cell differentiation and migration to the lungs.

##### 6.1.3.3 CD8+ T-cell effector functions

**Table 2** provides details on CD8+ T-cell effector functions (**Figure 4F**).

## 7 Granuloma

### 7.1 Background

In TB, the hallmark of the host–microbe interaction occurs at the level of the granuloma (38)—an organized aggregate of immune cells (89) that immunologically constrain and physically contain the bacteria, albeit frequently failing to eradicate it (41). In human disease, TB manifests as distinct types of lesions, such as anaerobic caseous necrotic lesions and aerobic cavities (90), all of which can exist in a single infected patient (91). That is, upon infection, several granulomas can grow within a single lung. Notably, however, granulomas develop independently of one another and have largely heterogeneous functional trajectories, thus further complicating treatment (41). Specifically, NHP and human granulomas are heterogeneous in their histopathology, inflammatory signaling activity, metabolic activity, and bacterial load (29). This results partly from the heterogeneous nature of granuloma formation (91). In addition, granulomas have a double-edged nature: on the one hand, they serve to control *Mtb* infection; on the other, they provide a survival niche for the bacteria (92).

For the “Granuloma” section, a systems biology approach was taken—human data were used for consistency, and animal data were used (mice and NHPs) to complement the narrative, such that a fuller representation of granuloma response in TB is afforded. The human versus animal data are specified to make a clear distinction.

### 7.2 Formation

#### 7.2.1 Mediators and signaling

In humans, the primary granulomatous interactions occur in the peripheral part of the lung close to the pleura (93). Later, granulomas can also form in the DLNs (74). The formation of granulomas in the early stages relies on the presence of persistent stimuli (94) resulting from the failure of the immune system to clear *Mtb* (74). Namely, sustained TNF-α signaling by infected macrophages and T cells maintain chemokine concentrations that enable cellular recruitment and retention of granuloma structures (94).

NHP studies have shown that IFN-γ can also induce the production of chemokines (e.g., CXCL9, CXCL10, and CXCL11) (62). This is thought to enable the migration of cells to form the granuloma (62). A balance between TNF-α and IL-10 concentrations is crucial for controlling infection within a single granuloma (92). In addition, molecular mediators also characterize granuloma dynamics (e.g., IFN-γ, TNF-α, IL-10, CXCL9, CXCL10, CXCL11, CCL2, and CCL5) (38).

#### 7.2.2 Four phases of development

**Figure 5:**
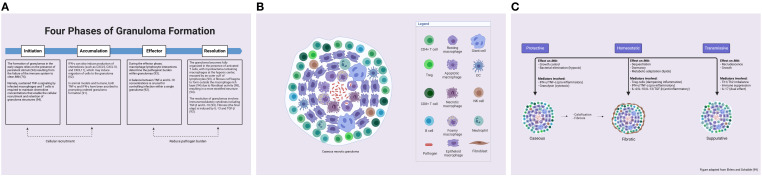
Granuloma formation and response. **(A)** The phases of granuloma formation (lungs and lymph nodes). Granuloma formation can be separated into four distinct phases: initiation, accumulation, effector, and resolution. **(B)** A fully formed tuberculous granuloma consists of a central zone of caseating necrosis, surrounded by neutrophils, NK cells, T cells, B cells, DCs, epithelial cells, and fibroblasts. The primary cellular component of the structure is the macrophage, which develops new phenotypes. **(C)** TB granulomas manifest as diverse types (caseous, cellular necrotic, and fibrotic). Classic caseous necrotic granulomas feature a caseous necrotic center (dead immune cells and lung tissue), which is surrounded by layers of macrophages and then a smaller cuff of lymphocytes. The non-necrotizing granuloma consists mainly of macrophages with limited lymphocytes. Granuloma fibrosis and/or calcification occur naturally during infection, forming a fibrotic cuff to aid granuloma containment and restrict bacterial dissemination. Suppurative granulomas are heavily infiltrated by neutrophils and can cause bacterial transmission. DCs, dendritic cells; TB, tuberculosis. From: Ehlers, S. & Schaible, U.E. The granuloma in TB: dynamics of a host-pathogen collusion. *Front Immunol* 3, 411 (2012). Created with BioRender.com.

In humans, granuloma formation may be divided into four phases: initiation, accumulation, effector, and resolution (93) (**Figure 5A**). Both the initiation and accumulation phases involve cellular recruitment (93). In knockout mouse experiments, both TNF-α and lymphotoxin-α have proven integral to granuloma formation (93). They regulate chemokine receptor and adhesion molecule expression, and they establish chemokine gradients (93). In animal models and humans, both TNF-α and IFN-γ have been ascribed to prompting ordered granuloma formation (91). During the effector phase, macrophage–lymphocyte interactions determine the pathogenic burden within granulomas (93). Finally, the resolution phase involves immunomodulatory cytokines including TGF-β and IL-10 (93). Fibrosis—the final step in granuloma resolution—is induced by IL-13 and TGF-β (93).

#### 7.2.3 Cellular recruitment and function

In humans, NHPs, and mice, the cells involved in granuloma structures include neutrophils, NK cells, T cells, B cells, DCs, epithelial cells, and fibroblasts (32) (**Figure 5B**). However, the macrophage is the primary cellular component responsible for granuloma formation and is the major cell type found in most granulomas (25). Paradoxically, macrophages house the majority of *Mtb* but also have among the most efficient effector functions to kill the bacteria (25). In humans, it is the M1 macrophage phenotype that predominates granuloma formation and inflammatory response (40).

In humans, NHPs, and mice, new macrophage phenotypes such as epithelioid macrophages, multinucleated giant cells, and foamy macrophages (FMs) appear within the granuloma during the phase of containment and cellular consolidation (36, 91). The array of macrophage phenotypes has different functions, for example, antimycobacterial effector mechanisms, cytokine production, and tissue remodeling (due to the secretion of chemokines and proteins) (25). Specifically, epithelioid macrophages have firmly interlaced cell membranes linking adjacent cells—this is thought to optimize the pathogen-containing properties of granulomas (32). Multinucleated giant cells result from the fusion of macrophages (32); they have multiple nuclei arranged in a semi-circle, and their function (while not well understood) is thought to involve bacterial control and inflammation (28). Finally, in humans specifically, *Mtb*-infected macrophages induce the formation of FMs by the accumulation of lipid bodies (LBs) (95). Said accumulation results from an imbalance between the serum influx and efflux of low-density lipoprotein particles (94). Within the FM, *Mtb*-containing phagosomes gradually surround and engulf the LBs—nutrients for the mycobacteria (95). The release of *Mtb* cell wall lipids into the extracellular space (by macrophages) has been shown to generate further LB accumulation in uninfected macrophages, resulting in the growth of human TB granulomas (96). Although FMs are central to granuloma development and maintenance, they also demonstrate lower phagocytic capabilities (96). Nonetheless, macrophages contribute to the majority of infection control mechanisms and inflammatory responses within the granuloma (25).

In humans, NHPs, and mice, T cells form a key structural and functional component of granulomatous lesions due to their roles in the containment and progression of *Mtb* infection (26). For optimal function, T cells must be activated by infected macrophages—this, in turn, causes cytokine release to activate macrophages or kill infected cells via cytotoxic mechanisms (62). The outer cuff of lymphocytes also engages in antigen presentation with APCs to trigger a response at the site of infection (91). It is noteworthy that T cells are involved in all four phases of granuloma development in humans (93) (**Figure 5A**).

In mice, neutrophil-mediated clearance of infected macrophages inside lesions operates via two pathways: first, by directly reducing bacterial loads and, second, by reducing intercellular bacterial dissemination into uninfected macrophages and the proceeding cell death (89). In addition, NK cells are present in mature granulomas in the lungs of *Mtb*-infected patients (31).

In humans, the role of epithelial cells in granuloma pathogenesis is juxtaposed (32). On the one hand, epithelial cell-mediated MMP-9 production heightens macrophage recruitment (32, 97) (a deleterious effect due to the resulting mycobacterial growth) (97); on the other hand, their IFN-γ-mediated signaling inhibits neutrophil recruitment (a protective mechanism via inflammation control) (32). The role of B cells is vague, although studies have shown that they contribute to infection control in TB (28). NHP studies have shown that fibroblasts contribute to fibrosis by inducing collagen deposition (98).

#### 7.2.4 Fibrosis and calcification

The granuloma becomes fully organized in the presence of activated T cells, with mycobacteria-containing macrophages at the hypoxic center encased by an outer cuff of lymphocytes (99). A fibrous cuff begins to form outside the macrophage-rich layer (94) due to fibroblast activity (98), resulting in a more stratified structure (94). Subsequently, most of the lymphocytes start to aggregate outside the fibrous cuff (94). Upon cellular recruitment to the site of infection, the granuloma structure becomes highly vascularized; this is due to a VEGF-mediated response (94). VEGF enhances angiogenesis, which promotes bacterial growth by relieving hypoxia (100). The high level of stratification in human and NHP granulomas suggests that protection depends on microenvironments that boost bacterial clearance while averting damage to neighboring host tissue (42).

In humans, a mature (i.e., fully formed) granuloma comprises a caseous necrotic core, surrounded by activated macrophages, DCs, T cells, B cells, and fibroblasts (93). These bacterial-containment granulomas often do not develop into active sites and have been shown to resolve (94). Fibro-calcified granulomas demonstrate enhanced infection control and often have lower bacterial burdens due to their ability to physically impede *Mtb* (98) (**Figure 5C**).

### 7.3 Granuloma types

In humans and NHPs, TB granulomas manifest as different types (caseous, cellular necrotic, fibrotic, and suppurative) (74). The details of granuloma types in TB, their cellular composition, and function are provided in **Table 3**.

**Table 3:** Granuloma types, cellular compositions, and functions.

Granuloma type	Cellular composition	Function
**Caseous necrotic**	NHP studies have shown that the classic caseous necrotic granulomas result from the necrotic death of participating cells (32, 91); they feature a caseous necrotic center formed of dead immune cells and lung tissue, which encase bacteria that cannot grow due to the hypoxic conditions (38). The caseous center is surrounded by layers of macrophages that are enclosed by a cuff of lymphocytes (38) (**Figure 5C**). In humans, the necrotic caseous granuloma is the most commonly formed in active TB but is also formed in TBI (42). The human caseous granuloma (so named due to its cheese-like appearance) consists of an acellular necrotic center surrounded by epithelioid macrophages and neutrophils, and a lymphocytic cuff (which contains both B and T cells) (25). Most importantly, the death of FMs results in the accumulation of lipid debris that form the caseum (99). The lipid droplets elicit eicosanoid production, which aids in host defense and skews macrophage death toward apoptosis (i.e., more protective as opposed to necrosis) (51).	This granuloma type sequesters the bacteria from the surrounding tissue and lowers the bacillary replication rate (2). In humans, despite the protective role of this granuloma type, some can show increasing lipid accumulation in the caseum (94). This induces necrosis and subsequent collapse of the granuloma core, thereby releasing bacilli into the airways (94). Further, necrotizing granulomas are formed upon failure to eradicate *Mtb* or to modulate inflammation (29).
**Cellular non-necrotizing**	In humans, the non-necrotizing granuloma consists mainly of macrophages with limited lymphocytes (25).	Cellular non-necrotizing granulomas play a role in maintaining immune balance via *Mtb* sequestration, dormancy, and metabolic adaptation (99) (**Figure 5C**).
**Fibrotic**	In NHPs and humans, granuloma fibrosis and/or calcification occur naturally during infection (124). The process of fibrosis involves fibroblasts, which proliferate in wounds where they differentiate into myofibroblasts and secrete extracellular matrix proteins such as collagen (125). In NHPs, peripheral fibrosis involves a collagen cuff that surrounds the perimeter of granulomas (**Figure 5C**). This fibrotic cuff is thought to contribute to granuloma containment and restrict disease dissemination (125). In NHPs, the other type of fibrotic granuloma morphology involves a collagenous structure throughout (referred to as “centrally fibrotic”). These granulomas appear as scars and are thought to be involved with the sterilization of a granuloma (i.e., clearing all the bacteria inside). Centrally localized fibrosis is more common following antibiotic treatment (125). In human chronic or TB infection, caseous granulomas can also become calcified, which commences at the caseous center (25). The calcium salts are typically deposited diffusely throughout the necrosis, but they may be deposited concentrically on occasion (93) (**Figure 5C**).	A calcified granuloma typically indicates a successful immune response and houses fewer inflammatory cells than other granulomas (25).
**Suppurative (necrotic neutrophilic)**	In humans and NHPs, suppurative (or necrotic neutrophilic) (123) granulomas are heavily infiltrated by neutrophils (42). IL-17 has a dual effect: it benefits bacterial killing, mediated through the recruitment of CD4+ effector T cells (which produce IFN-γ), but is damaging in excess due to increased neutrophil recruitment (32). That is, neutrophilic cytotoxic molecules may cause notable tissue destruction and remodeling (99) (**Figure 5C**).	This granuloma type participates in bacterial transmission. It can prompt active lesion development, providing further host cell lipid substrate for *Mtb* growth and biofilm formation to cause transmission (99).
**Leukocyte aggregates**	Beyond the granuloma types discussed in this paper, recent research has shown that human TB lungs also contain non-necrotizing leukocyte aggregates that are spatially organized to surround necrotizing granulomas (29).	It is thought that these non-necrotizing lesions impact the outcome of immune response by co-ordinating with neighboring necrotizing granulomas (29).

TB, tuberculosis; FM, foamy macrophage; NHPs, non-human primates.

## 8 Bacteria

### 8.1 Background

**Figure 6:**
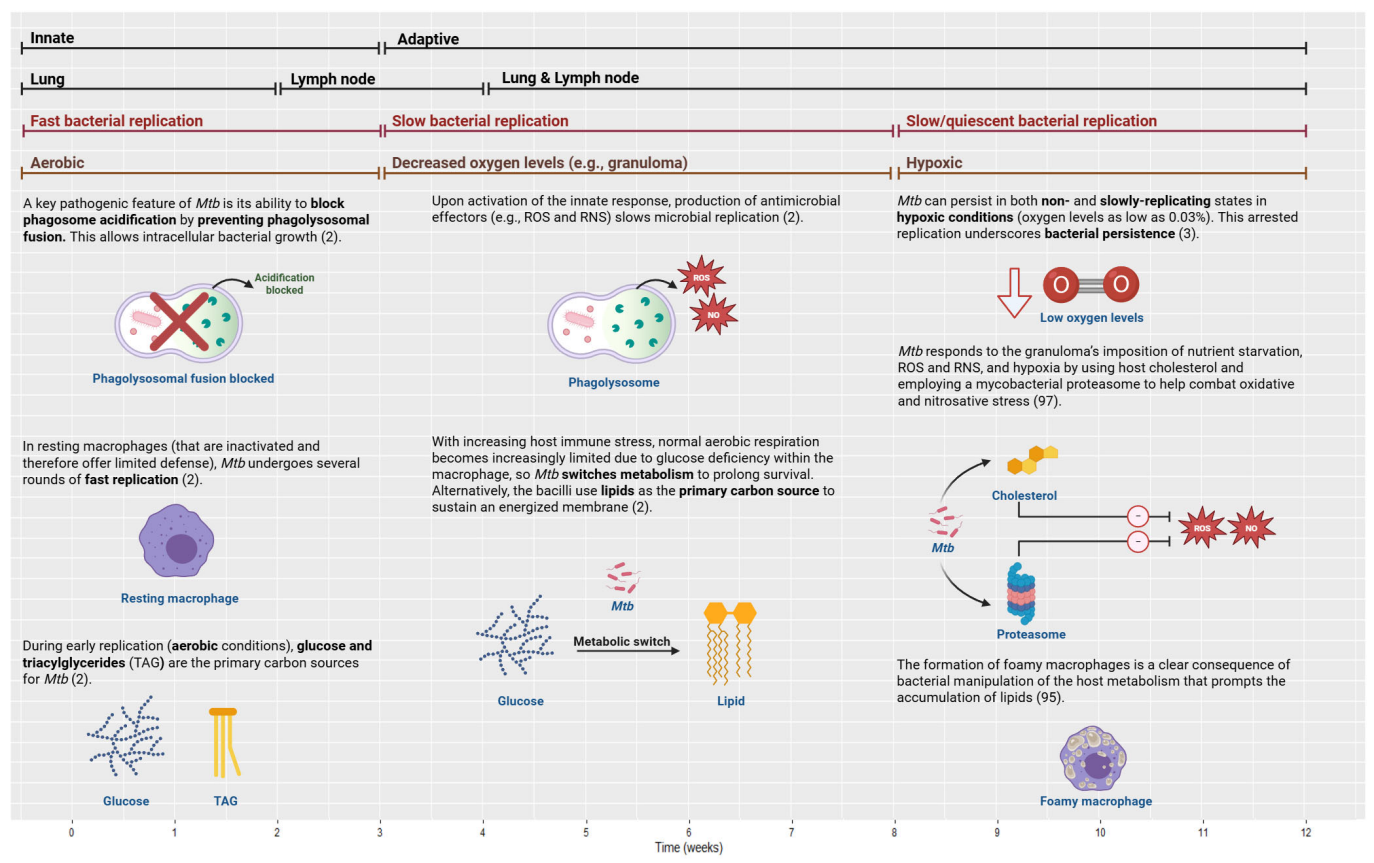
Bacterial growth and manipulation in TB over time from innate to adaptive (first bars), with the associated compartments (second bars), bacterial replication rates (third bars), and oxygen level/conditions (fourth bars). To establish persistence, *Mtb* employs several effector mechanisms within the host cells and the granuloma. TB, tuberculosis; NO, nitric oxide; RNS, reactive nitrogen species; ROS, reactive oxygen species. Created with BioRender.com.

*Mtb* is a prototroph that can synthesize vitamins, amino acids, and co-factors (2) and is preferentially an intracellular pathogen (101). *Mtb* divides every 16–24 hours; this is a significantly slower rate than most bacteria, which divide on the order of minutes (74). Following infection, *Mtb* undergoes several rounds of rapid replication, which is then slowed or arrested by host immunity (2) (**Figure 6**).

### 8.2 *Mtb* immunoevasive mechanisms

Being a facultative intracellular bacterium, *Mtb* has developed immunoevasive mechanisms to circumvent the host’s immune response and forge its longevity in an otherwise hostile environment (33). To establish persistence, *Mtb* employs several effector mechanisms within the host cells (102). This includes inhibiting phagosome acidification, blocking phagolysosomal fusion, interfering with cellular trafficking, impairing apoptosis and autophagy, delaying antigen presentation, disrupting immune recognition, and modulating inflammasome activation (102). *Mtb* often resists immune protection by preventing phagosome maturation; this allows mycobacterial replication to persist, thereby propagating granuloma formation (95). Furthermore, it has been demonstrated that a portion of the *Mtb* population can induce phagosomal rupture; this allows mycobacteria to escape to the cytosol, promoting bacterial replication and host cell death via necrosis (95). Thus, it has been shown that the response in the phagocytosing macrophage could influence disease outcome (53).

Moreover, bacterial evasion of DC functions allows the persistent slowly replicating *Mtb* to bypass adaptive immunity (71). In this context, *Mtb* arrests DC maturation, antigen presentation, and cytokine production; this, in turn, undermines the magnitude of the T-cell response to engender bacterial survival in the lungs (71). In human studies, evidence has shown that the ability of *Mtb*-infected DCs to stimulate T-cell proliferation is impaired (71). Such mechanisms are paramount to the longevity of this unique mycobacterium.

### 8.3 Aerobic versus hypoxic niches

*Mtb* resides in a very specific nutritional environment and is reliant on host-derived nutrients obtained from distinct metabolic pathways (95). During infection, the range of carbon and nitrogen sources, and fluctuations in oxygen concentration that *Mtb* encounters affect the lipid composition of the bacterial envelope (95). Hence, metabolism is essential to host–pathogen dynamics (2). *Mtb* manipulation of the host immune response affords the bacteria essential metabolic capabilities to survive in diverse environments elicited by fluctuating nutrient availability (2) to aid its adaptation within hosts and transmission between them (3).

In the context of metabolism, glucose and triacylglycerides are thought to be the primary carbon sources under aerobic conditions; this occurs during early replication and immune response (2). While abrupt changes in oxygen levels threaten bacterial survival, with gradual adaptation, *Mtb* can persist in both non-replicating and slowly replicating states (even with antibiotic tolerance) in hypoxic conditions (oxygen levels as low as 0.03%) for decades (3). In the case of non-replicating bacilli, sufficient basal metabolic activity must be retained—this sustains an energized bacterial cell membrane and cellular processes to ensure survival (2).

As the infection proceeds, posing greater immune stress to the bacteria, *Mtb* switches metabolism from normal aerobic respiration to alternative (anaerobic type) pathways (2). As a result of glucose deficiency, mycobacteria shift to using lipids as the primary carbon source (2). Host lipids are nutrient sources for both host cells and *Mtb*; thus, the modulation of lipid homeostasis determines infection outcome (95). More specifically, the modulation of lipid homeostasis allows *Mtb* to, paradoxically, cause tissue necrosis and propagate transmission in some hosts while also causing persistent infection in other hosts who remain clinically asymptomatic for decades to a lifetime (3). As such, TB disease states are characterized by rapidly replicating (aerobic conditions), slowly replicating, and non-replicating bacterial phenotypes (3).

### 8.4 Bacterial manipulation in the granuloma

This prompts discussion on *Mtb* survival within the granuloma as demonstrated in animal models. *Mtb* responds to the granuloma’s imposition of nutrient starvation, ROS/NOS, and hypoxia using host cholesterol and employing a mycobacterial proteasome to help combat nitrosative and oxidative stress (97). Parenthetically, a zebrafish model of TB has also shown that, the granuloma promotes bacterial expansion by inducing infected macrophage apoptosis and recruiting uninfected macrophages to nascent granulomas (89). The newly recruited macrophages migrate to the growing granuloma where they uptake the apoptotic debris and bacterial contents of infected macrophages (89). In humans, the formation of FMs is a clear consequence of bacterial manipulation of the host metabolism, promoting the accumulation of lipids (95). The role of cholesterol, fatty acids, and their derivatives in the development of FMs that contribute to mycobacterial persistence is well evidenced (103).

## 9 Immune response and implications in disease relapse: drug development opportunities

Taking these findings, we can assess their implications in disease relapse. Relapse continues to be problematic owing to the empirical approach to treatment shortening in Phase 2 and 3 trials (20). There is mounting evidence that a more effective drug regimen is a pressing need for successful TB treatment, which is currently complicated by relapse and drug-resistant strains of *Mtb* (104). Management of the causative persister bacteria would moderate drug resistance/relapse, thereby allowing for shorter treatment durations. The extent to which drugs, as opposed to the immune system, can clear residual bacteria post-treatment, therefore, requires further clarification.

In the clinical space, the elusiveness of *Mtb* infection in humans, in conjunction with our incomplete understanding of the biomarkers of immunity against *Mtb* in infected patients, has limited the formulation of preventive strategies (39). Thus, we detail the innate response distinctly. Innate immunity is vital for early protection against TB, as it heavily determines the establishment of infection and disease development (105). However, the most successful combative agency against *Mtb* in individuals is the adaptive immune response, which prevents approximately 90%–95% of infected patients from developing active disease. The adaptive immune response can also deter the reactivation of TBI. Thus far, no vaccine has been able to demonstrate the same effect (39).

However, beyond vaccines, evidence does show that antibiotics like bedaquiline interact with human immune cells to forge immune-enhancing properties. This provides a potential longer-term objective for drug development (21). We can determine whether the future of TB treatment will involve a combination of drugs and immune modulators, or drugs and vaccines as an immune-boosting scheme. Hence, a comprehensive description of the immune response in humans can highlight the need for additional data collection (e.g., immune signaling triggered by bacterial debris in humans), which can result in an antibody response. Fundamentally, these data may provide an impetus for novel drug/regimen development.

## 10 Application of the data: modeling and simulation

To this end, the application of this work may pertain to the M&S sphere, involving the mathematical composition of the immune response to *Mtb* for modelers, such that the important components of the immune response to *Mtb* and the time course by which response occurs can be parameterized in mechanistic or semi-mechanistic models. This can better allegorize the host–pathogen–drug relationship. For instance, agent-based models that warrant a comprehensive understanding of the immune response timing may use the outlined animal data to simulate human immunity. For reliable translation, we must also understand the species differences between preclinical models and humans (e.g., signaling differences) to effectively simulate human response and ensure robust predictivity in the clinical setting, albeit the optimal use of translational simulations in antitubercular drug development has yet to be leveraged (106).

M&S offers new opportunities to explore immune dynamics using a multiscale and multiorgan approach with adjustable resolution (74), yielding good accuracy and reliability of simulation results. Preceding clinical evaluation (i.e., human trials), these simulations may comprise various clinical scenarios that can be systematically analyzed to establish exposure–response relationships and optimal trial design and to assess comparative exposures, safety, and efficacy of candidate compounds. This could define the efficacious dose range and shortened treatment durations that may eradicate different bacterial phenotypes in Phase 2 clinical studies (23). Essentially, translational efforts can inform drug development for relapse prevention using a systematic and scientifically grounded approach that diverges from empiricism. They can also provide mechanistic insights to enhance our understanding of the mechanisms that may contribute to relapse; and M&S may help to predict the risk of relapse.

While the provision of drug treatment and numerical data (e.g., immune cell numbers, cytokine concentrations, and treatment effect) is beyond the remit of this paper, **Supplementary Table 4** provides a list of references that contain this information to support model development.

## 11 Discussion

### 11.1 Summary

This narrative review provides a systematic purview of the key immune players that are implicated in human pulmonary TB, in line with the organs or compartments in which they reside. We also cover the bacterial immunoevasive mechanisms, their metabolic adaptation, and the presence of different bacterial phenotypes that enforce their persistence in this pernicious disease. This is coupled with the time course of response in animal models to add informative value for (semi-)mechanistic parametric considerations.

The key cellular players of the innate response include AECs, alveolar epithelial cells, neutrophils, NK cells, macrophages, and DCs. They induce bacterial elimination through various mechanisms: secretion of antimicrobial effector molecules to recruit immune cells to the site of infection by AECs (22) and alveolar epithelial cells (40), respiratory burst by neutrophils (31), cytotoxic cell lysis by NK cells (35), and phagocytosis by phagocytes (66). Arguably, the macrophage is the most important player of innate immunity, as it exhibits critical early bacterial eradication properties while also producing mediators that perpetuate the immune activity of other players in both arms of response. Evidence shows that immunocompromised patients who exhibit poor macrophage response ultimately develop TB (66), thus illustrating that macrophages contribute critically to the early clearance of intracellular *Mtb* in humans to prevent active disease.

The key players of the adaptive response include CD4+ T cells, CD8+ T cells, and the granulomatous lesions. T cells enforce bacterial killing via macrophage activation, inducing infected macrophage apoptosis (34) and enhancing cytolytic activity (88) due to pre-stimulation (35). The function of the granuloma is juxtaposed—aiding in bacterial containment and sequestration on the one hand and prolonging pathogenic survival on the other (92). CD4+ T cells have been shown to elicit the most robust bacterial killing in the adaptive response in humans due to their production of the pro-inflammatory cytokine, IFN-γ, which activates macrophages in the adaptive phase to eliminate bacteria (most efficiently) at the later stages of infection (66).

The key mediators, in addition to the pro-inflammatory cytokine, IFN-γ, include the pro-inflammatory cytokine, TNF-α, which ubiquitously recruits and activates other immune cells (22), while a significant anti-inflammatory cytokine, IL-10, is involved in immune modulation to prevent host-induced tissue damage (67).

### 11.2 Further applications of this review: vaccines and immunomodulation

Beyond M&S, we must note that a foremost goal in TB treatment has been the development of a new, effective vaccine. The Bacillus Calmette-Guérin (BCG) vaccine has been widely used as part of the expanded program of immunization in several countries (107). However, the efficacy of the BCG vaccine has demonstrated high variability, affording only a transient immune response against the TB challenge (108). Thus, research is focused on discovering new approaches to stimulate adaptive immunity, namely, improving the T-cell response against *Mtb* (77). This may be crucial to eradicating residual bacteria, wherein CD4+ T cells are paramount in pathogenic killing and therefore prevention of active disease (78).

Conversely, some argue that vaccine development strategies should consider the bacterial immunoevasive mechanisms instead, as the adaptive immunity-boosting approach has been largely unsuccessful to date (64). Studies indicate that enhancing phagolysosomal fusion, autophagy, and ROS production would open interesting explorative avenues for antitubercular efforts (64). It is thought that formulating vaccines to augment the innate response may be feasible. For instance, by altering macrophage interactions to eradicate *Mtb* at the earliest manifestations of infection (64).

Furthermore, it is recognized that anti-TB drugs alone are unlikely to eradicate *Mtb* in all cases, a challenge that is further complicated by the lengthy drug development process and the emergence of new drug-resistant strains, hence underscoring the need for immunomodulation strategies (109). A key example involves adjunct host-directed therapy (HDT)—this is an emerging treatment approach that represents a paradigm shift in the antitubercular landscape. In addition to targeting bacteria, HDTs aim to modulate the host immune response (110). Thus far, repurposed drugs for HDT have shown promise (109). For instance, a tyrosinase kinase inhibitor (imatinib) more effectively regulates *Mtb* uptake and killing in combination with anti-TB drugs, and vitamin D3 enhances macrophage activity (18). Another example is metformin, which enhances CD8+ T-cell activity, facilitates phagolysosomal fusion, and limits inflammation in patients with TB-HIV co-infection (109). That is, HDTs have been shown to alter the immune response, combat resistance, target new mechanisms, or shorten treatment duration to improve patient compliance (18). However, some researchers state that the HDTs currently under assessment are complicated by adverse events and high costs. Alternatively, natural products such as alkaloids and phenols are also being evaluated for their immunomodulating properties (like their ability to regulate macrophage polarization and upregulate Th1 lymphocytes), their lower immunopathological damage, and the their lower costs (109).

In summary, a better understanding of the host immune defense against *Mtb* may support the development of effective vaccines, HDTs, and, in turn, biomarkers, thereby reducing the need for the presently available resistance-laden antibiotics in TB treatment (40). This has yet to be harnessed in TB therapy.

### 11.3 Further implications of this review: immunosuppressed and immunocompromised patients

Critical to understanding the human immune response to *Mtb* is examining individuals with a suppressed or compromised immune response. For instance, patients taking immunosuppressive treatment like TNF-α inhibitors for inflammatory conditions such as rheumatoid arthritis (i.e., immunosuppressed due to medical intervention) (111) and those with autoimmune diseases like systemic lupus erythematosus (i.e., immunocompromised due to co-morbidities), are at an increased risk of TB resulting from a diminished immune response (112).

A significant co-morbidity in TB is HIV whereby patients are at a high risk of developing TB due to lower T-cell counts (78). Arguably, more importantly, the dual burden with diabetes mellitus (DM)—a leading cause of death worldwide—significantly impacts the progression and management of TB disease (113). Essentially, poor glycemic control in DM impairs immunity, thereby increasing susceptibility to TB (114). That is, the metabolic alterations characteristic of DM dysregulate cell-mediated immunity and cytokine secretion (115). More specifically, type 2 DM (the more common form) is a well-recognized risk factor for TB (116). Although debatable, some evidence suggests that active TB may, reciprocally, trigger type 2 diabetes through impaired glucose tolerance (116). Due to the complex and multifaceted nature of this metabolic disorder (116), the clinical presentation of TB-DM is also more complex; patients often present with higher severity and more cavitary disease (115). Further, DM causes pharmacokinetic variability in TB drugs, leading to suboptimal drug exposure and, consequently, compromised treatment outcomes (117). As such, patients with TB-DM co-morbidity have increased risks of active TB incidence by threefold (118), disease relapse by twofold, MDR-TB by twofold (119), and mortality by twofold (114). Given the rising incidence of DM (116), particularly type 2 DM (119), predominantly in developing countries where the TB burden is already markedly higher, the TB-DM dual burden is becoming even more frequent (116). Thus, it is crucial to understand the factors that enable effective TB-DM treatment (116).

While this review focuses on the immune response of immunocompetent patients before drug treatment, a greater understanding and application of the data provided, like the time course of response as outlined herein (mechanistically or semi-mechanistically) in conjunction with the cellular and molecular immune processes in humans, may provide insight into the causes of insufficient response that contribute to relapse. This could serve as a baseline for research whereby immunosuppressed and immunocompromised patients are subsequently considered. By comparing their immune responses to those of immunocompetent patients, the findings may support the understanding of key differences—such as compromised mechanisms—in the presence of co-morbidities.

### 11.4 Limitations

#### 11.4.1 Time course (animal data)

A key limitation is that since human temporal data are scarce, preclinical data were used to characterize the time course of response. Although cynomolgus macaques closely resemble various manifestations of human TB (120), no animal model can completely represent human disease (24). This limitation underscores the challenges in replicating the full spectrum of human immune responses in animal models (24), including the time course. Nonetheless, the advent of *in silico* models has supported the translation of preclinical data to inform human immune response, providing knowledge that is otherwise limited in experimental systems (25).

Similarly, the extracted papers covered only a 12-week span of immune response (as mice often die within experimental timelines)—perhaps a longer duration of data that are found in NHPs would be better suited to understand human timing as opposed to using predominantly mouse data. Nonetheless, it is broadly recognized that murine data have proven vital to understanding TB pathology and protective immunity mechanisms (24). Veritably, much of our understanding of T-cell function in tuberculous lungs is also derived from murine studies (26).

#### 11.4.2 Granuloma (limited human data)

Additionally, our knowledge of the granuloma response in humans is limited by the ethical constraints surrounding lung autopsies. In TB research, a lung autopsy is conducted on individuals who died in a TB-endemic setting (39). Such studies offer invaluable insights into the intricate dynamics of this infection. They aid in the recognition of distinctive characteristics and support our understanding of disease control (24). However, they are compounded by a myriad of challenges, including obtaining consent and limited resources (27). Moreover, the use of patient blood samples to understand lesional response provides little indication, and the complex pathophysiology of granuloma formation cannot yet be directly investigated at the molecular level within human lesions (99). A further limitation is in obtaining lung samples via biopsy for patients with suspected TB; given that symptoms may not present for months to years, the investigation of lesion development is further impeded (28). Therefore, several preclinical models, including mice, guinea pigs, rabbits, mini pigs, tamarins, and NHPs, are used in the research arena. Together, these models may aid in demonstrating the complicated pathophysiology in humans (99).

#### 11.4.3 Translation of preclinical models to humans

Regarding the translation of preclinical data to humans for granuloma response, the granulomatous infiltration in the lungs of mice lacks the structured and organized appearance that is commonly seen in human lesions. However, those found in guinea pigs and rabbits exhibit a more human-like structure. Notably, *Mtb*-infected macaques closely emulate the comprehensive range of granuloma forms found in patients. Owing to this, coupled with translational principles like scaling for species differences, research conducted on NHPs has proven integral to understanding the development and structure of human lesions (25).

### 11.5 Concluding remarks

This review succinctly encapsulates the immune response to *Mtb* in humans. Although our knowledge of the full spectrum of response (particularly the granuloma) is incipient—specifically at the molecular level—the data presented may aid in drug development, and wider fields, as they offer a structured and broad vignette of cellular and molecular immunological mechanisms in response to *Mtb*. Integrating these comprehensive insights into a cohesive framework with M&S could help elucidate the mechanisms behind relapse, predict the risk of relapse, and inform potential treatment strategies. We also provide a significance ranking of the qualitative data to put the role of each player into a broader perspective and cover the key information in a single report. Additionally, we endorse the use of translational simulations to support the widely recognized, critical need for novel treatment approaches in the antitubercular armamentarium.

## References

[1] Furin J Cox H Pai M. Tuberculosis. Lancet. (2019) 393:1642–56. doi: 10.1016/S0140-6736(19)30308-3 30904262

[2] Warner DF. Mycobacterium tuberculosis metabolism. Cold Spring Harb Perspect Med. (2014) 5:a021121. doi: 10.1101/cshperspect.a021121 25502746 PMC4382733

[3] Ehrt S Schnappinger D Rhee KY. Metabolic principles of persistence and pathogenicity in Mycobacterium tuberculosis. Nat Rev Microbiol. (2018) 16:496–507. doi: 10.1038/s41579-018-0013-4 29691481 PMC6045436

[4] Ganguli S Gammack D Kirschner DE. A metapopulation model of granuloma formation in the lung during infection with mycobacterium tuberculosis. Math Biosci Eng. (2005) 2:535–60. doi: 10.3934/mbe.2005.2.535 20369939

[5] de Martino M Lodi L Galli L Chiappini E. Immune response to mycobacterium tuberculosis: A narrative review. Front Pediatr. (2019) 7:350. doi: 10.3389/fped.2019.00350 31508399 PMC6718705

[6] Coussens AK Zaidi SMA Allwood BW Dewan PK Gray G Kohli M. Classification of early tuberculosis states to guide research for improved care and prevention: an international Delphi consensus exercise. Lancet Respir Med. (2024) 12:484–98. doi: 10.1016/S2213-2600(24)00028-6 PMC7616323 38527485

[7] Jilani TN Avula A Zafar Gondal A Siddiqui AH. Active tuberculosis. In: StatPearls. StatPearls Publishing, Treasure Island (FL (2021). Available at: http://www.ncbi.nlm.nih.gov/books/NBK513246/. 30020618

[8] Peralta Alvarez MP Marshall JL Tanner R. Correlates of Protection from Tuberculosis. In: Christodoulides M, editor. Vaccines for Neglected Pathogens: Strategies, Achievements and Challenges. Springer International Publishing, Cham (2023). p. 99–137. doi: 10.1007/978-3-031-24355-4_6

[9] Goletti D Lindestam Arlehamn CS Scriba TJ Anthony R Cirillo DM Alonzi T. Can we predict tuberculosis cure? What tools are available? Eur Respir J. (2018) 52:1801089. doi: 10.1183/13993003.01089-2018 30361242

[10] Beltran CGG Heunis T Gallant J Venter R du Plessis N Loxton AG. Investigating non-sterilizing cure in TB patients at the end of successful anti-TB therapy. Front Cell Infect Microbiol. (2020) 10:443. doi: 10.3389/fcimb.2020.00443 32984071 PMC7477326

[11] Singh V Chibale K. Strategies to combat multi-drug resistance in tuberculosis. Acc Chem Res. (2021) 54:2361–76. doi: 10.1021/acs.accounts.0c00878 PMC8154215 33886255

[12] Tiberi S Muñoz-Torrico M Duarte R Dalcolmo M D’Ambrosio L Migliori GB. New drugs and perspectives for new anti-tuberculosis regimens. Pulmonology. (2018) 24:86–98. doi: 10.1016/j.rppnen.2017.10.009 29487031

[13] Larkins-Ford J Degefu YN Van N Sokolov A Aldridge BB. Design principles to assemble drug combinations for effective tuberculosis therapy using interpretable pairwise drug response measurements. Cell Rep Med. (2022) 3:100737. doi: 10.1016/j.xcrm.2022.100737 36084643 PMC9512659

[14] Greenstein T Aldridge BB. Tools to develop antibiotic combinations that target drug tolerance in Mycobacterium tuberculosis. Front Cell Infect Microbiol. (2023) 12:1085946. doi: 10.3389/fcimb.2022.1085946 36733851 PMC9888313

[15] Colangeli R Jedrey H Kim S Connell R Ma S Chippada Venkata UD. Bacterial factors that predict relapse after tuberculosis therapy. N Engl J Med. (2018) 379:823–33. doi: 10.1056/NEJMoa1715849 PMC6317071 30157391

[16] WHO consolidated guidelines on tuberculosis. Module 4: treatment - drug-resistant tuberculosis treatment. Geneva: World Health Organization (2022). Licence: CC BY-NC-SA 3.0 IGO. 32603040

[17] Vanino E Granozzi B Akkerman OW Munoz-Torrico M Palmieri F Seaworth B. Update of drug-resistant tuberculosis treatment guidelines: A turning point. Int J Infect Dis. (2023) 130:S12–5. doi: 10.1016/j.ijid.2023.03.013 36918080

[18] Abreu R Giri P Quinn F. Host-pathogen interaction as a novel target for host-directed therapies in tuberculosis. Front Immunol. (2020) 11:1553. doi: 10.3389/fimmu.2020.01553 32849525 PMC7396704

[19] Bartelink I Zhang N Keizer R Strydom N Converse P Dooley K. New paradigm for translational modeling to predict long-term tuberculosis treatment response. Clin Trans Sci. (2017) 10:366–79. doi: 10.1111/cts.2017.10.issue-5 PMC5593171 28561946

[20] Muliaditan M Davies GR Simonsson USH Gillespie SH Della Pasqua O. The implications of model-informed drug discovery and development for tuberculosis. Drug Discovery Today. (2017) 22:481–6. doi: 10.1016/j.drudis.2016.09.004 27693714

[21] Giraud-Gatineau A Coya JM Maure A Biton A Thomson M Bernard EM. The antibiotic bedaquiline activates host macrophage innate immune resistance to bacterial infection. Elife. (2020) 9:e55692. doi: 10.7554/eLife.55692 32369020 PMC7200153

[22] Gupta N Kumar R Agrawal B. New players in immunity to tuberculosis: the host microbiome, lung epithelium, and innate immune cells. Front Immunol. (2018) 9:709. doi: 10.3389/fimmu.2018.00709 29692778 PMC5902499

[23] Chen C Ortega F Alameda L Simonsson USH. Population pharmacokinetics, optimised design and sample size determination for rifampicin, isoniazid, ethambutol and pyrazinamide in the mouse. Eur J Pharm Sci. (2016) 93:319–33. doi: 10.1016/j.ejps.2016.07.017 27473307

[24] Kaushal D Mehra S Didier PJ Lackner AA. The non-human primate model of tuberculosis: Primate model of TB. J Med Primatol. (2012) 41:191–201. doi: 10.1111/j.1600-0684.2012.00536.x 22429048 PMC3961469

[25] Flynn JL Chan J Lin PL. Macrophages and control of granulomatous inflammation in tuberculosis. Mucosal Immunol. (2011) 4:271–8. doi: 10.1038/mi.2011.14 PMC3311958 21430653

[26] Gideon HP Phuah J Myers AJ Bryson BD Rodgers MA Coleman MT. Variability in tuberculosis granuloma T cell responses exists, but a balance of pro- and anti-inflammatory cytokines is associated with sterilization. PloS Pathog. (2015) 11:e1004603. doi: 10.1371/journal.ppat.1004603 25611466 PMC4303275

[27] Karat AS Omar T Tlali M Charalambous S Chihota VN Churchyard GJ. Lessons learnt conducting minimally invasive autopsies in private mortuaries as part of HIV and tuberculosis research in South Africa. Public Health action. (2019) 9:186–90. doi: 10.5588/pha.19.0032 PMC6945733 32042614

[28] Hunter L Hingley-Wilson S Stewart GR Sharpe SA Salguero FJ. Dynamics of macrophage, T and B cell infiltration within pulmonary granulomas induced by mycobacterium tuberculosis in two non-human primate models of aerosol infection. Front Immunol. (2022) 12:776913. doi: 10.3389/fimmu.2021.776913 35069548 PMC8770544

[29] Sawyer AJ Patrick E Edwards J Wilmott JS Fielder T Yang Q. Spatial mapping reveals granuloma diversity and histopathological superstructure in human tuberculosis. J Exp Med. (2023) 220:e20221392. doi: 10.1084/jem.20221392 36920308 PMC10035589

[30] Corleis B Dorhoi A. Early dynamics of innate immunity during pulmonary tuberculosis. Immunol Letters. (2020) 221:56–60. doi: 10.1016/j.imlet.2020.02.010 32092359

[31] Liu CH Liu H Ge B. Innate immunity in tuberculosis: host defense vs pathogen evasion. Cell Mol Immunol. (2017) 14:963–75. doi: 10.1038/cmi.2017.88 PMC5719146 28890547

[32] Ramakrishnan L. Revisiting the role of the granuloma in tuberculosis. Nat Rev Immunol. (2012) 12:352–66. doi: 10.1038/nri3211 22517424

[33] Weiss G Schaible UE. Macrophage defense mechanisms against intracellular bacteria. Immunol Rev. (2015) 264:182–203. doi: 10.1111/imr.2015.264.issue-1 25703560 PMC4368383

[34] Sud D Bigbee C Flynn JL Kirschner DE. Contribution of CD8+ T cells to control of Mycobacterium tuberculosis infection. J Immunol. (2006) 176:4296–314. doi: 10.4049/jimmunol.176.7.4296 16547267

[35] Nutt SL Huntington ND. Cytotoxic T Lymphocytes and Natural Killer Cells. In: Clinical Immunology. Amsterdam, Netherlands: Elsevier (2019). p. 247–259.e1. Available at: https://linkinghub.elsevier.com/retrieve/pii/B978070206896600017X.

[36] Huang L Nazarova EV Russell DG. Mycobacterium tuberculosis: Bacterial Fitness within the Host Macrophage. Microbiol Spectr. (2019) 7. doi: 10.1128/microbiolspec.BAI-0001-2019 PMC6459685 30848232

[37] Pennisi M Russo G Sgroi G Bonaccorso A Parasiliti Palumbo GA Fichera E. Predicting the artificial immunity induced by RUTI^®^ vaccine against tuberculosis using universal immune system simulator (UISS). BMC Bioinf. (2019) 20:504. doi: 10.1186/s12859-019-3045-5 PMC6904993 31822272

[38] Linderman JJ Cilfone NA Pienaar E Gong C Kirschner DE. A multi-scale approach to designing therapeutics for tuberculosis. Integr Biol (Camb). (2015) 7:591–609. doi: 10.1039/c4ib00295d 25924949 PMC4436084

[39] Ryndak MB Laal S. Mycobacterium tuberculosis primary infection and dissemination: A critical role for alveolar epithelial cells. Front Cell Infect Microbiol. (2019) 9:299. doi: 10.3389/fcimb.2019.00299 31497538 PMC6712944

[40] Chai Q Lu Z Liu CH. Host defense mechanisms against Mycobacterium tuberculosis. Cell Mol Life Sci. (2020a) 77:1859–78. doi: 10.1007/s00018-019-03353-5 PMC11104961 31720742

[41] Marino S Hult C Wolberg P Linderman JJ Kirschner DE. The role of dimensionality in understanding granuloma formation. Comput (Basel). (2018) 6. doi: 10.3390/computation6040058 PMC6599587 31258937

[42] Mattila JT Ojo OO Kepka-Lenhart D Marino S Kim JH Eum SY. Microenvironments in tuberculous granulomas are delineated by distinct populations of macrophage subsets and expression of nitric oxide synthase and arginase isoforms. J Immunol. (2013) 191:773–84. doi: 10.4049/jimmunol.1300113 PMC3746594 23749634

[43] Wong EA Evans S Kraus CR Engelman KD Maiello P Flores WJ. IL-10 Impairs Local Immune Response in Lung Granulomas and Lymph Nodes during Early Mycobacterium tuberculosis Infection. J Immunol. (2020) 204:644–59. doi: 10.4049/jimmunol.1901211 PMC6981067 31862711

[44] Li Y Wang Y Liu X. The role of airway epithelial cells in response to mycobacteria infection. Clin Dev Immunol. (2012) 2012:1–11. doi: 10.1155/2012/791392 PMC3337601 22570668

[45] Rodrigues TS Conti BJ Fraga-Silva TFDC Almeida F Bonato VLD. Interplay between alveolar epithelial and dendritic cells and Mycobacterium tuberculosis. J Leukocyte Biol. (2020) 108:1139–56. doi: 10.1002/JLB.4MR0520-112R 32620048

[46] Condon TV Sawyer RT Fenton MJ Riches DWH. Lung dendritic cells at the innate-adaptive immune interface. J Leukocyte Biol. (2011) 90:883–95. doi: 10.1189/jlb.0311134 PMC3206474 21807741

[47] Barclay AM Ninaber DK Van Veen S Hiemstra PS Ottenhoff THM van der Does AM. Airway epithelial cells mount an early response to mycobacterial infection. Front Cell Infect Microbiol. (2023) 13:1253037. doi: 10.3389/fcimb.2023.1253037 37822359 PMC10562574

[48] Vono M Lin A Norrby-Teglund A Koup RA Liang F Loré K. Neutrophils acquire the capacity for antigen presentation to memory CD4+ T cells *in vitro* and *ex vivo* . Blood. (2017) 129:1991–2001. doi: 10.1182/blood-2016-10-744441 28143882 PMC5383872

[49] Bussi C Gutierrez MG. Mycobacterium tuberculosis infection of host cells in space and time. FEMS Microbiol Rev. (2019) 43:341–61. doi: 10.1093/femsre/fuz006 PMC6606852 30916769

[50] Gammack D Doering CR Kirschner DE. Macrophage response to Mycobacterium tuberculosis infection. J Math Biol. (2004) 48:218–42. doi: 10.1007/s00285-003-0232-8 14745511

[51] Nisa A Kipper FC Panigrahy D Tiwari S Kupz A Subbian S. Different modalities of host cell death and their impact on Mycobacterium tuberculosis infection. Am J Physiology-Cell Physiol. (2022) 323:C1444–74. doi: 10.1152/ajpcell.00246.2022 PMC9662802 36189975

[52] Sokol CL Luster AD. The chemokine system in innate immunity. Cold Spring Harb Perspect Biol. (2015) 7:a016303. doi: 10.1101/cshperspect.a016303 25635046 PMC4448619

[53] Lowe DM Redford PS Wilkinson RJ O’Garra A Martineau AR. Neutrophils in tuberculosis: friend or foe? Trends Immunol. (2012) 33:14–25. doi: 10.1016/j.it.2011.10.003 22094048

[54] Nwongbouwoh Muefong C Owolabi O Donkor S Charalambous S Bakuli A Rachow A. Neutrophils contribute to severity of tuberculosis pathology and recovery from lung damage pre- and posttreatment. Clin Infect Dis. (2022) 74:1757–66. doi: 10.1093/cid/ciab729 PMC9155606 34427644

[55] Mak TW Saunders ME. B Cell Receptor Structure and Effector Function. In: The Immune Response [Internet]. Elsevier: Amsterdam, Netherlands; Boston, USA. (2006) 10:93–120. Available at: https://linkinghub.elsevier.com/retrieve/pii/B9780120884513500077.

[56] Dallenga T Repnik U Corleis B Eich J Reimer R Griffiths GW. M. tuberculosis-induced necrosis of infected neutrophils promotes bacterial growth following phagocytosis by macrophages. Cell Host Microbe. (2017) 22:519–530.e3. doi: 10.1016/j.chom.2017.09.003 29024644

[57] Borkute RR Woelke S Pei G Dorhoi A. Neutrophils in tuberculosis: cell biology, cellular networking and multitasking in host defense. IJMS. (2021) 22:4801. doi: 10.3390/ijms22094801 33946542 PMC8125784

[58] Muefong CN Sutherland JS. Neutrophils in tuberculosis-associated inflammation and lung pathology. Front Immunol. (2020) 11:962. doi: 10.3389/fimmu.2020.00962 32536917 PMC7266980

[59] Hilda JN Das S Tripathy SP Hanna LE. Role of neutrophils in tuberculosis: A bird’s eye view. Innate Immun. (2020) 26:240–7. doi: 10.1177/1753425919881176 PMC7251797 31735099

[60] Li Y Wang W Yang F Xu Y Feng C Zhao Y. The regulatory roles of neutrophils in adaptive immunity. Cell Commun Signal. (2019) 17:147. doi: 10.1186/s12964-019-0471-y 31727175 PMC6854633

[61] Qin Y Wang Q Shi J. Immune checkpoint modulating T cells and NK cells response to Mycobacterium tuberculosis infection. Microbiol Res. (2023) 273:127393. doi: 10.1016/j.micres.2023.127393 37182283

[62] Lin PL Flynn JL. CD8 T cells and Mycobacterium tuberculosis infection. Semin Immunopathol. (2015) 37:239–49. doi: 10.1007/s00281-015-0490-8 PMC4439333 25917388

[63] Murphy K Weaver C Berg L Janeway C. Janeway’s immunobiology. 10th edition. New York, NY: W.W. Norton and Company (2022).

[64] Chandra P Grigsby SJ Philips JA. Immune evasion and provocation by Mycobacterium tuberculosis. Nat Rev Microbiol. (2022) 20:750–66. doi: 10.1038/s41579-022-00763-4 PMC9310001 35879556

[65] Guirado E Schlesinger LS Kaplan G. Macrophages in tuberculosis: friend or foe. Semin Immunopathol. (2013) 35:563–83. doi: 10.1007/s00281-013-0388-2 PMC3763202 23864058

[66] Chaurasiya SK. Tuberculosis: Smart manipulation of a lethal host: Survival Inside Killers. Microbiol Immunol. (2018) 62:361–79. doi: 10.1111/1348-0421.12593 29687912

[67] Cilfone NA Ford CB Marino S Mattila JT Gideon HP Flynn JL. Computational modeling predicts IL-10 control of lesion sterilization by balancing early host immunity-mediated antimicrobial responses with caseation during mycobacterium tuberculosis infection. J Immunol. (2015) 194:664–77. doi: 10.4049/jimmunol.1400734 PMC4283220 25512604

[68] Pedruzzi G Das PN Rao KVS Chatterjee S. Understanding PGE2, LXA4 and LTB4 balance during Mycobacterium tuberculosis infection through mathematical model. J Theor Biol. (2016) 389:159–70. doi: 10.1016/j.jtbi.2015.10.025 26551160

[69] Wigginton JE Kirschner D. A model to predict cell-mediated immune regulatory mechanisms during human infection with Mycobacterium tuberculosis. J Immunol. (2001) 166:1951–67. doi: 10.4049/jimmunol.166.3.1951 11160244

[70] Kim H Shin SJ. Pathological and protective roles of dendritic cells in Mycobacterium tuberculosis infection: Interaction between host immune responses and pathogen evasion. Front Cell Infect Microbiol. (2022) 12:891878. doi: 10.3389/fcimb.2022.891878 35967869 PMC9366614

[71] Sia JK Georgieva M Rengarajan J. Innate immune defenses in human tuberculosis: an overview of the interactions between mycobacterium tuberculosis and innate immune cells. J Immunol Res. (2015) 2015:1–12. doi: 10.1155/2015/747543 PMC4516846 26258152

[72] Marino S Kirschner DE. The human immune response to Mycobacterium tuberculosis in lung and lymph node. J Theor Biol. (2004) 227:463–86. doi: 10.1016/j.jtbi.2003.11.023 15038983

[73] Stillwell W. Moving Components Through the Cell. In: An Introduction to Biological Membranes. London, UK: Elsevier (2016). p. 369–79. Available at: https://linkinghub.elsevier.com/retrieve/pii/B9780444637727000178.

[74] Marino S Linderman JJ Kirschner DE. A multifaceted approach to modeling the immune response in tuberculosis. Wiley Interdiscip Rev Syst Biol Med. (2011) 3:479–89. doi: 10.1002/wsbm.v3.4 PMC3110521 21197656

[75] Marino S Pawar S Fuller CL Reinhart TA Flynn JL Kirschner DE. Dendritic cell trafficking and antigen presentation in the human immune response to Mycobacterium tuberculosis. J Immunol. (2004) 173:494–506. doi: 10.4049/jimmunol.173.1.494 15210810

[76] Lian J Luster AD. Chemokine-guided cell positioning in the lymph node orchestrates the generation of adaptive immune responses. Curr Opin Cell Biol. (2015) 36:1–6. doi: 10.1016/j.ceb.2015.05.003 26067148 PMC4639456

[77] Carty SA Riese MJ Koretzky GA. Chapter 21 - T-Cell Immunity. In: offman R Benz EJ Silberstein LE Heslop HE Weitz JI Anastasi J, editors. Hematology, Seventh Edition. Philadelphia, USA: Elsevier (2018). p. 221–39. Available at: https://www.sciencedirect.com/science/article/pii/B9780323357623000214.

[78] Bozzano F Marras F De Maria A. IMMUNOLOGY OF TUBERCULOSIS. Mediterr J Hematol Infect Dis. (2014) 6:e2014027. doi: 10.4084/mjhid.2014.027 24804000 PMC4010607

[79] Flynn JL Chan J. Immune cell interactions in tuberculosis. Cell. (2022) 185:4682–702. doi: 10.1016/j.cell.2022.10.025 PMC12162144 36493751

[80] Seder RA Ahmed R. Similarities and differences in CD4+ and CD8+ effector and memory T cell generation. Nat Immunol. (2003) 4:835–42. doi: 10.1038/ni969 12942084

[81] Capece T Kim M. The role of lymphatic niches in T cell differentiation. Mol Cells. (2016) 39:515–23. doi: 10.14348/molcells.2016.0089 PMC4959015 27306645

[82] Kuka M De Giovanni M Iannacone M. The role of type I interferons in CD4+ T cell differentiation. Immunol Lett. (2019) 215:19–23. doi: 10.1016/j.imlet.2019.01.013 30771379 PMC7234836

[83] Leal Rojas IM Mok WH Pearson FE Minoda Y Kenna TJ Barnard RT. Human blood CD1c+ Dendritic cells promote th1 and th17 effector function in memory CD4+ T cells. Front Immunol. (2017) 8:971. doi: 10.3389/fimmu.2017.00971 28878767 PMC5572390

[84] Khader SA Partida-Sanchez S Bell G Jelley-Gibbs DM Swain S Pearl JE. Interleukin 12p40 is required for dendritic cell migration and T cell priming after Mycobacterium tuberculosis infection. J Exp Med. (2006) 203:1805–15. doi: 10.1084/jem.20052545 PMC2118335 16818672

[85] Huang G Wang Y Chi H. Regulation of TH17 cell differentiation by innate immune signals. Cell Mol Immunol. (2012) 9:287–95. doi: 10.1038/cmi.2012.10 PMC3423893 22504954

[86] Lourenço EV La Cava A. Natural regulatory T cells in autoimmunity. Autoimmunity. (2011) 44:33–42. doi: 10.3109/08916931003782155 21091291 PMC3057884

[87] Herbert C Ahmadzai H Thomas PS. Proinflammatory and Regulatory Cytokines in Sarcoidosis. In: Cytokine Effector Functions in Tissues. London, UK: Elsevier (2017). p. 129–38. Available at: https://linkinghub.elsevier.com/retrieve/pii/B9780128042144000075.

[88] St. Paul M Ohashi PS. The roles of CD8+ T cell subsets in antitumor immunity. Trends Cell Biol. (2020) 30:695–704. doi: 10.1016/j.tcb.2020.06.003 32624246

[89] Pagán AJ Ramakrishnan L. Immunity and immunopathology in the tuberculous granuloma. Cold Spring Harb Perspect Med. (2015) 5:a018499. doi: 10.1101/cshperspect.a018499 PMC4561401 25377142

[90] Dooley KE Phillips PPJ Nahid P Hoelscher M. Challenges in the clinical assessment of novel tuberculosis drugs. Adv Drug Delivery Rev. (2016) 102:116–22. doi: 10.1016/j.addr.2016.01.014 PMC4903928 26827911

[91] Cronan MR. In the thick of it: formation of the tuberculous granuloma and its effects on host and therapeutic responses. Front Immunol. (2022) 13:820134. doi: 10.3389/fimmu.2022.820134 35320930 PMC8934850

[92] Linderman JJ Kirschner DE. *In silico* models of M. tuberculosis infection provide a route to new therapies. Drug Discovery Today Dis Models. (2015) 15:37–41. doi: 10.1016/j.ddmod.2014.02.006 PMC4758993 26904139

[93] Schaaf HS Zumla A eds. Tuberculosis: a comprehensive clinical reference. Edinburgh: Saunders, Elsevier (2009). 1014 p.

[94] Russell DG Cardona PJ Kim MJ Allain S Altare F. Foamy macrophages and the progression of the human tuberculosis granuloma. Nat Immunol. (2009) 10:943–8. doi: 10.1038/ni.1781 PMC2759071 19692995

[95] Gago G Diacovich L Gramajo H. Lipid metabolism and its implication in mycobacteria-host interaction. Curr Opin Microbiol. (2018) 41:36–42. doi: 10.1016/j.mib.2017.11.020 29190491 PMC5862736

[96] Lanni F Wijnant GJ Xie M Osiecki P Dartois V Sarathy JP. Adaptation to the intracellular environment of primary human macrophages influences drug susceptibility of Mycobacterium tuberculosis. Tuberculosis. (2023) 139:102318. doi: 10.1016/j.tube.2023.102318 36889104

[97] Huynh KK Joshi SA Brown EJ. A delicate dance: host response to mycobacteria. Curr Opin Immunol. (2011) 23:464–72. doi: 10.1016/j.coi.2011.06.002 21726990

[98] Evans S Butler JR Mattila JT Kirschner DE. Systems biology predicts that fibrosis in tuberculous granulomas may arise through macrophage-to-myofibroblast transformation. PloS Comput Biol. (2020) 16:e1008520. doi: 10.1371/journal.pcbi.1008520 33370784 PMC7793262

[99] Ehlers S Schaible UE. The granuloma in tuberculosis: dynamics of a host-pathogen collusion. Front Immunol. (2012) 3:411. doi: 10.3389/fimmu.2012.00411 23308075 PMC3538277

[100] Hortle E Oehlers SH. Host-directed therapies targeting the tuberculosis granuloma stroma. Pathog Dis. (2020) 78:ftaa015. doi: 10.1093/femspd/ftaa015 32149337

[101] Marino S Kirschner DE. A multi-compartment hybrid computational model predicts key roles for dendritic cells in tuberculosis infection. Comput (Basel). (2016) 4. doi: 10.3390/computation4040039 PMC5627612 28989808

[102] Upadhyay S Mittal E Philips JA. Tuberculosis and the art of macrophage manipulation. Pathog Dis. (2018) 76. doi: 10.1093/femspd/fty037/4970761 PMC6251593 29762680

[103] Mahajan S Dkhar HK Chandra V Dave S Nanduri R Janmeja AK. *Mycobacterium tuberculosis* modulates macrophage lipid-sensing nuclear receptors PPARγ and TR4 for survival. JI. (2012) 188:5593–603. doi: 10.4049/jimmunol.1103038 22544925

[104] Sotgiu G Centis R D’ambrosio L Migliori GB. Tuberculosis treatment and drug regimens. Cold Spring Harb Perspect Med. (2015) 5:a017822. doi: 10.1101/cshperspect.a017822 25573773 PMC4448591

[105] Chai Q Wang L Liu CH Ge B. New insights into the evasion of host innate immunity by Mycobacterium tuberculosis. Cell Mol Immunol. (2020b) 17:901–13. doi: 10.1038/s41423-020-0502-z PMC7608469 32728204

[106] Dooley KE Hanna D Mave V Eisenach K Savic RM. Advancing the development of new tuberculosis treatment regimens: The essential role of translational and clinical pharmacology and microbiology. PloS Med. (2019) 16:e1002842. doi: 10.1371/journal.pmed.1002842 31276490 PMC6611566

[107] Abu-Raddad LJ Sabatelli L Achterberg JT Sugimoto JD Longini IM Dye C. Epidemiological benefits of more-effective tuberculosis vaccines, drugs, and diagnostics. Proc Natl Acad Sci USA. (2009) 106:13980–5. doi: 10.1073/pnas.0901720106 PMC2720405 19666590

[108] Chen JM Alexander DC Behr MA Liu J. *Mycobacterium bovis* BCG vaccines exhibit defects in alanine and serine catabolism. Infect Immun. (2003) 71:708–16. doi: 10.1128/IAI.71.2.708-716.2003 PMC145370 12540549

[109] Huang X Lowrie DB Fan XY Hu Z. Natural products in anti-tuberculosis host-directed therapy. Biomed Pharmacother. (2024) 171:116087. doi: 10.1016/j.biopha.2023.116087 38171242

[110] Cubillos-Angulo JM Nogueira BMF Arriaga MB Barreto-Duarte B Araújo-Pereira M Fernandes CD. Host-directed therapies in pulmonary tuberculosis: Updates on anti-inflammatory drugs. Front Med. (2022) 9:970408. doi: 10.3389/fmed.2022.970408 PMC9537567 36213651

[111] Fallahi-Sichani M Flynn JL Linderman JJ Kirschner DE. Differential risk of tuberculosis reactivation among anti-TNF therapies is due to drug binding kinetics and permeability. J Immunol. (2012) 188:3169–78. doi: 10.4049/jimmunol.1103298 PMC3311778 22379032

[112] Shekhar Gupta NY Harisingani AR. Diagnosis of tuberculosis with autoimmune hepatitis–systemic lupus erythematosus overlap syndrome: a case report. J Med Case Rep. (2022) 16:428. doi: 10.1186/s13256-022-03572-8 36333730 PMC9636765

[113] Forsman LD Alffenaar JWC. Diabetes mellitus and TB – finding strategies to reduce the double burden of disease. Int J tuberc Lung Dis. (2023) 27:91–3. doi: 10.5588/ijtld.22.0619 36853116

[114] Zhao L Gao F Zheng C Sun X. The impact of optimal glycemic control on tuberculosis treatment outcomes in patients with diabetes mellitus: systematic review and meta-analysis. JMIR Public Health Surveill. (2024) 10:e53948. doi: 10.2196/53948 38564244 PMC11022131

[115] Abbas U Masood KI Khan A Irfan M Saifullah N Jamil B. Tuberculosis and diabetes mellitus: Relating immune impact of co-morbidity with challenges in disease management in high burden countries. J Clin Tubercul Other Mycobacterial Dis. (2022) 29:100343. doi: 10.1016/j.jctube.2022.100343 PMC9720570 36478777

[116] Bisht MK Dahiya P Ghosh S Mukhopadhyay S. The cause–effect relation of tuberculosis on incidence of diabetes mellitus. Front Cell Infect Microbiol. (2023) 13:1134036. doi: 10.3389/fcimb.2023.1134036 37434784 PMC10330781

[117] Alffenaar JWC Stocker SL Forsman LD Garcia-Prats A Heysell SK Aarnoutse RE. Clinical standards for the dosing and management of TB drugs. Int J tuberc Lung Dis. (2022) 26:483–99. doi: 10.5588/ijtld.22.0188 PMC9165737 35650702

[118] Buasroung P Petnak T Liwtanakitpipat P Kiertiburanakul S. Prevalence of diabetes mellitus in patients with tuberculosis: A prospective cohort study. Int J Infect Dis. (2022) 116:374–9. doi: 10.1016/j.ijid.2022.01.047 35093530

[119] Huangfu P Ugarte-Gil C Golub J Pearson F Critchley J. The effects of diabetes on tuberculosis treatment outcomes: an updated systematic review and meta-analysis. Int J tuberc Lung Dis. (2019) 23:783–96. doi: 10.5588/ijtld.18.0433 31439109

[120] Capuano SV Croix DA Pawar S Zinovik A Myers A Lin PL. Experimental *mycobacterium tuberculosis* infection of cynomolgus macaques closely resembles the various manifestations of human M. tuberculosis Infection. Infect Immun. (2003) 71:5831–44. doi: 10.1128/IAI.71.10.5831-5844.2003 PMC201048 14500505

[121] Guzzetta G Kirschner D. The roles of immune memory and aging in protective immunity and endogenous reactivation of tuberculosis. PloS One. (2013) 8:e60425. doi: 10.1371/journal.pone.0060425 23580062 PMC3620273

[122] Kudryavtsev I Zinchenko Y Serebriakova M Akisheva T Rubinstein A Savchenko A. A key role of CD8+ T Cells in controlling of tuberculosis infection. Diagnostics. (2023) 13(18):2961. 37761328 10.3390/diagnostics13182961 PMC10528134

[123] Silva Miranda M Breiman A Allain S Deknuydt F Altare F. The tuberculous granuloma: an unsuccessful host defence mechanism providing a safety shelter for the bacteria? Clin Dev Immunol. (2012) 2012:1–14. doi: 10.1155/2012/139127 PMC3395138 22811737

[124] Gong C Linderman JJ Kirschner D. A population model capturing dynamics of tuberculosis granulomas predicts host infection outcomes. Math Biosci Eng. (2015) 12:625–42. doi: 10.3934/mbe.2015.12.625 PMC4447319 25811559

[125] Warsinske HC DiFazio RM Linderman JJ Flynn JL Kirschner DE. Identifying mechanisms driving formation of granuloma-associated fibrosis during Mycobacterium tuberculosis infection. J Theor Biol. (2017) 429:1–17. doi: 10.1016/j.jtbi.2017.06.017 28642013 PMC5576548

