# Corrigendum: Characterizing the immune response to *Mycobacterium tuberculosis*: a comprehensive narrative review and implications in disease relapse

**DOI:** 10.3389/fimmu.2025.1560113

**Published:** 2025-04-17

**Authors:** Fatima Rahman

**Affiliations:** ^1^ Department of Pharmacology, University College London, London, United Kingdom; ^2^ Istituto per le Applicazioni del Calcolo, Consiglio Nazionale delle Ricerche, Rome, Italy

**Keywords:** immune response, tuberculosis, adaptive immunity, innate immunity, granuloma, relapse

In the published article, there were errors in the text. The corrections are shown below.

A correction has been made to *Section 3*, last paragraph. The sentence previously stated: "…a tabular summary of the collated narrative data…”. The corrected sentence is “…a tabular summary of the collated data…”.

A correction has been made to *Section 5.1.4*, Paragraph 1. The sentence previously stated: “…reactive nitrogen species (NOS), and…”. The corrected sentence is: “…reactive nitrogen species (RNS), and…”.

A correction has been made to *Section 5.4.4,* Paragraph 1. The sentence previously stated: “(i.e., programmed cell death) (63).” The corrected sentence is: “(i.e., programmed cell death) (63), while granulysin may form pores and kill *Mtb* (62).”

A correction has been made to *Section 5.5.4.2,* last paragraph. The sentence previously stated: “Third, *Mtb* debris extrusion from apoptotic AMs can engage with resting AMs, …”. The corrected sentence is: “Third, *Mtb* debris extrusion from apoptotic AMs can engage with resting DCs, …”.

A correction has been made to *Section 6*, Paragraph 1. The sentence previously stated: “The adaptive immune response occurs in the DLN…”. The corrected sentence is: “The adaptive immune response generates in the DLN…”.

A correction has been made to *Section 8.3*, Paragraph 3. The sentence previously stated: “…from normal aerobic respiration to anaerobic respiration (2). As a result of glucose deficiency, macrophages shift to using lipids…”. The corrected sentence is: “…from normal aerobic respiration to alternative (anaerobic type) pathways (2). As a result of glucose deficiency, mycobacteria shift to using lipids…”.

A correction has been made to *Section 11.4.1,* Paragraph 1. The sentence previously stated: “Although cynomolgus macaques closely resemble various manifestations of human TB and immunopathology, there are few data regarding their ability to effectively model TBI, for instance (120).” The corrected sentence is: “Although cynomolgus macaques closely resemble various manifestations of human TB (120), no animal model can completely represent human disease (24).”

A correction has been made to **Table 1**, headers/Neutrophils/Natural killer cells. The headers previously stated: “Player” and “Narrative data extracted”. The corrected headers are: “Player/component” and “Data extracted”. The description previously stated: “…CD8+ T cells; NET production”. The corrected description is: “…CD8+ T cells”. The description previously stated: “…; types of antigen recognition”. The corrected description is: “…; role of granulysin”.

A correction has been made to **Table 2**, Th1. The sentence previously stated: “TNF-α ultimately induces apoptosis of infected macrophages (121) by Fas-Fas ligand (FasL) (72).” The corrected sentence is: “TNF-α induces apoptosis of infected macrophages (121); apoptosis can also occur by the Fas-FasL pathway (72).”

**Table 2:** T-cell effector functions by subset.

CD4+ T cell vs. CD8+ T-cell effector functions	
CD4+ T cell subset	
**Th1 cells**	In the lungs, Th1-type cytokines (IFN-γ, TNF-α, and IL-12) are central to protective immunity, as they activate macrophages to further antimicrobial activity (79). IFN-γ is produced mainly by Th1 cells; this occurs before and after activated macrophage response (72). IFN-γ activates macrophages, which is critical for intracellular bacterial elimination (a distinctive feature of *Mtb*) (66). Macrophages are the main target cells; however, following macrophage activation, they can kill intracellular bacteria and heighten the protective Th1 response (72) (**Figure 4D**). Activated and resident macrophages kill extracellular bacteria, while intracellular bacteria are only killed when the infected macrophage dies via cytolytic action or apoptosis (72). Infected macrophages secrete cytokines like TNF-α to recruit CD4+ and CD8+ T cells and activate their effector functions at the infection site (121). TNF-α induces apoptosis of infected macrophages (121); apoptosis can also occur by the Fas-FasL pathway (72). FasL is a membrane-bound molecule that is expressed by CD8+ and Th1 cells (63). The binding of the FasL to its receptor induces death by apoptosis in target cells (63). Following the apoptotic death of infected macrophages, neutrophils are attracted to the sites of mycobacterial release within the extracellular space (66). IL-12 is produced by macrophages in response to antigen stimulation. Its main function is to induce differentiation of Th0 lymphocytes into Th1 lymphocytes and enhance the production of IFN-γ (69). When macrophages are primed with IFN-γ, the production of IL-12 is greatly increased; however, the production can also be inhibited by IL-10 (69). Both Th1 and Th2 cells also produce IL-10 (72). IL-10 and other cytokines deactivate macrophages (72). IL-10 inhibits the production of cytokines and chemokines; this prevents cellular apoptosis and necrosis and alters the activation phenotype of macrophages (67). It dampens MHC II expression and NO production while circumventing the antimycobacterial effects of IFN-γ on macrophages (69).
**Th2 cells**	The Th2 response targets extracellular bacteria (i.e., humoral immunity) (72). Mature Th2 cells produce IL-4, IL-5, and IL-13 (77) and IL-10 (72). IL-4 promotes further Th2 cell differentiation in naïve T cells as they encounter antigens in a positive feedback loop (77). IL-4 and IL-13 reduce macrophage bacterial killing by dampening cellular responsiveness to IFN-γ and inhibiting iNOS production (65). IL-4 also mediates IgE class switching in B cells and IL-5 recruits eosinophils (77), which express the chemokine receptor CCR3—this enables eosinophils to respond to various inflammatory stimuli (52) (**Figure 4D**).
**Th17 cells**	Th17 effector cell function is governed by IL-23, which is characterized by the production of IL-17A, IL-17F, IL-21, and IL-22 (77). Th17 cells are thought to play an important role in controlling extracellular bacteria (77). The pro-inflammatory responses of IL-17A, IL-17F, and IL-22 include neutrophilia, tissue remodeling, and antimicrobial protein production (85). Meanwhile, IL-22 enhances phagolysosomal fusion, which inhibits *Mtb* intracellular growth (31) (**Figure 4D**). For further information on the roles of iTreg cells and B cells, see the **Supplementary Material** .
CD8+ T cell subset	
**Tc1 cells**	Functionally, Tc1 cells are characterized by their high levels of IFN-γ and TNF-α (88, 122). Resting macrophages are activated by IFN-γ, which enhances their pathogen-clearing ability and cytokine release (69). Cytotoxic CD8+ T cells also produce IFN-γ, which induces MHC class I expression to increase the chances for recognition and bacterial killing; IFN-γ activates macrophages for further bactericidal activity (i.e., phagocytic functions and antigen presentation) (63). IL-12 enhances the production of IFN-γ (69). T cells are killed by IFN-γ-induced apoptosis and natural cell death (based on their half-life) (34). Functions of TNF-α include recruitment of macrophages and T cells, activation of macrophages (with IFN-γ and bacterial signals), and induction of apoptosis of infected macrophages (34) (**Figure 4F**). Further, Tc1 cells produce high levels of perforin and granzyme B, thus demonstrating exceptional cytotoxic activity (88, 122). This bacterial killing process, cytolysis, involves the use of granules containing perforin, granzymes, and granulysin to destroy the infected cells (62). Similar to NK cells, the pore-forming protein, perforin, penetrates the target cell membrane, allowing granzyme entry to induce apoptosis (63). Granulysin has antimicrobial activity and is pro-apoptotic (63)(**Figure 4F**).
**Tc2 cells**	Tc2 cells are known for their production of type II cytokines, such as IL-4, IL-5, and IL-13 (88, 122). IL-4 and IL-13 promote immune suppression (65, 122). IL-4 and IL-13 reduce iNOS synthesis and cellular responsiveness to IFN-γ (65). IL-5 recruits eosinophils (77). These type II cytokines promote B-cell class switching for IgE production (77). Tc2 cells also express high levels of granzyme B, possessing cytotoxic abilities similar to Tc1 cells (88) (**Figure 4F**). Following bacterial elimination, the T-cell response is concluded via antigen removal, thereby restricting T-cell activation and abrogating the recruitment of new effector T cells (77).
**Tc9, Tc17, Tc22, and CD8 memory T cells**	The effector functions of Tc9, Tc17, Tc22, and CD8 memory T cells can be found in the **Supplementary Material**.

In the published article, there was an error in **Figure 4A** as published. The MHC I presentation icons have been deleted as this applies to CD8+ T cells. The corrected **Figure 4A** appears below.

**Figure 4:**
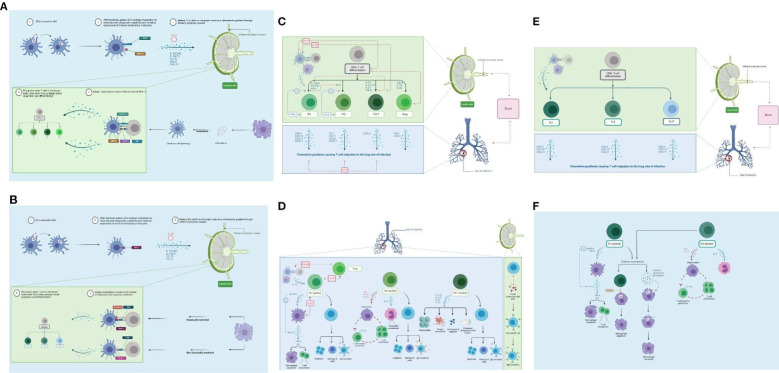
Adaptive immune response. **(A)** DC maturation and migration from the lungs (blue) and presentation to naïve CD4+ T cells in the lymph nodes (green). As maturation occurs, DCs migrate through the afferent lymphatic vessels and enter the T-cell area of the DLN. This migration is influenced by IL-12 release and chemokines. IL-10 can block this movement. DCs provide two primary actions within the DLN: naïve T-cell recruitment and antigen presentation. **(B)** DC maturation and migration from the lungs (blue) and presentation to naïve CD8+ T cells in the lymph nodes (green). CD8+ T cells can detect *Mtb* antigens (as peptides) presented by both classical and non-classical MHC molecules. **(C)** CD4+ T-cell differentiation in the lymph node (green) and migration to lungs (blue). Antigen-specific naïve CD4+ T cells undergo clonal expansion and effector differentiation. CD4+ T cells sense cytokines, which activate differentiation programs that result in their polarization toward specialized T-helper cell subsets. **(D)** CD4+ T-cell effector functions in the lungs (blue) and lymph nodes (green). Th1 cells produce IFN-γ to activate macrophages (eliminates intracellular *Mtb*). The Th2 response targets extracellular bacteria (i.e., humoral immunity). Th17 effector function is driven by IL-23; production of IL-17A, IL-17F, IL-21, and IL-22 to control extracellular bacteria. **(E)** CD8+ T-cell differentiation in the lymph node (green) and migration to the lung (blue). Following antigen presentation, CD8+ T cells can differentiate into Tc1, Tc2, and Tc17 cells. **(F)** CD8+ T-cell effector functions in the lungs (blue). Tc1 cells produce high levels of IFN-γ and TNF-α. Tc2 cells produce type II cytokines, which promote immune suppression. DC, dendritic cell; DLN, draining lymph node. Created with BioRender.com.

In the published article, there was an error in **Figure 6** as published. “Anaerobic” label changed to “Decreased oxygen levels (e.g. granuloma)” for clarity; “NOS” changed to “NO”/”RNS”; lipid icon changed to cholesterol. The corrected **Figure 6** appears below.

**Figure 6:**
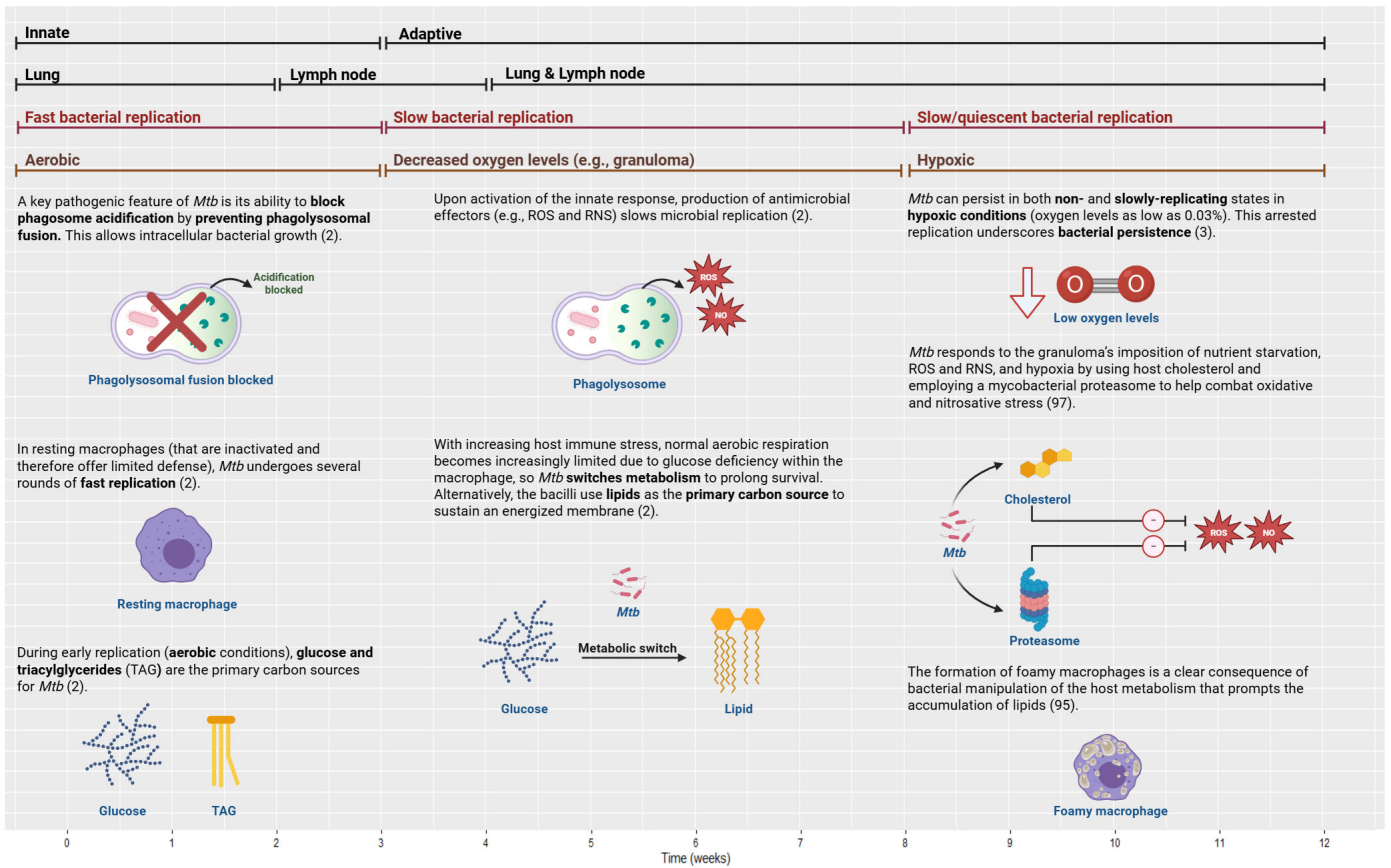
Bacterial growth and manipulation in TB over time from innate to adaptive (first bars), with the associated compartments (second bars), bacterial replication rates (third bars), and oxygen level/conditions (fourth bars). To establish persistence, *Mtb* employs several effector mechanisms within the host cells and the granuloma. TB, tuberculosis; NO, nitric oxide; RNS, reactive nitrogen species; ROS, reactive oxygen species. Created with BioRender.com

In the published article, there was an error in **Supplementary Tables 1-3**: “NOS” changed to “NO”; section citations/cross-references changed to inline citations for clarity. Table 3: (IL-5) changed “Immune suppression” to “Recruits eosinophils”; (TGF-β) changed “Chemotactic gradient to recruit immune cells” to “Anti-inflammatory effects”; (PGE2) changed “Downregulates production in phagocytosing macrophages to diminish the anti-inflammatory effect” to “Downregulates pro-inflammatory cytokine production in phagocytosing macrophages”. Minor formatting changes made. The correct supplementary material has been updated in the original article.

The author apologizes for these errors and states that this does not change the scientific conclusions of the article in any way. The original article has been updated.

