# Supplementary material for: Characterizing the immune response to Mycobacterium tuberculosis: a comprehensive narrative review and implications in disease relapse
Source: Front Immunol. 2024 Nov 22;15:1437901. doi: 10.3389/fimmu.2024.1437901 (PMC11620876; doi:10.3389/fimmu.2024.1437901)
Supplement: Supplementary file 1 [file DataSheet1.pdf]

Supplementary Data for Immune Response to Mycobacterium tuberculosis

1 Treatment of drug-susceptible TB: challenges

For drug-susceptible TB, the lengthy 6-month treatment duration is a considerable burden on patients and health services (1), elevating the risk of patient non-compliance, drug resistance (2), and toxicity (3). Hence, the shortening of treatment duration is a major milestone in antitubercular drug development (4).

2 Drug resistance in TB

The emergence of resistance is an inevitable consequence of drug use; notably when pathogens are exposed to low, inefficacious doses over time by inducing bacterial mutations (5). To further elucidate TB drug resistance, there are distinct categories: isoniazid-resistant TB which is caused by *Mtb* strains that are resistant to isoniazid (6); rifampicin-resistant TB (RR-TB) which is caused by *Mtb* strains that are resistant to RIF; multi-drug-resistant TB (MDR-TB) which is caused by *Mtb* strains that are resistant to at least both RIF and INH; extensively drug-resistant TB (XDR-TB) which is caused by *Mtb* strains that fulfill the definition of MDR-TB/RR-TB and are also resistant to any fluoroquinolone (i.e., levofloxacin and moxifloxacin) and at least one additional group A drug (currently, these include levofloxacin or moxifloxacin, bedaquiline, and linezolid); and finally, pre-XDR-TB which is caused by *Mtb* strains that fulfill the definition of MDR-TB/RR-TB and are also resistant to any fluoroquinolone (7). That is, the development of resistance in *Mtb* is complex (5). It involves the interaction of clinical, biological, and microbiological processes, such as non-adherence of patients to therapy, which leads to the development of genetic resistance (5). Further, the complexity of granulomas hinders effective drug distribution (5).

3 The immune response and drug development: challenges

The immune response has direct implications in the drug development arena. For instance, due to the immunoevasive mechanisms employed by *Mtb*, the bacteria can survive in diverse microenvironments as it migrates from healthy lung tissue into lesions, notably in physiologically distinct subpopulations (8). These include rapidly replicating bacteria (aerobic conditions), slowly replicating bacteria (anaerobic conditions), and non-replicating bacteria (hypoxic conditions); and resistant and persistent states. As such, drugs targeting *Mtb* need to be active in different phases of infection (8). Further complications drug development is the adaptable cell wall of *Mtb* itself; this macromolecular structure serves to protect the mycobacterium from hydrophilic compounds. This explain why novel treatment strategies focus on targeting the mycobacterial cell wall components (8).

Hence, to achieve cure, TB treatment must contain drugs that achieve all of the following criteria: kill rapidly replicating *Mtb* to swiftly reduce the bacterial load (isoniazid); eliminate slowly replicating bacteria that cause relapse (rifampicin and pyrazinamide), and shield the combination drugs against the development of resistance (ethambutol) (9). However, TB control efforts are hindered by the complex and long-duration treatment regimens required for cure without relapse (9). In addition, drugs differ in their ability to penetrate the various

lesions found in human pulmonary TB disease (9). This is due to the spatial heterogeneity of the granuloma, which forms a barrier to drug penetration (10). Bacteria also develop phenotypic tolerance to antibiotics inside granulomas, thereby further impeding drug development (10).

4 Bacterial phenotypes

The Multistate Tuberculosis Pharmacometric model quantifies bacterial growth by use of three bacterial phenotypes: rapidly replicating, slowly replicating, and non-replicating (11).

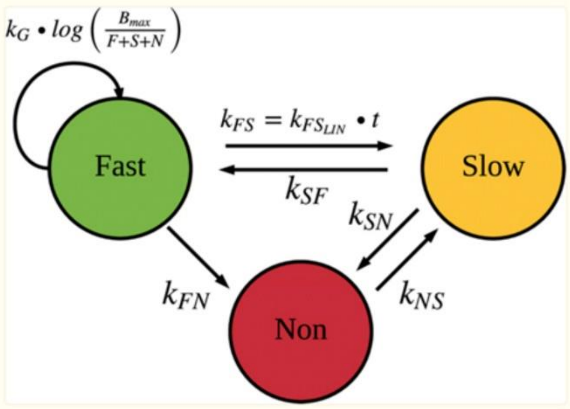

Fig. 1

Schematic illustration of the Multistate Tuberculosis Pharmacometric model. F, fast-multiplying bacterial state; S, slow-multiplying bacterial state; N, non-multiplying bacterial state; k_G, growth rate of the fast-multiplying state bacteria; k_{FS}, time-dependent linear rate parameter describing transfer from fast- to slow-multiplying bacterial state; k_{SF}, first-order transfer rate between slow- and fast-multiplying bacterial state; k_{FN}, first-order transfer rate between fast- and non-multiplying bacterial state; k_{SN}, first-order transfer rate between slow- and non-multiplying bacterial state; k_{NS}, first-order transfer rate between non-multiplying and slow-multiplying bacterial state

From Clewe et al. (11)

Supplementary Figure 1: Schematic representation of the Multistate Tuberculosis Pharmacometric model to illustrate the bacterial phenotypes in TB. From: Clewe et al. (11). Licensed under a Creative Commons Attribution (CC-BY) license. Disclaimer:
No changes were made in the re-use of this material. Link to the original material. Copyright © 2020 The Author(s). Created with BioRender.com.

5 Legends for figures

Definitions of the icons used to represent each cellular player (**Supplementary Figure 2**), including the colors and arrows in the figures (**Supplementary Figure 3**) are provided.

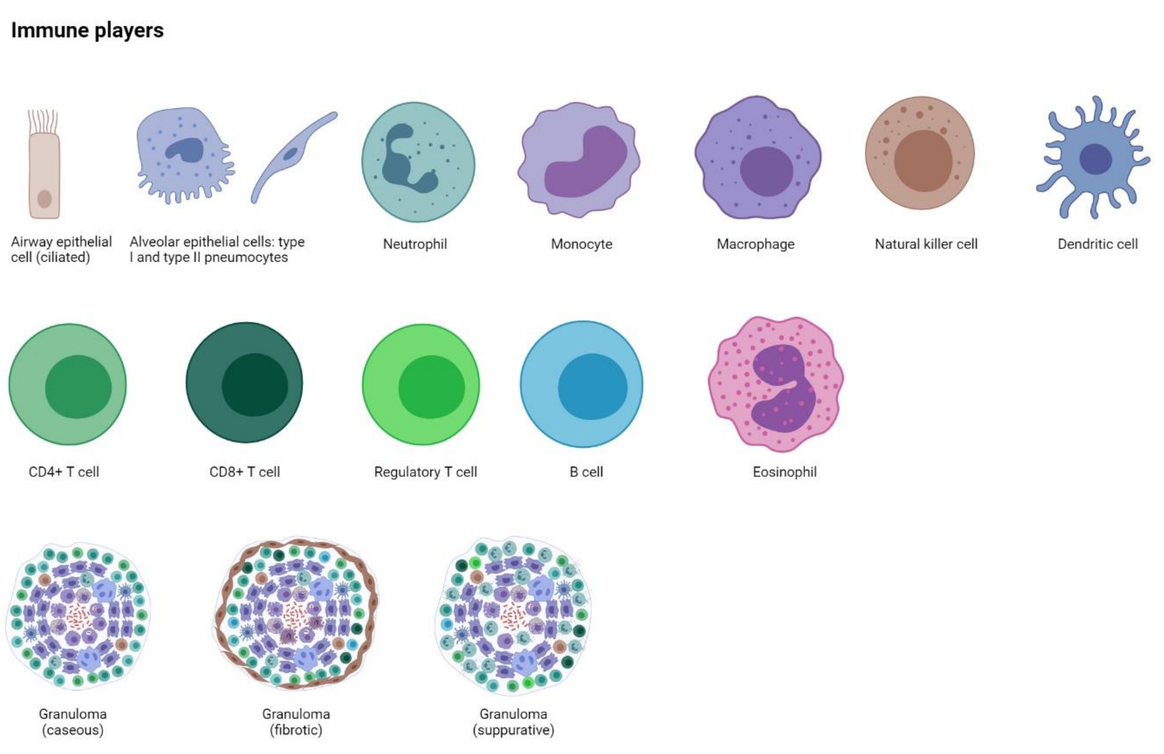

Supplementary Figure 2: Legend of icons used per cell type in the immune response to TB. Created with BioRender.com.

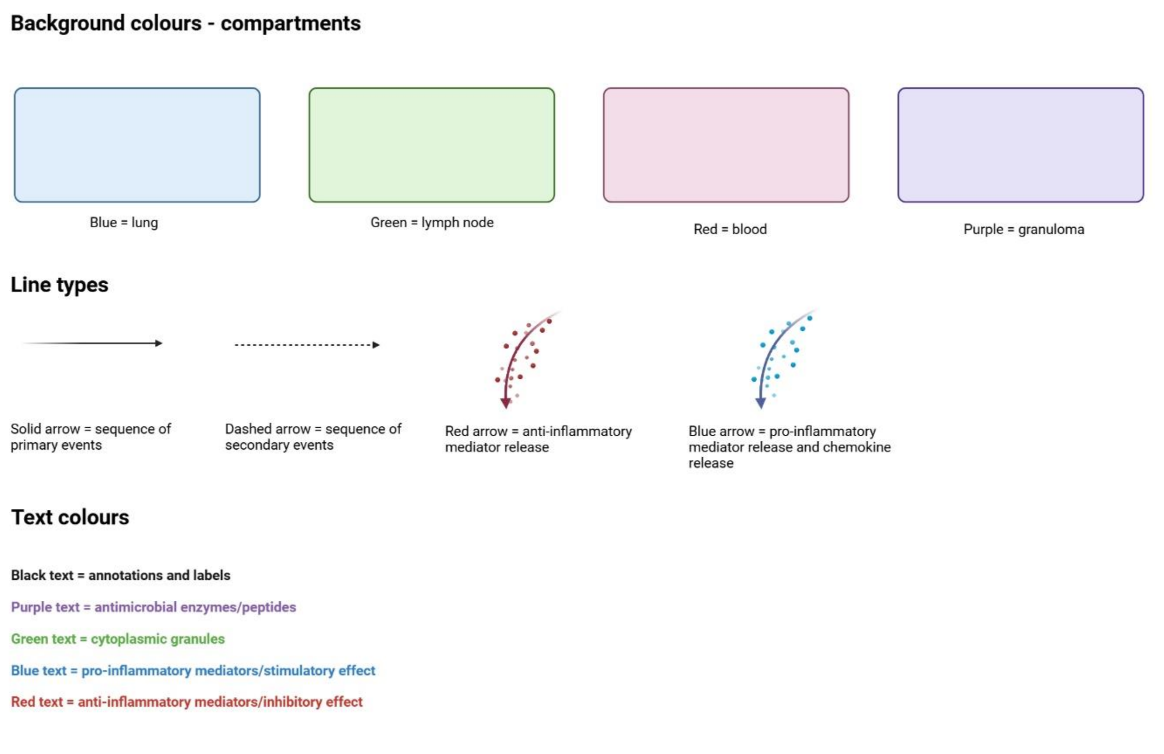

Supplementary Figure 3: Legend of the background colors, line types, and text colors to indicate different compartments, sequence of primary versus secondary events, mediator release, and types of antimicrobial effector molecules. Created with BioRender.com

6 High-level summary of immune response – innate and adaptive phases

A high-level summary of each player in the innate and adaptive response is presented in **Supplementary Figure 3** and **Supplementary Figure 4**, respectively.

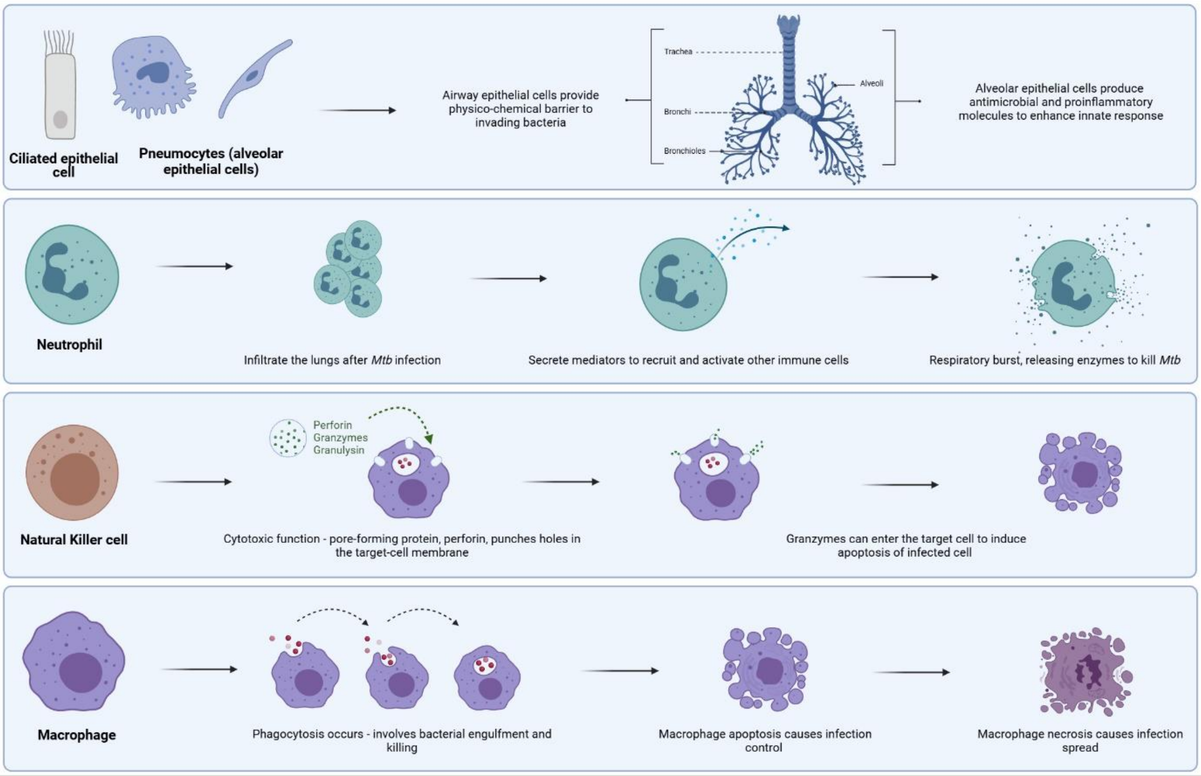

Supplementary Figure 4: Summary of the innate immune response per player. Created with BioRender.com.

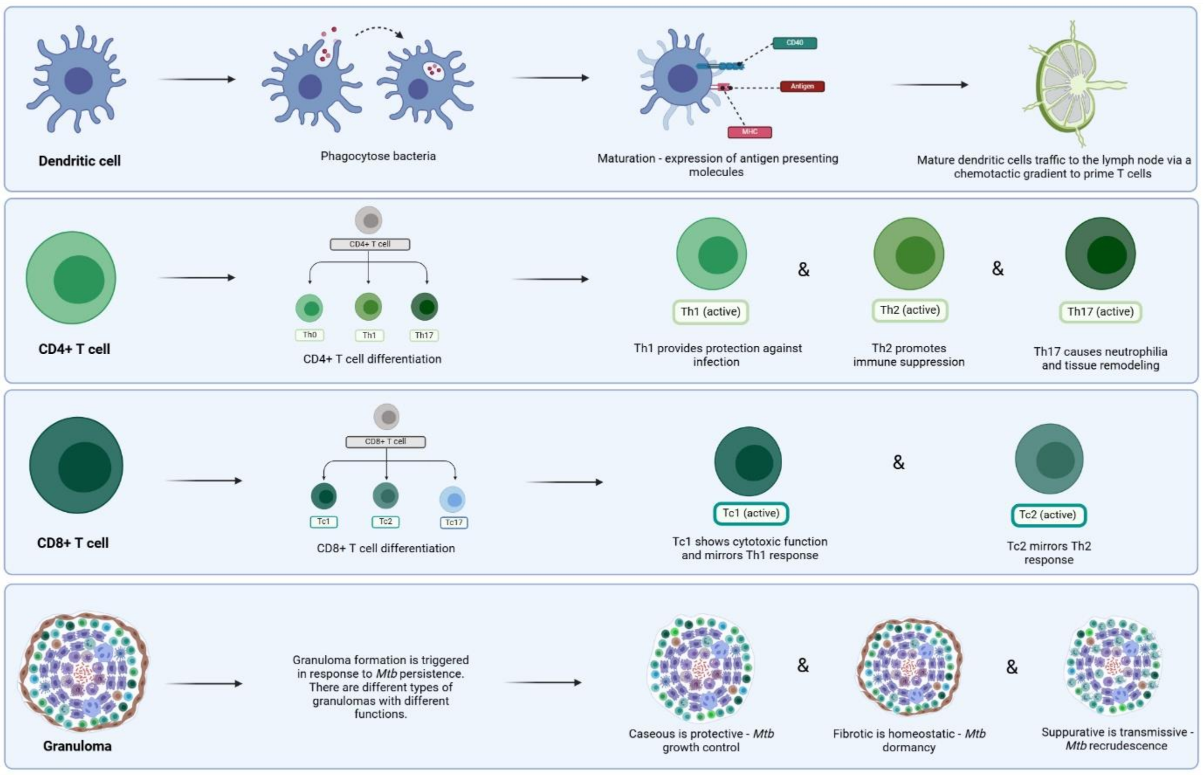

Supplementary Figure 5: Summary of the adaptive immune response per player, and granuloma response. Created with BioRender.com.

7 Innate immune response

7.1 Airway epithelial cells

7.1.1 Goblet cells

Goblet cells and mucous cells originating from submucosal glands perpetually generate airway mucus that envelops the airway epithelium (12). They also cooperate with the rhythmic beats of cilia on ciliated cells to allow effective airway clearance via the mucociliary escalator, which can expel almost 90% of inhaled foreign microorganisms (12). Clara cells, neuroendocrine cells, and basal cells also aid in infection control (e.g., neuroendocrine regulation by neuroendocrine cells) (12).

7.1.2 Airway mucus

Airway mucus is considered a viscoelastic gel containing an glycosylated mucins and multiple anti-mycobacterial components, such as defensins, immunoglobulins, lysozymes, and cytokines (12). Plasma cells produce immunoglobulin A (IgA) antibodies for diverse *Mtb* surface antigens that can block *Mtb* infection of lung epithelial cells (12). Secretory IgA covers mucosal surfaces to hinder the attachment of pathogens and neutralize their toxins (12).

7.1.3 Innate lymphoid cells (ILCs)

Moreover, ILCs are subsets of lymphocytes at mucosal surfaces that act early during *Mtb* infection (12,13). ILCs are categorized into five groups: natural killer (NK) cells, ILC1s, ILC2s, ILC3s, and lymphoid tissue-inducer (LTi) cells (12). ILCs lack rearranged antigen-specific

receptors; therefore, they are not MHC-restricted and functionally mirror adaptive lymphocytes (12). ILC1s, ILC2s, and ILC3s mirror the corresponding T-helper subsets including Th1, Th2, and Th17, respectively (12).

7.1.4 Innate immune T cells

The major groups of innate immune T cells are include mucosal-associated invariant T (MAIT) cells, natural killer T (NKT) cells, and gamma delta (γδ) T cells, depending on their antigen presentation process (12). MAIT cells are innate-like CD8+ T cells; that is, they recognize pathogens via MHC-I-related molecules (i.e., MR1 molecules) with limited polymorphisms (14). They can respond to *Mtb*-infected lung epithelial cells by producing IFN-γ, TNF-α, and granzymes (12). MAIT cells are thought to be involved in the innate response to intracellular pathogens, for which they produce cytokines such as IFN-γ, TNF-α, and IL-17 (14). MAITs also have cytotoxic capabilities as they produce granzyme B (14). Although the role of MAITs in the immune response in TB is unclear, these cells may play an important role in early host defense within the airways (14).

Moreover, NKT cells are characterized by their specificity for lipid antigens, which are presented by MHC class I-like CD1d molecules. NKT cells are further categorized into type I (invariant) and type II (diverse) subsets according to their different T cell receptor (TCR) expressions (12). In TB, NKT cells release IL-21 to aid immunoglobulin production by B cells (12). Recent studies have not addressed the immunological role of type II NKTs in TB (12).

In addition, γδ T cells usually lack CD4 or CD8 T cell lineage markers but they are still able to identify numerous cellular ligands via both TCR-dependent and TCR-independent pathways due to their expression of various pattern recognition receptors (12). Notably, circulating levels of γδ T cells are altered in TB patients to offer protective mechanisms. For instance, they produce inflammatory cytokines such as IFN-γ, TNF-α, and IL-17, which may also influence granuloma formation (12). Moreover, γδ T cells promote the maturation of DCs infected with mycobacteria, thereby indirectly protecting the host from mycobacterial infection (12).

8 Adaptive immune response

8.1 CD4+ T cells – effector functions

8.1.1 iTreg cells

Based on their origin, natural (constitutive) regulatory T cells (nTreg) are distinguished from inducible (adaptive) CD4+ CD25+ Foxp3+ regulatory T cells (iTreg). These two cell types have overlapping roles in immune response regulation (15). The nTreg cells are derived from the thymus and undergo proliferation in peripheral organs, where they can inhibit the activation of effector cells (15). iTreg cells that originate from CD4+ T cells in the peripheral regions frequently exhibit comparable characteristics and perform similar functions to nTreg cells. However, they may employ distinct mechanisms of immune regulation (15). The iTreg and nTreg cells work synergistically to promote immune tolerance (16).

The iTreg cells comprise various cellular subtypes; for instance, IL-10-producing Tr1 cells, TGF-β-producing Th3 cells, and FOXP3+ inducible T cells (15). IL-10 is an essential

immunosuppressant cytokine generated by regulatory T cells (17). TGF-β is also produced by regulatory T cells (17). Although the importance of IL-10 and TGF-β as suppressive mediators is well established, their contribution to nTreg cell function remains debatable (15). Type I IFNs affect Treg by decreasing their suppressive capabilities and allowing for greater CD4+ T cell activation (18). During chronic infection, type I IFNs induce an immunosuppressive environment by boosting the expression of programmed death-ligand 1 (PD-L1) and IL-10 by DCs. These immunoregulatory DCs cause CD4+ T cell exhaustion (18). In TB specifically, Treg suppresses the Th1 population effector functions (19); it also negatively modules the differentiation of CD4+ T cell subsets (19).

8.1.2 B cells

Antibody-independent B cell capacities, such as the ability to produce type I IFNs or anti-*Mtb* antibodies that inhibit bacterial survival inside macrophages, suggest that lung-resident B cells may affect early events in TB (20). B cells interact with the Th1 cell population, prompting three distinct processes to occur simultaneously: B cells proliferate, mature into memory B cells, and produce IgG after a successful encounter (19). Similarly, Th2 cell interaction with B cells results in B cell duplication, differentiation into memory B cells, and IgA secretion (19). Th17 cell migration and interaction with B cells results in cell duplication and IgE production (19). After interacting with *Mtb* inside the DLN, B cells become activated and release IgM (19).

8.2 CD8+ T cells – effector functions

8.2.1 Tc9 cells

In various diseases, it is thought that Tc9 cells produce IL-9 but a small amount of IFN-γ (21). They have low cytotoxic activity, partly due to their reduced granzyme B production (21). However, their role in TB has yet to be examined.

8.2.2 Tc17 cells

In TB, Tc17 cells are characterized by the production of IL-17A, IL-17F, and IL-22 (22). In diseases that are caused by intracellular pathogens, inflammatory responses such as neutrophilia, tissue remodeling, and antimicrobial protein production are induced by IL-17A, IL-17F, and IL-22 (16). [In the innate response, it has been shown that IL-22 inhibits *Mtb* intracellular proliferation by promoting phagolysosomal fusion (23).] Similarly to Tc9 cells, Tc17 cells also express limited granzyme B and demonstrate poor cytolytic activity (21). Evidence suggests that Tc17 can also develop into memory T cells with a long lifespan (21). In TB specifically, one study showed increased levels of IL-17 (derived from Tc17 cells) in infected patients, which may indicate deleterious effects of this cell type (24).

8.2.3 Tc22 cells

In various diseases, Tc22 cells primarily produce IL-2, IL-22, and TNF-α (21). Functionally, Tc22s exhibit high cytolytic activity and are characterized by the expression of granzyme B (21). While bearing resemblance to Th22 cells, the categorization of Tc22 cells as a distinct T cell lineage is debatable, particularly because of the ability of Th17 cells to also produce IL-22 (21). Notably, their role in TB has yet to be examined.

8.2.4 Memory CD8+ T cells

In diseases caused by intracellular pathogens (for instance), when an antigen is re-encountered in the periphery, effector CD8+ T cells from various subpopulations can fulfill their distinct functions. Following the primary response, the majority of CD8+ T cells undergo apoptosis; however, a small fraction persists as long-lived memory T cells. These memory CD8+ T cells exhibit robust proliferation and rapidly transform into effector cells upon re-exposure to the specific antigen (25). It is noteworthy, however, that whilst the role of CD4+ T cells is well documented in TB, that of CD8+ T cells has yet to be established definitively in humans (14).

9 Summary of immune players

Supplementary Table 1: Summary of cellular players, mediators, effectors, and site of action

Player	Mediators/molecules produced	Type of molecule	Site
**Airway epithelium**			
**Airway epithelial cells**	TNF-α (13,26)	Pro-inflammatory cytokine	Airways
	IFN-γ (13,26)	Pro-inflammatory cytokine	Airways
	GM-CSF (13,26)	Pro-inflammatory cytokine	Airways
	IL-6 (13,26)	Pro-inflammatory cytokine	Airways
	IL-10 (13)	Anti-inflammatory cytokine	Airways
	IL-8 (13,26)	Chemokine	Airways
	IP-10 (13,26)	Chemokine	Airways
	IL-27 (13,26)	Chemokine	Airways
	MCP-1 (13,26)	Chemokine	Airways
	MIG (13)	Chemokine	Airways
	LL-37 (13)	Antimicrobial peptide	Airways
	β-defensin-2 (13)	Antimicrobial peptide	Airways
	Hepcidin (13)	Antimicrobial peptide	Airways
	Gro-α (CXCL1) (27)	Chemokine	Airways
	ENA-78 (CXCL5) (27)	Chemokine	Airways
	ROS (13)	Highly reactive molecule	Airways
	RNS (13)	Highly reactive molecule	Airways
**Goblet cells**	Defensins (12)	Antimicrobial peptide	Airways
	Immunoglobulins (12)	Immunoglobulin	Airways
	Lysozymes (12)	Antimicrobial enzyme	Airways
**Plasma cells**	IgA antibodies (12)	Immunoglobulin	Airways
**MAIT cells**	IFN-γ (14)	Pro-inflammatory cytokine	Airways
	TNF-α (14)	Pro-inflammatory cytokine	Airways
	IL-17 (14)	Pro-inflammatory cytokine	Airways
	Granzymes (14)	Antimicrobial enzyme	Airways
**NKT cells**	IL-21 (12)	Pro-inflammatory cytokine	Airways
**γδ T cells**	IFN-γ (12)	Pro-inflammatory cytokine	Airways

Player	Mediators/molecules produced	Type of molecule	Site
	TNF-α (12)	Pro-inflammatory cytokine	Airways
	IL-17 (12)	Pro-inflammatory cytokine	Airways
	IL-8 (27)	Chemokine	Airways
**Alveolar epithelium**			
**Type I and II pneumocytes**	Immunoglobulins (12)	Immunoglobulin	Lungs
	Antimicrobial peptides (12)	Peptides	Lungs
	Surfactant proteins (12)	Proteins	Lungs
	TGF-β (13)	Anti-inflammatory cytokine	Lungs
**Type II pneumocytes**	CXCL5 (20)	Chemokine	Lungs
	Hydrolases (12)	Antimicrobial enzyme	Lungs
**Neutrophils**			
	IL-1 (19)	Pro-inflammatory cytokine	Lungs
	IFN-γ (13)	Po-inflammatory cytokine	Lungs
	TNF-α (13,28)	Po-inflammatory cytokine	Lungs
	IL- 1β (28)	Po-inflammatory cytokine	Lungs
	IL-12 (28)	Po-inflammatory cytokine	Lungs
	VEGF (28)	Pro-inflammatory protein	Lungs
	IP-10 (13)	Chemokine	Lungs
	MCP-1 (13)	Chemokine	Lungs
	MIP-1α/β (13)	Chemokine	Lungs
	α-defensins (13)	Antimicrobial peptide	Lungs
	Matrix metalloproteases (13)	Antimicrobial enzyme	Lungs
	Arginase (23)	Antimicrobial enzyme	Lungs
	Lactoferin (13)	Antimicrobial glycoprotein	Lungs
	Lipocalin 2 (29)	Antimicrobial protein	Lungs
	Gelatinase B (23)	Antimicrobial enzyme	Lungs
	Elastase (23)	Antimicrobial enzyme	Lungs
	Collagenase (23)	Antimicrobial enzyme	Lungs
	Myeloperoxidase (23)	Antimicrobial enzyme	Lungs
	NETs (30)	Antimicrobial molecules	Lungs

Player	Mediators/molecules produced	Type of molecule	Site
**NK cells**			
	IFN-γ (23)	Pro-inflammatory cytokine	Lungs
	IL-22 (23)	Pro-inflammatory cytokine	Lungs
	Perforin (31)	Antimicrobial protein	Lungs
	Granulysin (31)	Antimicrobial protein	Lungs
	TNF-α (13)	Pro-inflammatory cytokine	Lungs
**Macrophages**			
**M1**	TNF-α (12)	Pro-inflammatory cytokine	Lungs
	GM-CSF (12)	Pro-inflammatory cytokine	Lungs
	IL-12 (19)	Pro-inflammatory cytokine	Lungs
	IL-1β (12)	Pro-inflammatory cytokine	Lungs
	IL-6 (12)	Pro-inflammatory cytokine	Lungs
	IL-23 (12)	Pro-inflammatory cytokine	Lungs
	G-CSF (27)	Pro-inflammatory cytokine	Lungs
	IL-8 (32)	Chemokine	Lungs
	MIP-2 (32)	Chemokine	Lungs
	IP-10 (32)	Chemokine	Lungs
	MCP-1 (32)	Chemokine	Lungs
	LL-37 (27)	Antimicrobial peptide	Lungs
	LTB4 (27)	Leukotriene	Lungs
	Gro-α (CXCL1) (27)	Chemokine	Lungs
	IFN-α/β (type I interferons) (13)	Pro-inflammatory cytokine	Lungs
	ROS (33)	Highly reactive molecule	Lungs
	NO (33)	Highly reactive molecule	Lungs
**M2**	IL-10 (12)	Anti-inflammatory cytokine	Lungs
	TGF-β (12)	Anti-inflammatory cytokine	Lungs
	PGE2 (34)	Eicosanoid	Lungs
	ROS (33)	Highly reactive molecule	Lungs
	NO (33)	Highly reactive molecule	Lungs
**DCs**			
	IL-10 (35)	Anti-inflammatory cytokine	Lymph nodes
	IL-12 (19)	Pro-inflammatory cytokine	Lymph nodes

Player	Mediators/molecules produced	Type of molecule	Site
	IFN-α/β (type I interferons) (18)	Pro-inflammatory cytokine	Lymph nodes
	G-CSF (27)	Pro-inflammatory cytokine	Lymph nodes
	GM-CSF (27)	Pro-inflammatory cytokine	Lymph nodes
	LL-37 (27)	Antimicrobial peptide	Lymph nodes
	LTB4 (27)	Leukotriene	Lymph nodes
	Gro-α (CXCL5) (27)	Chemokine	Lymph nodes
	IL-6 (36)	Pro-inflammatory cytokine	Lymph nodes
	IL-23 (36)	Pro-inflammatory cytokine	Lymph nodes
	TGF-β (16)	Anti-inflammatory cytokine	Lymph nodes
	IL-1β (36)	Pro-inflammatory cytokine	Lymph nodes
	IL-27 (16)	Pro-inflammatory cytokine	Lymph nodes
	IL-35 (37)	Anti-inflammatory cytokine	Lymph nodes
	CCL18 (38)	Chemokine	Lymph nodes
	IL-8 (27)	Chemokine	Lymph nodes
	MIP-2 (27)	Chemokine	Lymph nodes
**CD4+ T cells**			
**Th1**	IFN-γ (39,40)	Pro-inflammatory cytokine	Lungs
	TNF-α (40)	Pro-inflammatory cytokine	Lungs
	IL-10 (39)	Anti-inflammatory cytokine	Lungs
**Th2**	IL-4 (41)	Anti-inflammatory cytokine	Lungs
	IL-5 (41)	Pro-inflammatory cytokine	Lungs
	IL-10 (39)	Anti-inflammatory cytokine	Lungs
	IL-13 (41)	Anti-inflammatory cytokine	Lungs
**Th17**	IL-17A (41)	Pro-inflammatory cytokine	Lungs
	IL-17F (41)	Pro-inflammatory cytokine	Lungs
	IL-21 (41)	Pro-inflammatory cytokine	Lungs
	IL-22 (41)	Pro-inflammatory cytokine	Lungs
**iTreg**	IL-10 (15)	Anti-inflammatory cytokine	Lungs
	TGF-β (15)	Anti-inflammatory cytokine	Lungs
	IL-35 (37)	Anti-inflammatory cytokine	Lungs
**CD8+ T cells**			
**Tc1**	IFN-γ (21,24)	Pro-inflammatory cytokine	Lungs

Player	Mediators/molecules produced	Type of molecule	Site
	TNF-α (21,24)	Pro-inflammatory cytokine	Lungs
	Perforin (21,24)	Antimicrobial protein	Lungs
	Granzyme B (21,24)	Antimicrobial enzyme	Lungs
	Granulysin (14)	Antimicrobial protein	Lungs
**Tc2**	IL-4 (21,24)	Anti-inflammatory cytokine	Lungs
	IL-5 (21,24)	Pro-inflammatory cytokine	Lungs
	IL-13 (21,24)	Anti-inflammatory cytokine	Lungs
**B cells**			
	IgG (19)	Immunoglobulin	Lungs
	IgA (19)	Immunoglobulin	Lungs
	IgE (19)	Immunoglobulin	Lungs
	IgM (19)	Immunoglobulin	Lymph nodes

CCL = CC chemokine ligand; CXCL = CXC chemokine ligand; DCs = dendritic cells; Ig = immunoglobulin; IFN = interferon; IL = interleukin; ILC = innate lymphoid cells; IP = interferon gamma-induced protein; iTreg = induced regulatory T cell; MAIT = mucosal-associated invariant T cells; MCP = monocyte chemoattractant protein; MIG = monokine induced by gamma interferon; MIP = macrophage inflammatory protein; NO = nitric oxide; RNS = reactive nitrogen species; PGE 2 = prostaglandin E 2; ROS = reactive oxygen species; Th = T helper cell; Tc = cytotoxic T cell; NETs = neutrophil extracellular traps; NK = natural killer; NKT = natural killer T cells; TGF = tumor growth factor; VEGF = vascular endothelial growth factor.

10 Ranking the immune players and mediators by importance

Supplementary Table 2 provides a ranking of the key immune cellular players as follows: macrophages, dendritic cells, CD4+ T cells, CD8+ T cells, neutrophils, airway/alveolar epithelial cells, and natural killer cells. The ranking from highest to lowest importance is based on the degree of involvement of each player throughout the innate, adaptive, and granuloma responses; and the influence each player has on disease outcome (i.e., significance and consistency in the research).

Supplementary Table 3 shows the ranking of the associated mediators of each cellular player based on the qualitative data collated (using the same ranking method).

Supplementary Table 2: Summary of the roles and significance ranking of cellular players in human TB

Player	Involvement in immune defense arms (innate, adaptive, granuloma)					
	Innate response	Adaptive response	Granuloma	Influence on disease outcome	Site	Importance in immune response to *Mtb* (High/Fair/Low)
**Airway epithelium**						
**Airway epithelial cells**	Yes	Undetermined<br><br>(may play a critical role in initiating protective adaptive immunity to mycobacterial infections - antigen presentation (13))	No	Express MHC I molecules and can directly present intracellular antigens to resident CD8+ T cells (13).<br>Play a role in initiating protective adaptive immunity (13).	Airways	High
**Goblet cells**	Yes	No	No	Can eliminate almost 90% of inhaled foreign particles and micro-organisms (12).	Airways	Fair
**Plasma cells**	Yes	No	No	Can prevent *Mtb* infection in the earliest stage of infection (12).	Airways	Low
**ILCs**	Yes	No	No	ILCs are thought to respond to various neurotransmitters that determine efficiency of	Airways	Low

Player	Involvement in immune defense arms (innate, adaptive, granuloma)			Influence on disease outcome	Site	Importance in immune response to *Mtb* (High/Fair/Low)
	Innate response	Adaptive response	Granuloma			
				the mucosal immune barrier (12), which may impact disease outcome.		
**MAIT cells**	Yes	No	No	Contribution to bacterial clearance is unclear; however, these cells may play an important role in the early host response in the airways (14).	Airways	Low
**NKT cells**	Yes	No	No	Much is unknown; however, iNKT cells from TB patients have a role in bacterial replication restriction (12).	Airways	Low
**γδ T cells**	Yes	Undetermined<br><br>(may indirectly help to control *Mtb* infection by promoting the maturation of DCs (12))	No	Inflammatory cytokine production and possible role in granuloma formation, displaying protective properties (12).<br>Promote the maturation of DCs infected with mycobacteria, thus indirectly aiding infection control (12).<br>γδ T cells are thought to demonstrate both innate and adaptive functions in response to *Mtb* (14), so they are believed to contribute to disease control.	Airways	Fair

Player	Involvement in immune defense arms (innate, adaptive, granuloma)<br>Innate response	 Adaptive response	 Granuloma	Influence on disease outcome	Site	Importance in immune response to *Mtb* (High/Fair/Low)
**Alveolar epithelium**						
**Type I pneumocytes**	Yes	No	No	Type I pneumocytes produce various effector molecules that enhance the bactericidal effects of macrophages (12). This early response is crucial to reduce disease burden.	Lungs	High
**Type II pneumocytes**	Yes	No	No	Type II pneumocytes produce various effector molecules such as lung hydrolases, which alone reduce 60% - 80% of bacterial cell adhesion and survival in human macrophages (12). This has a significant impact on disease outcome.	Lungs	High
**Macrophages**						
**M1 and M2**	Yes<br><br>(phagocytosis (42))	Yes<br><br>(antigen presentation (19))	Yes	Macrophage phagocytic and bacterial eradication capabilities evident throughout the immune response (32,42).<br>The macrophage initiates granuloma formation (43) and is the key cell type in most granulomatous lesions (44). Macrophages are imperative to disease control and outcome.	Lungs	High

Player	Involvement in immune defense arms (innate, adaptive, granuloma)			Influence on disease outcome	Site	Importance in immune response to *Mtb* (High/Fair/Low)
	Innate response	Adaptive response	Granuloma			
**Neutrophils**						
	Yes<br><br>(internalization via direct recognition or opsonization (27))	Yes<br><br>(in response to cell recruitment signals by activated macrophages (32))	Yes	Play a very complex role against *Mtb*, contributing to both bacterial eradication and dissemination (via necrosis) (23).<br>Suppurative granulomas (or necrotic neutrophilic granulomas) (45) are heavily infiltrated by neutrophils (28) which causes dissemination at later stages (46). Neutrophils are key to disease outcome.	Lungs	High
**NK cells**						
	Yes	Yes<br><br>(elicit IFN-γ production by CD8+ T cells; lyse Treg cells (13))	Yes	Exceptional cytotoxic mediated bacterial killing (47,48) is crucial to infection control.<br>NK cells are present in mature granulomas in the lungs of patients (23), and they have a role in the adaptive response (13) and disease control.	Lungs	High
**DCs**						
	Yes<br><br>(phagocytosis (38))	Yes<br><br>(antigen presentation (39))	Yes	Most efficient APCs, crucial for bridging the innate and adaptive immune responses (35,49). Without T cell response, infection control is hindered.	Lungs	High

Player	Involvement in immune defense arms (innate, adaptive, granuloma)			Influence on disease outcome	Site	Importance in immune response to *Mtb* (High/Fair/Low)
	Innate response	Adaptive response	Granuloma			
**CD4+ T cells**						
**Th1**	No	Yes	Yes	T cells are foremost in the adaptive response (40) and are a major cellular component of granuloma (50). They are known to play a vital role in the containment and progression of *Mtb* infection (50).	Lungs	High
**Th2**	No	Yes	Yes	T cells are foremost in the adaptive response (40) and are a major cellular component of granuloma (50). They are known to play a vital role in the containment and progression of *Mtb* infection (50).	Lungs	High
**Th17**	No	Yes	Yes	T cells are foremost in the adaptive response (40) and are a major cellular component of granuloma (50). They are known to play a vital role in the containment and progression of *Mtb* infection (50).	Lungs	High
**iTreg**	No	Yes	Yes	T cells are foremost in the adaptive response (40) and are a major cellular component of granuloma (50). They are known to play a vital role in the containment and progression of *Mtb* infection (50).	Lungs	High

Player	Involvement in immune defense arms (innate, adaptive, granuloma)			Influence on disease outcome	Site	Importance in immune response to *Mtb* (High/Fair/Low)
	Innate response	Adaptive response	Granuloma			
**CD8+ T cells**						
**Tc1**	No	Yes	Yes	Exhibit extremely strong cytotoxic activity in the adaptive response (including the granuloma), thereby affecting disease outcome (21).	Lungs	High
**Tc2**	No	Yes	Undetermined	Undetermined	Lungs	Fair
**B cells**						
	Undetermined	Yes	Yes	While the protective potential of B cell activity and the antibodies they produce against *Mtb* is debatable (40), it is thought that they do play a role in controlling disease outcome (40,51).	Airways/ Lungs/ Lymph nodes	Fair

DCs = dendritic cells; Ig = immunoglobulin; IFN = interferon; IL = interleukin; ILC = innate lymphoid cells; IP = interferon gamma-induced protein; iTreg = induced regulatory T cell; MAIT = mucosal-associated invariant T cells; Th = T helper cell; Tc = cytotoxic T cell; NK = natural killer; NKT = natural killer T cell.

Supplementary Table 3: Summary of the roles and significance ranking of molecular mediators/effectors in human TB

Player	Key associated mediators/peptides/ molecules	Role (e.g., cell recruitment/chemotaxis)	Site	Importance in immune response to *Mtb* (High/Fair/Low)
**Airway epithelium**				
**Airway epithelial cells**	IFN-γ	Recruitment and communication with immune cells (13).<br>Contributes to the initiation of innate immune responses (antimicrobial effect) (13).<br>Modulates the function of intraepithelial DCs (26).	Airways/Lungs	High
	TNF-α	Recruitment and communication with immune cells (13).<br>Contributes to the initiation of innate immune responses (antimicrobial effect) (13).<br>Modulates the function of intraepithelial DCs (26).	Airways/Lungs	High
	IL-10	Communication with immune cells (13).<br>Contributes to innate immune responses (13).	Airways/Lungs	High
	Antimicrobial peptides (cathelicidin [LL-37], β-defensin-2, and hepcidin)	Recruitment and communication with immune cells (13).<br>Contribute to the initiation of innate immune responses (antimicrobial effect) (13).	Airways/Lungs	High
	IL-6	Recruitment and communication with immune cells (13).<br>Contributes to the initiation of innate immune responses (antimicrobial effect) (13).<br>Modulates the function of intraepithelial DCs (26).	Airways/Lungs	Fair
	GM-CSF	Recruitment and communication with immune cells (13).<br>Contributes to the initiation of innate immune responses (antimicrobial effect) (13).<br>Modulates the function of intraepithelial DCs (26).	Airways/Lungs	Fair

Player	Key associated mediators/peptides/ molecules	Role (e.g., cell recruitment/chemotaxis)	Site	Importance in immune response to *Mtb* (High/Fair/Low)
	IL-8	Recruitment and communication with immune cells (13). Contributes to the initiation of innate immune responses (antimicrobial effect) (13). Modulates the function of intraepithelial DCs (26).	Airways/Lungs	Fair
	IP-10 (CXCL10)	Recruitment and communication with immune cells (13). Contributes to the initiation of innate immune responses (antimicrobial effect) (13). Modulates the function of intraepithelial DCs (26).	Airways/Lungs	Fair
	IL-27	Recruitment and communication with immune cells (13). Contributes to the initiation of innate immune responses (antimicrobial effect) (13). Modulates the function of intraepithelial DCs (26).	Airways/Lungs	Fair
	MCP-1 (CCL2)	Recruitment and communication with immune cells (13). Contributes to the initiation of innate immune responses (antimicrobial effect) (13). Modulates the function of intraepithelial DCs (26).	Airways/Lungs	Fair
	MIG	Recruitment and communication with immune cells (13). Contributes to the initiation of innate immune responses (antimicrobial effect) (13).	Airways/Lungs	Fair
	ROS	Recruitment and communication with immune cells (13). Contributes to the initiation of innate immune responses (antimicrobial effect) (13).	Airways/Lungs	Fair
	RNS	Recruitment and communication with immune cells (13). Contributes to the initiation of innate immune responses (antimicrobial effect) (13).	Airways/Lungs	Fair

Player	Key associated mediators/peptides/ molecules	Role (e.g., cell recruitment/chemotaxis)	Site	Importance in immune response to *Mtb* (High/Fair/Low)
	Gro-α (CXCL1)	Promotes neutrophil migration from the bloodstream to peripheral tissue (27).	Airways/Lungs	Fair
	ENA-78 (CXCL5)	Promotes neutrophil migration from the bloodstream to peripheral tissue (27).	Airways/Lungs	Fair
**Goblet cells**	Airway mucus (defensins, immunoglobulins, lysozymes, and cytokines)	Bacterial clearance via the mucociliary escalator (12).	Airways/Lungs	Low
**Plasma cells**	IgA	Specific for diverse *Mtb* surface antigens that can block *Mtb* infection of lung epithelial cells (12).	Airways/Lungs	Fair
**ILCs**	Same as Th1, Th2, and Th17 cells	Same as Th1, Th2, and Th17 cells (12).	Airways/Lungs	Low
**MAIT cells**	IFN-γ	Innate responses to intracellular pathogens (14).	Airways/Lungs	Low
	TNF-α	Innate responses to intracellular pathogens (14).	Airways/Lungs	Low
	IL-17	innate responses to intracellular pathogens (14).	Airways/Lungs	Low
	Granzymes	Cytotoxic capabilities (14).	Airways/Lungs	Low
**NKT cells**	IL-21	Facilitates the production of immunoglobulins by B cells (12).	Airways/Lungs	Low
**γδ T cells**	IFN-γ	Provides a protective effect for the host and may influence the formation of granulomas (12).	Airways/Lungs	Fair
	TNF-α	Provides a protective effect for the host and may influence the formation of granulomas (12).	Airways/Lungs	Fair

Player	Key associated mediators/peptides/ molecules	Role (e.g., cell recruitment/chemotaxis)	Site	Importance in immune response to *Mtb* (High/Fair/Low)
	IL-17	Provides a protective effect for the host and may influence the formation of granulomas (12).	Airways/Lungs	Fair
**Alveolar epithelium**				
**Type I and II pneumocytes**	Immunoglobulins, antimicrobial peptides, surfactant proteins	Bactericidal effects (12).<br>Surfactant proteins enhance the antimicrobial activity of macrophages, neutrophils, and lymphocytes (12).	Lungs	Fair
	TGF-β	Important immunoregulatory role in maintaining epithelial integrity and preventing immune-mediated destruction by limiting inflammation (13).	Lungs	Fair
**Type II pneumocytes**	CXCL5	Neutrophil recruitment to site of infection (20).	Lungs	Fair
	Hydrolases	Enhance neutrophil intracellular killing (12).	Lungs	High
**Macrophages**				
**M1**	IL-12	Th1 differentiation (42).<br>Possibly Tc1 differentiation (21).<br>Regulates the ongoing immune response, primarily by inducing differentiation of Th0 lymphocytes to Th1 lymphocytes, but also by enhancing the production of IFN-γ (52).<br>Cross-talk to increase IFN-γ production by Th1 cells (52).	Lungs	High
	TNF-α	Cell recruitment (53)and apoptosis (19,54).<br>Granuloma formation and function (43,55).	Lungs	High
	GM-CSF	Chemotactic gradient to recruit immune cells to the site of infection (27).	Lungs	Low

Player	Key associated mediators/peptides/ molecules	Role (e.g., cell recruitment/chemotaxis)	Site	Importance in immune response to *Mtb* (High/Fair/Low)
	IL-1β	Alters the metabolic pathway of AMs, shifting towards glycolysis (56).<br>T cell differentiation (36).	Lungs	High
	IFN-α/β (type I IFNs)	Enhance NK cell activation and effector functions (18).<br>Increase DC maturation, thus providing a more efficient antigen presentation and co-stimulation to T cells (18).<br>Act on T regulatory cells by dampening their suppressive capabilities (18).<br>Induce Th1 differentiation (18).<br>Type I IFNs sensed by CD4+ T cells protect them from NK cell-mediated killing (18).<br>During chronic infections, type I IFN release creates an immunosuppressive environment by increasing IL-10 expression by DCs, which induces CD4+ T cell activity exhaustion (18).	Lungs	High
	IL-6	Th17 differentiation (36).	Lungs	High
	IL-23	Induces Th17 differentiation (36)/effector function (41).	Lungs	Fair
	IL-8	Chemotactic gradient to recruit immune cells to the site of infection (27,32).	Lungs	High
	MIP2 (CCL2)	Chemotactic gradient to recruit immune cells to the site of infection (27,32).	Lungs	Fair
	IP-10 (CXCL10)	Chemotactic gradient to recruit immune cells to the site of infection (32).	Lungs	Fair

Player	Key associated mediators/peptides/ molecules	Role (e.g., cell recruitment/chemotaxis)	Site	Importance in immune response to *Mtb* (High/Fair/Low)
	MCP-1	Chemotactic gradient to recruit immune cells to the site of infection (32).	Lungs	Fair
	LTB4	Promotes neutrophil migration from the bloodstream to peripheral tissue (27).	Lungs	Low
	Gro-α (CXCL1)	Promotes neutrophil migration from the bloodstream to peripheral tissue (27).	Lungs	Fair
	IFN-α/β (type I interferons)	Enhances DC maturation, thereby improving antigen presentation capabilities to T cells (18).	Lungs	High
	ROS	Kills bacteria (33).	Lungs	High
	NO	Kills bacteria (33).	Lungs	High
**M2**	IL-10	Inhibitory function - immune modulation (prevent necrosis/apoptosis) (57).<br>Macrophages are the primary source of IL-10 (57).	Lungs	High
	TGF-β	Anti-inflammatory effects (12).	Lungs	Fair
	PGE2	Downregulates pro-inflammatory cytokine production in phagocytosing macrophages (27).	Lungs	Low
	ROS	Kills bacteria (33).	Lungs	High
	NO	Kills bacteria (33).	Lungs	High

Player	Key associated mediators/peptides/ molecules	Role (e.g., cell recruitment/chemotaxis)	Site	Importance in immune response to *Mtb* (High/Fair/Low)
**Neutrophils**				
	IFN-γ	Recruits and activates other immune cells (13).	Lungs	High
	TNF-α	Recruits and activates other immune cells (13).	Lungs	High
	IL-1β	Possibly cross-priming of CD8+ T cells (28).	Lungs	High
	IL-10	Neutrophil effector functions are negatively modulated by IL-10 (19).<br>Reduces bacterial killing and impairs the secretion of cytokines and chemokines (19).	Lungs	High
	IP-10 (CXCL10)	Recruits and activates other immune cells (13).	Lungs	Fair
	MCP-1 (CCL2)	Recruits and activates other immune cells (13).	Lungs	Fair
	MIP-1α/β	Recruits and activates other immune cells (13).	Lungs	Fair
	IL-12	Possibly cross-priming of CD8+ T cells (28).	Lungs	Fair
	VEGF	Possibly cross-priming of CD8+ T cells (28).	Lungs	Low
	Elastase, collagenase, and myeloperoxidase	Kill bacteria and indiscriminately damage bacterial and host cells (23).	Lungs	Fair
	Lipocalin 2	Antimicrobial peptide for bacterial restriction (29).	Lungs	Low
	Antimicrobial enzymes (α-defensins, matrix metalloproteases, lactoferin, and lipocalin)	Restrict the growth of mycobacteria within macrophages and promote apoptosis of infected macrophages (13).	Lungs	Fair

Player	Key associated mediators/peptides/ molecules	Role (e.g., cell recruitment/chemotaxis)	Site	Importance in immune response to *Mtb* (High/Fair/Low)
	NETs	Captures and eradicates microbes (30).	Lungs	Fair
**NK cells**				
	IFN-γ	Inhibits *Mtb* intracellular growth by enhancing phagolysosomal fusion (23).	Lungs	High
	IL-22	Inhibits *Mtb* intracellular growth by enhancing phagolysosomal fusion (23).	Lungs	Low
	Perforin	Cytotoxic activity (31).	Lungs	High
	Granulysin	Cytotoxic activity (31).	Lungs	High
	Granzymes	Cytotoxic activity (31).	Lungs	High
	TNF-α	Promotes γδ T cell proliferation (13,23).	Lungs	Low
**DCs**				
	IL-12p70	DC migration to DLN (19,58) in conjunction with CCL19 and CCL21 (59).<br>Induces Th1 differentiation (36).	Lungs/Lymph nodes	High
	IFN-α/β (type I interferons)	Increase DC maturation, thus providing a more efficient antigen presentation and co-stimulation to T cells (18).<br>Induce Th1 differentiation (18).	Lymph nodes	High
	IL-6	Induces Th17 differentiation (36).	Lymph nodes	High
	TGF-β	Induces Th17 differentiation (16).<br>iTreg differentiation (16).	Lymph nodes	High

**Player**	**Key associated mediators/peptides/ molecules**	**Role (e.g., cell recruitment/chemotaxis)**	**Site**	**Importance in immune response to *Mtb* (High/Fair/Low)**
	IL-1β	Induces Th17 differentiation (36).	Lymph nodes	High
	IL-10	Inhibits all Th17 cell differentiation (16) and chemotaxis to lungs (19).	Lymph nodes	High
	IL-23	Induces Th17 differentiation (16)/effector function (36).	Lymph nodes	Low
	IL-27	Inhibits Th17 differentiation (16).	Lymph nodes	Low
	IL-35	Stimulates the development of adaptive Treg cells (15).<br>Inhibits T-cell proliferation to limit tissue damage (37).	Lymph nodes	Low
**CD4+ T cells**				
**Th1**	IFN-γ	IFN-γ activates macrophages, which is critical for elimination of intracellular pathogens such as *Mtb* (42).	Lungs	High
	TNF-α	Recruits immune cells (mainly CD4+ and CD8+ T cells) to the site of infection and activate the effector functions in these cells (53).<br>TNF-α ultimately induces apoptosis of infected macrophages (53).	Lungs	High
	IL-10	Promotes immune suppression (57).	Lungs	High
**Th2**	IL-4	Considered to be the prototypical Th2 cell cytokine (39).<br>Positive feedback loop to promote further Th2 cell differentiation (41).<br>Promotes immune suppression (34).<br>Decreases cellular responsiveness to IFN-γ (34).<br>Inhibits the synthesis of iNOS (34).<br>Mediates IgE class switching in B cells (41).<br>Possibly Tc2 differentiation (CD8+ T cell) (21).	Lungs	High

Player	Key associated mediators/peptides/ molecules	Role (e.g., cell recruitment/chemotaxis)	Site	Importance in immune response to *Mtb* (High/Fair/Low)
	IL-10	Promotes immune suppression (39).	Lungs	High
	IL-13	Promotes immune suppression (34).<br>Decrease cellular responsiveness to IFN-γ (34).<br>Inhibit the synthesis of iNOS (34).	Lungs	Fair
	IL-5	Recruits eosinophils (41).	Lungs	Low
**Th17**	IL-17A	Promotes inflammatory responses including neutrophilia, tissue remodeling, and production of antimicrobial proteins (16).	Lungs	Fair
	IL-17F	Promotes inflammatory responses including neutrophilia, tissue remodeling, and production of antimicrobial proteins (16).	Lungs	Fair
	IL-21	Promotes inflammatory responses (41).	Lungs	Fair
	IL-22	Inhibits *Mtb* intracellular growth by enhancing phagolysosomal fusion (23).	Lungs	Low
**iTreg**	IL-10 (by Tr1 cell subset (15))	Promotes immune suppression (15,17).	Lungs	High
	TGF-β (by Th3 cell subset)	Promotes immune suppression (15,17).	Lungs	High
	IL-35	Stimulates the development of adaptive Treg cells (15).<br>Inhibits T cell proliferation to limit tissue damage (37).	Lungs	Fair
**CD8+ T cells**				
**Tc1**	IFN-γ	Activates resting macrophages, enhancing their ability to effectively clear pathogens and release cytokines (52).	Lungs	High

Player	Key associated mediators/peptides/ molecules	Role (e.g., cell recruitment/chemotaxis)	Site	Importance in immune response to *Mtb* (High/Fair/Low)
	TNF-α	Macrophage and T cell recruitment, macrophage activation (along with IFN-γ and bacterial signal), and induction of apoptosis in infected macrophages (60).	Lungs	High
	Perforin	Cytotoxic activity (14).	Lungs	High
	Granzyme B	Cytotoxic activity (14).	Lungs	High
	Granulysin	Cytotoxic activity (14).	Lungs	High
**Tc2**	IL-4	Tc2 differentiation (CD8+ T cell) (21).<br>Immune suppression (34).	Lungs	Fair
	IL-5	Recruits eosinophils (41).	Lungs	Fair
	IL-13	Immune suppression (24,34).	Lungs	Fair
**B cells**				
	IgG	Produced by Th1 interaction (19).<br>Opsonization (12).	Lungs	Low
	IgA	Produced by Th2 interaction (19).<br>Neutralization (12).	Lungs	Low
	IgE	Produced by Th17 interaction (19).<br>Sensitization of mast cells (41).	Lungs	Low
	IgM	B cell interaction with *Mtb* (19).<br>Activates complement system (61).	Lymph nodes	Low

AM = alveolar macrophage; CCL = CC chemokine ligand; CXCL = CXC chemokine ligand; DCs = dendritic cells; Ig = immunoglobulin; IFN = interferon; IL = interleukin; ILC = innate lymphoid cells; iNOS = inducible nitric oxide synthase; IP = interferon gamma-induced protein; iTreg = induced regulatory T cell; LTB4 = leukotriene B4; MAIT = mucosal-associated invariant T cells; MCP = monocyte chemoattractant protein; MIG = monokine induced by gamma interferon; MIP = macrophage inflammatory

protein; NO = nitric oxide; RNS = reactive nitrogen species; PD-L1 = programmed death-ligand 1; PGE 2 = prostaglandin E 2; ROS = reactive oxygen species; Th = T helper cell; Tc = cytotoxic T cell; NETs = neutrophil extracellular traps; NK = natural killer; NKT = natural killer T cells; TGF = tumor growth factor; VEGF = vascular endothelial growth factor.

11 Modeling & Simulation literature

Supplementary Table 4 displays the Modeling and Simulation literature (categorized by data type provided) that may be used for model development. These references were found using a keyword search (n = 43) as shown in **Figure 1**.

Supplementary Table 4: References containing data to support (semi-)mechanistic model development

Reference	Data type provided
Linderman JJ, Kirschner DE. In silico models of M. tuberculosis infection provide a route to new therapies. *Drug Discov Today Dis Models.* (2015) 15:37–41. doi: 10.1016/j.ddmod.2014.02.006	Treatment
Pienaar E, Dartois V, Linderman JJ, Kirschner DE. In silico evaluation and exploration of antibiotic tuberculosis treatment regimens. *BMC Syst Biol.* (2015) 9:79. doi: 10.1186/s12918-015-0221-8	Treatment
Magombedze G, Garira W, Mwenje E. Modelling the human immune response mechanisms to mycobacterium tuberculosis infection in the lungs. *Math Biosci Eng.* (2006) 3:661–682. doi: 10.3934/mbe.2006.3.661	Bacteria; CMI
Fors J, Strydom N, Fox WS, Keizer RJ, Savic RM. Mathematical model and tool to explore shorter multi-drug therapy options for active pulmonary tuberculosis. *PLoS Comput Biol.* (2020) 16:e1008107. doi: 10.1371/journal.pcbi.1008107	Treatment
Apiyo D, Mouton JM, Louw C, Sampson SL, Louw TM. Dynamic mathematical model development and validation of in vitro Mycobacterium smegmatis growth under nutrient- and pH-stress. *J Theor Biol.* (2022) 532:110921. doi: 10.1016/j.jtbi.2021.110921	Bacteria
Bru A, Cardona P-J. Mathematical modeling of tuberculosis bacillary counts and cellular populations in the organs of infected mice. *PLoS One.* (2010) 5:e12985. doi: 10.1371/journal.pone.0012985	Bacteria; granuloma
Ganguli S, Gammack D, Kirschner DE. A metapopulation model of granuloma formation in the lung during infection with mycobacterium tuberculosis. *Math Biosci Eng.* (2005) 2:535–560. doi: 10.3934/mbe.2005.2.535	Granuloma
Gong C, Linderman JJ, Kirschner D. A population model capturing dynamics of tuberculosis granulomas predicts host infection outcomes. *Math Biosci Eng.* (2015) 12:625–642. doi: 10.3934/mbe.2015.12.625	Granuloma
Ibarguen-Mondragon E, Esteva L, Burbano-Rosero EM. Mathematical model for the growth of Mycobacterium tuberculosis in the granuloma. *Math Biosci Eng.* (2018) 15:407–428. doi: 10.3934/mbe.2018018	Granuloma
Joslyn LR, Pienaar E, DiFazio RM, Suliman S, Kagina BM, Flynn JL, et al. Integrating Non-human Primate, Human, and Mathematical Studies to Determine the Influence of BCG Timing on H56 Vaccine Outcomes. *Front Microbiol.* (2018) 9:1734. doi: 10.3389/fmicb.2018.01734	Treatment

Reference	Data type provided
Kirschner D, Pienaar E, Marino S, Linderman JJ. A review of computational and mathematical modeling contributions to our understanding of Mycobacterium tuberculosis within-host infection and treatment. *Curr Opin Syst Biol.* (2017) 3:170–185. doi: 10.1016/j.coisb.2017.05.014	Treatment; CMI
Pedruzzi G, Das PN, Rao KVS, Chatterjee S. Understanding PGE2, LXA4 and LTB4 balance during Mycobacterium tuberculosis infection through mathematical model. *J Theor Biol.* (2016) 389:159–170. doi: 10.1016/j.jtbi.2015.10.025	Immune mediators
Renardy M, Kirschner DE. Evaluating vaccination strategies for tuberculosis in endemic and non-endemic settings. *J Theor Biol.* (2019) 469:1–11. doi: 10.1016/j.jtbi.2019.02.020	Treatment
Singer BH, Kirschner DE. Influence of backward bifurcation on interpretation of r(0) in a model of epidemic tuberculosis with reinfection. *Math Biosci Eng.* (2004) 1:81–93. doi: 10.3934/mbe.2004.1.81	Epidemiology
Murphy BM, Singer BH, Anderson S, Kirschner D. Comparing epidemic tuberculosis in demographically distinct heterogeneous populations. *Math Biosci.* (2002) 180:161–185. doi: 10.1016/s0025-5564(02)00133-5	Epidemiology
Chang ST, Linderman JJ, Kirschner DE. Multiple mechanisms allow Mycobacterium tuberculosis to continuously inhibit MHC class II-mediated antigen presentation by macrophages. *Proc Natl Acad Sci U S A.* (2005) 102:4530–4535. doi: 10.1073/pnas.0500362102	CMI
Cilfone NA, Ford CB, Marino S, Mattila JT, Gideon HP, Flynn JL, et al. Computational modeling predicts IL-10 control of lesion sterilization by balancing early host immunity-mediated antimicrobial responses with caseation during mycobacterium tuberculosis infection. *J Immunol.* (2015) 194:664–677. doi: 10.4049/jimmunol.1400734	Immune mediators
Cilfone NA, Perry CR, Kirschner DE, Linderman JJ. Multi-scale modeling predicts a balance of tumor necrosis factor-α and interleukin-10 controls the granuloma environment during Mycobacterium tuberculosis infection. *PLoS One.* (2013) 8:e68680. doi: 10.1371/journal.pone.006868	Immune mediators; Granuloma
Gammack D, Doering CR, Kirschner DE. Macrophage response to Mycobacterium tuberculosis infection. *J Math Biol.* (2004) 48:218–242. doi: 10.1007/s00285-003-0232-8	CMI
Guzzetta G, Kirschner D. The roles of immune memory and aging in protective immunity and endogenous reactivation of tuberculosis. *PLoS One.* (2013) 8:e60425. doi: 10.1371/journal.pone.0060425	Epidemiology
Guzzetta G, Ajelli M, Yang Z, Merler S, Furlanello C, Kirschner D. Modeling socio-demography to capture tuberculosis transmission dynamics in a low burden setting. *J Theor Biol.* (2011) 289:197–205. doi: 10.1016/j.jtbi.2011.08.032	Epidemiology
Guzzetta G, Ajelli M, Yang Z, Mukasa LN, Patil N, Bates JH, et al. Effectiveness of contact investigations for tuberculosis control in Arkansas. *J Theor Biol.* (2015) 380:238–246. doi: 10.1016/j.jtbi.2015.05.031	Epidemiology

**Reference**	**Data type provided**
Kirschner D. Dynamics of co-infection with M. Tuberculosis and HIV-1. *Theor Popul Biol.* (1999) 55:94–109. doi: 10.1006/tpbi.1998.1382	Co-infection
Linderman JJ, Cilfone NA, Pienaar E, Gong C, Kirschner DE. A multi-scale approach to designing therapeutics for tuberculosis. *Integr Biol (Camb).* (2015) 7:591–609. doi: 10.1039/c4ib00295d	Treatment
Marino S, Hult C, Wolberg P, Linderman JJ, Kirschner DE. The Role of Dimensionality in Understanding Granuloma Formation. *Computation (Basel).* (2018) 6: doi: 10.3390/computation6040058	Granuloma
Marino S, Kirschner DE. The human immune response to Mycobacterium tuberculosis in lung and lymph node. *Journal of Theoretical Biology.* (2004) 227:463–486. doi: 10.1016/j.jtbi.2003.11.023	CMI
Marino S, Kirschner DE. A Multi-Compartment Hybrid Computational Model Predicts Key Roles for Dendritic Cells in Tuberculosis Infection. *Computation (Basel).* (2016) 4: doi: 10.3390/computation4040039	CMI
Marino S, Linderman JJ, Kirschner DE. A multifaceted approach to modeling the immune response in tuberculosis. *Wiley Interdiscip Rev Syst Biol Med.* (2011) 3:479–489. doi: 10.1002/wsbm.131	CMI
Marino S, Pawar S, Fuller CL, Reinhart TA, Flynn JL, Kirschner DE. Dendritic cell trafficking and antigen presentation in the human immune response to Mycobacterium tuberculosis. *J Immunol.* (2004) 173:494–506. doi: 10.4049/jimmunol.173.1.494	CMI
Millar JA, Butler JR, Evans S, Mattila JT, Linderman JJ, Flynn JL, et al. Spatial Organization and Recruitment of Non-Specific T Cells May Limit T Cell-Macrophage Interactions Within Mycobacterium tuberculosis Granulomas. *Front Immunol.* (2020) 11:613638. doi: 10.3389/fimmu.2020.613638	CMI
Repasy T, Lee J, Marino S, Martinez N, Kirschner DE, Hendricks G, et al. Intracellular bacillary burden reflects a burst size for Mycobacterium tuberculosis in vivo. *PLoS Pathog.* (2013) 9:e1003190. doi: 10.1371/journal.ppat.1003190	Bacteria
Sud D, Bigbee C, Flynn JL, Kirschner DE. Contribution of CD8+ T cells to control of Mycobacterium tuberculosis infection. *J Immunol* (2006) 176:4296–4314. doi: 10.4049/jimmunol.176.7.4296	CMI
Wigginton JE, Kirschner D. A model to predict cell-mediated immune regulatory mechanisms during human infection with Mycobacterium tuberculosis. *J Immunol.* (2001) 166:1951–1967. doi: 10.4049/jimmunol.166.3.1951	CMI
Wong EA, Evans S, Kraus CR, Engelman KD, Maiello P, Flores WJ, et al. IL-10 Impairs Local Immune Response in Lung Granulomas and Lymph Nodes during Early Mycobacterium tuberculosis Infection. *J Immunol.* (2020) 204:644–659. doi: 10.4049/jimmunol.1901211	Granuloma

Reference	Data type provided
Wong EA, Joslyn L, Grant NL, Klein E, Lin PL, Kirschner DE, Flynn JL. Low Levels of T Cell Exhaustion in Tuberculous Lung Granulomas. *Infect Immun.* (2018) 86: doi: 10.1128/IAI.00426-18	CMI; Granuloma
Evans S, Butler JR, Mattila JT, Kirschner DE. Systems biology predicts that fibrosis in tuberculous granulomas may arise through macrophage-to-myofibroblast transformation. *PLoS Comput Biol.* (2020) 16:e1008520. doi: 10.1371/journal.pcbi.1008520	Granuloma
Fallahi-Sichani M, Kirschner DE, Linderman JJ. NF-κB Signaling Dynamics Play a Key Role in Infection Control in Tuberculosis. *Front Physiol.* (2012) 3:170. doi: 10.3389/fphys.2012.00170	Signaling
Marino S, Cilfone NA, Mattila JT, Linderman JJ, Flynn JL, Kirschner DE. Macrophage polarization drives granuloma outcome during Mycobacterium tuberculosis infection. *Infect Immun.* (2015) 83:324–338. doi: 10.1128/IAI.02494-14	Granuloma
Marino S, Sud D, Plessner H, Lin PL, Chan J, Flynn JL, et al. Differences in reactivation of tuberculosis induced from anti-TNF treatments are based on bioavailability in granulomatous tissue. *PLoS Comput Biol.* (2007) 3:1909–1924. doi: 10.1371/journal.pcbi.0030194	Treatment; Granuloma
Pienaar E, Matern WM, Linderman JJ, Bader JS, Kirschner DE. Multiscale Model of Mycobacterium tuberculosis Infection Maps Metabolite and Gene Perturbations to Granuloma Sterilization Predictions. *Infect Immun.* (2016) 84:1650–1669. doi: 10.1128/IAI.01438-15	Granuloma
Warsinske HC, DiFazio RM, Linderman JJ, Flynn JL, Kirschner DE. Identifying mechanisms driving formation of granuloma-associated fibrosis during Mycobacterium tuberculosis infection. *J Theor Biol.* (2017) 429:1–17. doi: 10.1016/j.jtbi.2017.06.017	Granuloma
Warsinske HC, Pienaar E, Linderman JJ, Mattila JT, Kirschner DE. Deletion of TGF-β1 Increases Bacterial Clearance by Cytotoxic T Cells in a Tuberculosis Granuloma Model. *Front Immunol.* (2017) 8:1843. doi: 10.3389/fimmu.2017.01843	Granuloma
Wessler T, Joslyn LR, Borish HJ, Gideon HP, Flynn JL, Kirschner DE, et al. A computational model tracks whole-lung Mycobacterium tuberculosis infection and predicts factors that inhibit dissemination. *PLoS Comput Biol*. (2020) 16:e1007280. doi: 10.1371/journal.pcbi.1007280	Granuloma

CMI = cell-mediated immunity.

12 References

1. Goletti D, Lindestam Arlehamn CS, Scriba TJ, Anthony R, Cirillo DM, Alonzi T,et al.. Can we predict tuberculosis cure? What tools are available? *Eur Respir J.* (2018) 52:1801089. doi: 10.1183/13993003.01089-2018

2. Marino S, Kirschner DE. A Multi-Compartment Hybrid Computational Model Predicts Key Roles for Dendritic Cells in Tuberculosis Infection. *Computation (Basel).* (2016) 4: doi: 10.3390/computation4040039

3. Sotgiu G, Centis R, D'ambrosio L, Migliori GB. Tuberculosis treatment and drug regimens. *Cold Spring Harb Perspect Med.* (2015) 5:a017822. doi: 10.1101/cshperspect.a017822

4. Bartelink I, Zhang N, Keizer R, Strydom N, Converse P, Dooley K, et al. New Paradigm for Translational Modeling to Predict Long-term Tuberculosis Treatment Response: Translational Modeling Predicts TB Treatment Response. *Clin Transl Science.* (2017) 10:366–379. doi: 10.1111/cts.12472

5. Singh V, Chibale K. Strategies to Combat Multi-Drug Resistance in Tuberculosis. *Acc Chem Res.* (2021) 54:2361–2376. doi: 10.1021/acs.accounts.0c00878

6. World Health Organization. *WHO treatment guidelines for isoniazid-resistant tuberculosis: supplement to the WHO treatment guidelines for drug-resistant tuberculosis*. Geneva: World Health Organization. (2018). https://iris.who.int/handle/10665/260494 [Accessed July 25, 2024].

7. Viney K, Linh NN, Gegia M, Zignol M, Glaziou P, Ismail N, et al. New definitions of pre-extensively and extensively drug-resistant tuberculosis: update from the World Health Organization. *Eur Respir J.* (2021) 57:2100361. doi: 10.1183/13993003.00361-2021

8. Abrahams KA, Besra GS. Mycobacterial drug discovery. *RSC Med Chem.* (2020) 11:1354–1365. doi: 10.1039/D0MD00261E

9. Dooley KE, Phillips PPJ, Nahid P, Hoelscher M. Challenges in the clinical assessment of novel tuberculosis drugs. *Adv Drug Delivery Rev.* (2016) 102:116–122. doi: 10.1016/j.addr.2016.01.014

10. Kirschner D, Pienaar E, Marino S, Linderman JJ. A review of computational and mathematical modeling contributions to our understanding of Mycobacterium tuberculosis within-host infection and treatment. *Curr Opin Syst Biol*. (2017) 3:170–185. doi: 10.1016/j.coisb.2017.05.014

11. Clewe O, Faraj A, Hu Y, Coates ARM, Simonsson USH. A model-based analysis identifies differences in phenotypic resistance between in vitro and in vivo: implications for translational medicine within tuberculosis. *J Pharmacokinet Pharmacodyn.* (2020) 47:421–430. doi: 10.1007/s10928-020-09694-0

12. Chai Q, Lu Z, Liu CH. Host defense mechanisms against Mycobacterium tuberculosis. *Cell Mol Life Sci.* (2020) 77:1859–1878. doi: 10.1007/s00018-019-03353-5

13. Gupta N, Kumar R, Agrawal B. New Players in Immunity to Tuberculosis: The Host Microbiome, Lung Epithelium, and Innate Immune Cells. *Front Immunol.* (2018) 9:709. doi: 10.3389/fimmu.2018.00709

14. Lin PL, Flynn JL. CD8 T cells and Mycobacterium tuberculosis infection. *Semin Immunopathol.* (2015) 37:239–249. doi: 10.1007/s00281-015-0490-8

15. Lourenço EV, La Cava A. Natural regulatory T cells in autoimmunity. *Autoimmunity.* (2011) 44:33–42. doi: 10.3109/08916931003782155

16. Huang G, Wang Y, Chi H. Regulation of TH17 cell differentiation by innate immune signals. *Cell Mol Immunol.* (2012) 9:287–295. doi: 10.1038/cmi.2012.10

17. Dennis KL, Blatner NR, Gounari F, Khazaie K. Current status of interleukin-10 and regulatory T-cells in cancer. *Curr Opin Oncol.* (2013) 25:637–645. doi: 10.1097/CCO.0000000000000006

18. Kuka M, De Giovanni M, Iannacone M. The role of type I interferons in CD4+ T cell differentiation. *Immunol Lett.* (2019) 215:19–23. doi: 10.1016/j.imlet.2019.01.013

19. Pennisi M, Russo G, Sgroi G, Bonaccorso A, Parasiliti Palumbo GA, Fichera E, et al. Predicting the artificial immunity induced by RUTI® vaccine against tuberculosis using universal immune system simulator (UISS). *BMC Bioinformatics.* (2019) 20:504. doi: 10.1186/s12859-019-3045-5

20. Corleis B, Dorhoi A. Early dynamics of innate immunity during pulmonary tuberculosis. *Immunol Letters.* (2020) 221:56–60. doi: 10.1016/j.imlet.2020.02.010

21. St. Paul M, Ohashi PS. The Roles of CD8+ T Cell Subsets in Antitumor Immunity. *Trends Cell Biol.* (2020) 30:695–704. doi: 10.1016/j.tcb.2020.06.003

22. Kumar NP, Moideen K, Banurekha VV, Nair D, Babu S. Modulation of Th1/Tc1 and Th17/Tc17 responses in pulmonary tuberculosis by IL-20 subfamily of cytokines. *Cytokine.* (2018) 108:190–196. doi: 10.1016/j.cyto.2018.04.005

23. Liu CH, Liu H, Ge B. Innate immunity in tuberculosis: host defense vs pathogen evasion. *Cell Mol Immunol.* (2017) 14:963–975. doi: 10.1038/cmi.2017.88

24. Kudryavtsev I, Zinchenko Y, Serebriakova M, Akisheva T, Rubinstein A, Savchenko A, et al. A Key Role of CD8+ T Cells in Controlling of Tuberculosis Infection. *Diagnostics.* (2023) 13:2961. doi: 10.3390/diagnostics13182961

25. Mittrücker H-W, Visekruna A, Huber M. Heterogeneity in the Differentiation and Function of CD8+ T Cells. *Arch Immunol Ther Exp.* (2014) 62:449–458. doi: 10.1007/s00005-014-0293-y

26. Condon TV, Sawyer RT, Fenton MJ, Riches DWH. Lung dendritic cells at the innate-adaptive immune interface. *J Leukocyte Biol.* (2011) 90:883–895. doi: 10.1189/jlb.0311134

27. Lowe DM, Redford PS, Wilkinson RJ, O'Garra A, Martineau AR. Neutrophils in tuberculosis: friend or foe? *Trends Immunol.* (2012) 33:14–25. doi: 10.1016/j.it.2011.10.003

28. Mattila JT, Ojo OO, Kepka-Lenhart D, Marino S, Kim JH, Eum SY, et al. Microenvironments in tuberculous granulomas are delineated by distinct populations of macrophage subsets and expression of nitric oxide synthase and arginase isoforms. *J Immunol.* (2013) 191:773–784. doi: 10.4049/jimmunol.1300113

29. Borkute RR, Woelke S, Pei G, Dorhoi A. Neutrophils in Tuberculosis: Cell Biology, Cellular Networking and Multitasking in Host Defense. *IJMS.* (2021) 22:4801. doi: 10.3390/ijms22094801

30. Hilda JN, Das S, Tripathy SP, Hanna LE. Role of neutrophils in tuberculosis: A bird's eye view. *Innate Immun.* (2020) 26:240–247. doi: 10.1177/1753425919881176

31. Murphy K, Weaver C, Berg L, Janeway C. *Janeway's immunobiology*. *10th edition.* New York, NY: W.W. Norton and Company (2022).

32. Gammack D, Doering CR, Kirschner DE. Macrophage response to Mycobacterium tuberculosis infection. *J Math Biol.* (2004) 48:218–242. doi: 10.1007/s00285-003-0232-8

33. Weiss G, Schaible UE. Macrophage defense mechanisms against intracellular bacteria. *Immunol Rev.* (2015) 264:182–203. doi: 10.1111/imr.12266

34. Guirado E, Schlesinger LS, Kaplan G. Macrophages in tuberculosis: friend or foe. *Semin Immunopathol.* (2013) 35:563–583. doi: 10.1007/s00281-013-0388-2

35. Sia JK, Georgieva M, Rengarajan J. Innate Immune Defenses in Human Tuberculosis: An Overview of the Interactions between Mycobacterium tuberculosis and Innate Immune Cells. *J Immunol Res.* (2015) 2015:1–12. doi: 10.1155/2015/747543

36. Leal Rojas IM, Mok W-H, Pearson FE, Minoda Y, Kenna TJ, Barnard RT, et al. Human Blood CD1c+ Dendritic Cells Promote Th1 and Th17 Effector Function in Memory CD4+ T Cells. *Front Immunol.* (2017) 8:971. doi: 10.3389/fimmu.2017.00971

37. Herbert C, Ahmadzai H, Thomas PS. Proinflammatory and Regulatory Cytokines in Sarcoidosis. In: *Cytokine Effector Functions in Tissues*. London, UK: Elsevier (2017). p. 129–38. Available at: https://linkinghub.elsevier.com/retrieve/pii/B9780128042144000075.

38. Marino S, Pawar S, Fuller CL, Reinhart TA, Flynn JL, Kirschner DE. Dendritic cell trafficking and antigen presentation in the human immune response to Mycobacterium tuberculosis. *J Immunol.* (2004) 173:494–506. doi: 10.4049/jimmunol.173.1.494

39. Marino S, Kirschner DE. The human immune response to Mycobacterium tuberculosis in lung and lymph node. *J Theor Biol.* (2004) 227:463–486. doi: 10.1016/j.jtbi.2003.11.023

40. Flynn JL, Chan J. Immune cell interactions in tuberculosis. *Cell.* (2022) 185:4682–4702. doi: 10.1016/j.cell.2022.10.025

41. Carty SA, Riese MJ, Koretzky GA. Chapter 21 - T-Cell Immunity. In: Hoffman R, Benz EJ, Silberstein LE, Heslop HE, Weitz JI, Anastasi J, et al, editors. *Hematology, Seventh Edition*. Philadephia, USA: Elsevier (2018). p. 221–239 doi: 10.1016/B978-0-323-35762-3.00021-4.

42. Chaurasiya SK. Tuberculosis: Smart manipulation of a lethal host: Survival Inside Killers. *Microbiol Immunol.* (2018) 62:361–379. doi: 10.1111/1348-0421.12593

43. Russell DG, Cardona P-J, Kim M-J, Allain S, Altare F. Foamy macrophages and the progression of the human tuberculosis granuloma. *Nat Immunol.* (2009) 10:943–948. doi: 10.1038/ni.1781

44. Flynn JL, Chan J, Lin PL. Macrophages and control of granulomatous inflammation in tuberculosis. *Mucosal Immunol.* (2011) 4:271–278. doi: 10.1038/mi.2011.14

45. Silva Miranda M, Breiman A, Allain S, Deknuydt F, Altare F. The Tuberculous Granuloma: An Unsuccessful Host Defence Mechanism Providing a Safety Shelter for the Bacteria? *Clin Dev Immunol.* (2012) 2012:1–14. doi: 10.1155/2012/139127

46. Ehlers S, Schaible UE. The granuloma in tuberculosis: dynamics of a host-pathogen collusion. *Front Immunol.* (2012) 3:411. doi: 10.3389/fimmu.2012.00411

47. Nutt SL, Huntington ND. Cytotoxic T Lymphocytes and Natural Killer Cells. In: *Clinical Immunology*. Amsterdam, Netherlands: Elsevier (2019). p. 247–259.e1. Available at: https://linkinghub.elsevier.com/retrieve/pii/B978070206896600017X.

48. Nisa A, Kipper FC, Panigrahy D, Tiwari S, Kupz A, Subbian S. Different modalities of host cell death and their impact on *Mycobacterium tuberculosis* infection. *Am J Physiology-Cell Physiol.* (2022) 323:C1444–C1474. doi: 10.1152/ajpcell.00246.2022

49. Kim H, Shin SJ. Pathological and protective roles of dendritic cells in Mycobacterium tuberculosis infection: Interaction between host immune responses and pathogen evasion. *Front Cell Infect Microbiol.* (2022) 12:891878. doi: 10.3389/fcimb.2022.891878

50. Gideon HP, Phuah J, Myers AJ, Bryson BD, Rodgers MA, Coleman MT, et al. Variability in tuberculosis granuloma T cell responses exists, but a balance of pro- and anti-inflammatory cytokines is associated with sterilization. *PLoS Pathog.* (2015) 11:e1004603. doi: 10.1371/journal.ppat.1004603

51. Hunter L, Hingley-Wilson S, Stewart GR, Sharpe SA, Salguero FJ. Dynamics of Macrophage, T and B Cell Infiltration Within Pulmonary Granulomas Induced by

Mycobacterium tuberculosis in Two Non-Human Primate Models of Aerosol Infection. *Front Immunol.* (2022) 12:776913. doi: 10.3389/fimmu.2021.776913

52. Wigginton JE, Kirschner D. A model to predict cell-mediated immune regulatory mechanisms during human infection with Mycobacterium tuberculosis. *J Immunol.* (2001) 166:1951–1967. doi: 10.4049/jimmunol.166.3.1951

53. Guzzetta G, Kirschner D. The roles of immune memory and aging in protective immunity and endogenous reactivation of tuberculosis. *PLoS One.* (2013) 8:e60425. doi: 10.1371/journal.pone.0060425

54. Pedruzzi G, Das PN, Rao KVS, Chatterjee S. Understanding PGE2, LXA4 and LTB4 balance during Mycobacterium tuberculosis infection through mathematical model. *Theorl Biol.* (2016) 389:159–170. doi: 10.1016/j.jtbi.2015.10.025

55. Linderman JJ, Kirschner DE. In silico models of M. tuberculosis infection provide a route to new therapies. *Drug Discovery Today Dis Models.* (2015) 15:37–41. doi: 10.1016/j.ddmod.2014.02.006

56. Ryndak MB, Laal S. Mycobacterium tuberculosis Primary Infection and Dissemination: A Critical Role for Alveolar Epithelial Cells. *Front Cell Infect Microbiol.* (2019) 9:299. doi: 10.3389/fcimb.2019.00299

57. Cilfone NA, Ford CB, Marino S, Mattila JT, Gideon HP, Flynn JL, et al. Computational modeling predicts IL-10 control of lesion sterilization by balancing early host immunity-mediated antimicrobial responses with caseation during mycobacterium tuberculosis infection. *J Immunol.* (2015) 194:664–677. doi: 10.4049/jimmunol.1400734

58. Khader SA, Partida-Sanchez S, Bell G, Jelley-Gibbs DM, Swain S, Pearl JE, et al. Interleukin 12p40 is required for dendritic cell migration and T cell priming after Mycobacterium tuberculosis infection. *J Exp Med.* (2006) 203:1805–1815. doi: 10.1084/jem.20052545

59. Lian J, Luster AD. Chemokine-guided cell positioning in the lymph node orchestrates the generation of adaptive immune responses. *Curr Opin Cell Biol.* (2015) 36:1–6. doi: 10.1016/j.ceb.2015.05.003

60. Sud D, Bigbee C, Flynn JL, Kirschner DE. Contribution of CD8+ T cells to control of Mycobacterium tuberculosis infection. *J Immunol.* (2006) 176:4296–4314. doi: 10.4049/jimmunol.176.7.4296

61. Mak TW, Saunders ME. B Cell Receptor Structure and Effector Function. In: *The Immune Response*. Amsterdam, Netherlands; Boston, USA: Elsevier (2006). p. 93–120. Available at: https://linkinghub.elsevier.com/retrieve/pii/B9780120884513500077.
